# Characterization of the *Castanopsis carlesii* Deadwood Mycobiome by Pacbio Sequencing of the Full-Length Fungal Nuclear Ribosomal Internal Transcribed Spacer (ITS)

**DOI:** 10.3389/fmicb.2019.00983

**Published:** 2019-05-17

**Authors:** Witoon Purahong, Ausana Mapook, Yu-Ting Wu, Chaur-Tzuhn Chen

**Affiliations:** ^1^Department of Soil Ecology, UFZ-Helmholtz Centre for Environmental Research, Halle, Germany; ^2^Center of Excellence in Fungal Research, Mae Fah Luang University, Chiang Rai, Thailand; ^3^Department of Forestry, National Pingtung University of Science and Technology, Pingtung, Taiwan

**Keywords:** NGS, long-read sequencing, fungal community, wood-inhabiting fungi, fungal diversity, species identification, Taiwan

## Abstract

Short-read next generation sequencing (NGS) platforms can easily and quickly generate thousands to hundreds of thousands of sequences per sample. However, the limited length of these sequences can cause problems during fungal taxonomic identification. Here we validate the use of Pacbio sequencing, a long-read NGS method, for characterizing the fungal community (mycobiome) of *Castanopsis carlesii* deadwood. We report the successful use of Pacbio sequencing to generate long-read sequences of the full-length (500–780 bp) fungal ITS regions of the *C. carlesii* mycobiome. Our results show that the studied deadwood mycobiome is taxonomically and functionally diverse, with an average of 85 fungal OTUs representing five functional groups (animal endosymbionts, endophytes, mycoparasites, plant pathogens, and saprotrophs). Based on relative abundance data, Basidiomycota were the most frequently detected phyla (50% of total sequences), followed by unidentified phyla, and Ascomycota. However, based on presence/absence data, the most OTU-rich phyla were Ascomycota (58% of total OTUs, 72 OTUs) followed by Basidiomycota and unidentified phyla. The majority of fungal OTUs were identified as saprotrophs (70% of successfully function-assigned OTUs) followed by plant pathogens. Finally, we used phylogenetic analysis based on the full-length ITS sequences to confirm the species identification of 14/36 OTUs with high bootstrap support (99–100%). Based on the numbers of sequence reads obtained per sample, which ranged from 3,047 to 13,463, we conclude that Pacbio sequencing can be a powerful tool for characterizing moderate- and possibly high-complexity fungal communities.

## Introduction

Short-read next generation sequencing (NGS) platforms such as 454 pyrosequencing, Ion Torrent, and Illumina have been used extensively to characterize fungal communities (mycobiomes) in various spheres, including the atmosphere, and hydrosphere (Nawaz et al., 2016), biosphere (i.e., the sphere of plant-derived substrates such as leaves, roots, and wood) (Gao et al., 2013; Hoppe et al., 2016; Purahong et al., 2016, 2018a), lithosphere (i.e., soil) (Goldmann et al., 2015), and anthroposphere (i.e., land used by humans) (Vályi et al., 2015). These approaches can easily generate thousands to hundreds of thousands of sequences per sample in a short time, but the limited length of these sequences can complicate fungal taxonomic identification (Nilsson et al., 2009; Goodwin et al., 2016). The internal transcribed spacer (ITS) region has been identified as the sequence with the greatest potential for successfully identifying the widest possible range of fungal groups, and is therefore commonly used as a universal DNA barcode for fungi (Schoch et al., 2012). To compensate for the limitations of short-read NGS methods, species identification based on the ITS1, or ITS2 sub-regions rather than the full ITS region has been tested. It is not yet known which of these two sub-regions is best for this purpose, but it is clear that the choice of region can influence the success of fungal taxonomic identification. Nilsson et al. (2009) compared the performance of the two regions by analyzing 26,577 quality-filtered ITS sequences, showing that region choice affected taxonomic identification in ∼51% of the studied cases. Additionally, there was no agreement between taxonomic identifications based on ITS1, ITS2, and the full ITS region in 4% of cases at the genus level and 14% of cases at the species level. Accordingly, several recent studies have suggested that short-read NGS based on ITS1 (Jayawardena et al., 2018) or ITS2 (Dissanayake et al., 2018; Purahong et al., 2018b,c) sequences may only be sufficient for correct taxonomic identification at the genus or higher taxonomic levels.

Long-read NGS methods provide alternative ways to characterize the mycobiome and achieve more accurate fungal taxonomic identification (Tedersoo et al., 2018). One of the most widely used platforms for this purpose is the single-molecule real-time (SMRT) sequencing platform of Pacific Biosciences (PacBio) (Rhoads and Au, 2015). This platform’s usefulness was initially limited by its high error rate (approximately 13%) when using the single pass approach (Goodwin et al., 2016). However, the adoption of a multiple pass approach made it possible to generate a consensus read for an insert (also known as a circular consensus sequence, CCS), reducing the error rate to less than 1% (Goodwin et al., 2016). Unfortunately, the multiple pass approach may be unsuitable for use with DNA templates longer than ∼3 kb because it is difficult to sequence such templates repeatedly. However, shorter DNA templates (such as the fungal ITS region, which is typically <1000 bp) are readily sequenced multiple times, enabling the use of multiple pass Pacbio sequencing for highly accurate characterization of fungal communities. Although Pacbio sequencing can be used with longer sequences than short-read NGS methods such as Illumina and 454 pyrosequencing, it is not a high throughput method. For environmental samples such as soil- and plant-derived substrates, Illumina sequencing yields at least four to hundreds of thousands of high quality sequences per sample; the corresponding ranges for Pacbio sequencing are 2,138–43,618 for soil samples (Tedersoo et al., 2018) and 1,000 to ∼10,000 for plant material (Walder et al., 2017). The numbers of sequences obtained using Pacbio sequencing are thus highly variable and strongly dependent on the choice of primers. Consequently, some good sequences will be lost during normalization, when the number of sequences per sample is equalized to the number achieved for the sample with the fewest reads (Tedersoo et al., 2018). Although the number of obtained sequences can be used to roughly estimate the completeness of a sequencing method, absolute sequence numbers are not necessarily the best measure of performance. Instead, the goal should be to obtain a reasonable representation of the fungal community (which may sometimes be achievable with a relatively low sequence depth). It should also be noted that the per-sample cost of Pacbio sequencing greatly exceeds that of other short-read NGS methods (Quail et al., 2012). Therefore, Pacbio sequencing is currently a low-throughput approach that may be suitable for characterizing simple to moderately complex fungal communities.

Despite the potential of Pacbio sequencing for mycobiome analysis, it has not been widely used for this purpose (Tedersoo et al., 2018); only few suitable bioinformatics workflows or sequence quality filter parameters have been reported. Here we describe the use of Pacbio sequencing to characterize the fungal community associated with *Castanopsis carlesii* deadwood. Deadwood was chosen because it is important for diverse ecosystem functions (as a source of energy and nutrients) and geomorphological processes, and harbors moderately diverse fungi from various taxonomic and functional groups (Hodge and Peterken, 1998; Rajala et al., 2010, 2012; Stokland et al., 2012; Purahong et al., 2018a,b). Individual deadwood logs commonly harbor up to 100 fungal OTUs (the mean being between 20 and 45 OTUs), so a sequence depth of approximately 3000 is normally sufficient to obtain saturated or almost saturated rarefaction curves, and thus to assess fungal diversity with reasonable accuracy (Purahong et al., 2017, 2018b). *C. carlesii* was chosen because of its economic and ecological importance; it is one of the most abundant tree species in southeastern Taiwan. Moreover, the deadwood mycobiome of this tree species has not been characterized. We expected that phylogenetic analysis based on full-length ITS data obtained by Pacbio sequencing would confirm reported genus- and species-level taxonomic data for fungal classes such as Eurotiomycetes, Sordariomycetes, and Dothideomycetes, in which ITS is reportedly a good marker for species identification (Vu et al., 2019). We therefore conducted a study with the following goals: (i) assess the diversity of the *C. carlesii* deadwood mycobiome, (ii) identify the fungal taxonomic and functional groups associated with this mycobiome, (iii) evaluate the suitability of Pacbio sequencing for mycobiome analysis in moderately complex fungal communities, (iv) assess the potential of phylogenetic analysis based on full-length ITS data obtained by Pacbio sequencing for confirming genus- and species-level taxonomic assignments, and (v) compare the suitability and efficiency of long-read (full-length ITS) and short-read (ITS1 or ITS2) sequences for characterizing the deadwood mycobiome of *C. carlesii*.

## Materials and Methods

### Study Site and Sampling

The study was conducted on a 1-ha permanent site at Tajen Forest Station (22°27’N 120°82′E) in southeastern Taiwan. The forest at this site has been classified as a tropical forest. The site’s mean annual temperature and humidity are 25°C and 75%, respectively, with a mean annual precipitation of 2,400 mm (based on data from 1998 to 2006), falling mostly between April and October. A long-term experiment has been established at the 1-ha site, which has been divided into 20 plots subjected to five different thinning regimes (0, 20, 40, 60, and 80% cutting) since February 2015. The 1-ha study site harbors 83 vascular plant species, representing 61 genera and 31 families (Yang et al., 2015). The site’s most abundant tree species is *C. carlesii*, which is often found to have severe rotted heart-wood disease during thinning management. Wood chip samples were collected from freshly cut rotted heart-wood deadwood from three *C. carlesii* individuals (three samples were collected in total, one from each individual). Samples were collected using a cordless drill (Makita DDF 453) equipped with a wood auger (diameter 20 mm, length 135 mm), which was flamed and wiped with ethanol between drillings to avoid cross-contamination between samples. The samples were frozen in liquid nitrogen immediately after collection, transported to the laboratory on dry ice (ca. −70°C) as soon as possible, and stored at −20°C until analysis.

### DNA Extraction, ITS Amplicon Library Generation, and Sequencing

Each deadwood sample was separately homogenized into a fine powder. DNA was extracted from 100 mg of each homogenized wood sample using the Soil DNA Isolation Mini Kit (FAVORGEN, Taiwan) according to the manufacturer’s instructions. The presence and quantity of genomic DNA was checked using a NanoDrop ND-1000 spectrophotometer (Thermo Fisher Scientific, Dreieich, Germany) and the extracts were then stored at −20°C until needed for downstream PCR.

Fungal communities were characterized based on complete ITS sequences. Amplicon libraries were generated using the fungus-specific universal primer pair ITS1F/ITS4. Triplicate PCR amplifications of each sample were performed in a total volume of 50 μl containing 2.5U *Superrun EX taq*^TM^ HS, 5 μl of 10X *EX taq* Buffer, 200 μM of dNTPs, 0.2 μM of each primer, and 2–5 μg diluted template DNA. A touchdown PCR program was used involving an initial denaturation period (95°C for 5 min) followed by 10 cycles of denaturation at 94°C for 30 s, annealing at 60–50°C for 45 s (−1°C per cycle), and extension at 72°C for 2 min; and then 30 cycles of 94°C for 30 s, 50°C for 45 s and 72°C for 2 min, with a final 10 min extension step at 72°C. The PCR products were separated by 1 % agarose gel electrophoresis using 1X TAE buffer and SYBR^®^Green I. Bands of the expected size (between 500 and 1000bp) were cut out and purified using a QIAEX II Gel Extraction Kit (QIAGEN Inc., Valencia, United States). Sixteen-nucleotide barcoded SMRTbell adapters were added to the 5′ ends of amplicons and reverse complement barcoded adapters were added to the 3′ ends to enable multiplexing of samples. Three barcoded samples were pooled at equimolar concentrations and multiplexed on single SMRT chips. Sequences were generated using the PacBio RS II system, which can achieve 99.5–99.9% sequence accuracy for DNA fragments using circular consensus technology (Travers et al., 2010), at the Genomics biotechnology company in Taiwan.

### Bioinformatics Analysis

The raw reads were de-multiplexed and primitively quality trimmed. After obtaining fastq files, we further processed the reads using MOTHUR (Schloss et al., 2009) to retain high-quality reads, i.e., reads that (1) were between 500 and 1000 nt long, (2) contained homopolymers of ≤15 nt, (3) lacked ambiguous bases, and (4) had average Phred scores of 20 or above. Potential chimeras were identified and discarded with UCHIME (Edgar et al., 2011) as implemented in USEARCH^1^, in reference mode with the UNITE (Kõljalg et al., 2013) reference dataset for UCHIME (v7.2) using the option –mindiv 3. On a per-sample basis, non-chimeric reads were clustered into operational taxonomic units (OTUs) using CD-HIT-EST (Fu et al., 2012) with a threshold of 97% pairwise similarity in accurate mode. Representative OTU sequences were searched against the UNITE+INSD database (v7.2) using VSEARCH (Rognes et al., 2016) global alignment to identify the taxonomy of the best hit. Any OTU without a hit or with only a weak hit (i.e., a hit for which the mean of the % sequence identity and % alignment coverage was <80%) was excluded. OTUs assigned to the same taxon were clustered together. Merging OTUs that match to the same reference sequence reduces the likelihood of erroneous diversity inflation due to poorly clustered sequences. The number of sequence reads per sample ranged from 3,047 to 13,463, so we subsampled and rarefied them to the smallest read number per sample (3,047). The raw sequence data sets are available in the European Nucleotide Archive under the study number PRJNA541480^2^. Potential fungal functional groups were identified using the online Guilds application tool: FUNGuildb (Nguyen et al., 2016). To compare the suitability and efficiency of long-read (full-length ITS) and short-read (ITS1 or ITS2) sequences for characterizing the deadwood mycobiomes of *C. carlesii*, we used the ITSx Perl-based software tool (Bengtsson-Palme et al., 2013) to extract ITS1 and ITS2 sequences from our full- length ITS sequences. Taxonomic assignment based on the ITS1 and ITS2 sequences was then performed as described previously using the UNITE+INSD database (v7.2) and VSEARCH (Rognes et al., 2016). We subsampled and rarefied the ITS1 and ITS2 sequences to the smallest read number per sample (2,773). Singletons were removed from the rarefied ITS1 and ITS2 datasets.

### Statistical Analysis

To assess the coverage/sequencing depth, individual rarefaction analyses were performed for each sample using the “diversity” function in PAST (Hammer et al., 2001). The sample-based rarefaction curves indicated non-saturation (albeit with sufficient sampling) of fungal diversity at the analyzed sequencing depth (3,047 reads), so we evaluated α-diversity using both the observed and chao1-estimated richness (both calculated using the “diversity” function in PAST). The community composition was analyzed in terms of both relative abundance (based on the Bray-Curtis similarity index) and presence/absence (based on the Jaccard similarity index) by using the “statistics” function to compute similarity and distance indices. A simplified phylogram generated by randomized axelerated maximum likelihood (RAxML) analysis based on complete ITS sequences was constructed to illustrate the phylogenetic placement of the 36 fungal OTUs that we identified at the species level. Phylogenetic trees were drawn using FigTree v. 1.4 (Rambaut, 2009). To compare the suitability and efficiency of long-read (full-length ITS) and short-read (ITS1 or ITS2) sequences, we repeated the above analyses using the ITS1 or ITS2 datasets (comparisons were performed using both the rarefied datasets with 2, 773 sequence reads per sample and the rarefied datasets with singleton removal). Comparisons of WIF OTU richness between the long-read (full-length ITS) and short-read (ITS1 or ITS2) datasets were performed using the paired *t*-test and the Jarque-Bera (JB) test for normality.

## Results

### Characteristics of the Sequence Datasets

A total of 24,096 good-quality filtered full-length fungal ITS sequences were obtained after removing non-target and chimeric sequences. After subsampling and rarefaction based on the smallest read number per sample (3,047), the sequences clustered into 284 fungal OTUs including 160 singletons, 34 doubletons, and 22 tripletons. The number of wood-inhabiting fungal (WIF) OTUs detected and the similarity of the community composition in *C. carlesii* deadwood depended on the choice of bioinformatics parameters (i.e., the use of rarefied or non-rarefied datasets and the inclusion or exclusion of rare OTUs) and statistical methods (Supplementary Figures S1A,B). Specifically, using only the 3,047 rarefied sequences for each sample reduced the number of detected OTUs by 36% (from 441 to 284; see Supplementary Figure S1A). However, rarefaction had little or no effect on the WIF community composition when evaluated in terms of relative abundance (using the Bray-Curtis similarity index) or presence/absence (using the Jaccard similarity index; see Supplementary Figure S1B). Removal of rare taxa strongly reduced the number of detected OTUs (by 48–71% for non-rarefied datasets and 56–76% for rarefied datasets; Supplementary Figure S1A). Removing rare OTUs markedly affected the community composition when evaluated in terms of presence/absence (using the Jaccard similarity index) but not when evaluated in terms of relative abundance. Removing singletons or singletons through tripletons increased the similarity of the samples (Supplementary Figure S1B). Subsequent analyses of the deadwood mycobiome are based exclusively on rarefied datasets with singletons removed. The impact of removing singletons was evaluated by performing a Mantel test based on the Jaccard distance measure (*R*_Mantel_ = 0.9996). On the basis of this test, 124 abundant fungal OTUs were retained for further analysis.

The lengths of the full ITS regions in the samples ranged from 500 to 780 bp (mode: 595–646 bp, mean: 617–635 bp). Information on the ITS sizes and distributions of representative sequences are shown in Supplementary Figure S2. In the final dataset, we were able to assign 83, 77, 73, 46, 55 and 29% of the total fungal OTUs at the phylum, class, order, family, genus, and species levels, respectively.

### Richness and Community Composition

*Castanopsis carlesii* deadwood was colonized by 85.33 ± 12.44 WIF OTUs (mean ± SE) on the basis of observed OTU richness (Table 1 and Supplementary Figure S3), or 313.43 ± 63.82 WIF OTUs based on Chao-1 estimated OTU richness. As shown in Supplementary Figure S1B, relative abundance data suggested that the WIF communities of the three deadwood samples were rather different (Bray-Curtis similarity index = 0.16–0.39%), but presence/absence data suggested the opposite (Jaccard similarity index = 0.41–0.61%).

**Table 1:** Observed wood-inhabiting fungal OTU richness and diversity of the three deadwood replicates used in this experiment with and without sequence normalization.

Sample	Non-normalized reads	Total OTU richness	Singleton	Chao1	Shannon H’
Replicate 1	3047	171	45	347	2.14
Replicate 2	13463	331	124	571	2.58
Replicate 3	7586	154	44	324	1.88
Total	24096	441			
**Sample**	**Normalized reads**	**Total OTU richness**	**Singleton**	**Chao1**	**Shannon H’**
Replicate 1	3047	171	69	347	2.15
Replicate 2	3047	153	60	403.3	2.56
Replicate 3	3047	92	31	190	1.86
Total	9141	284			

**FIGURE 1:**
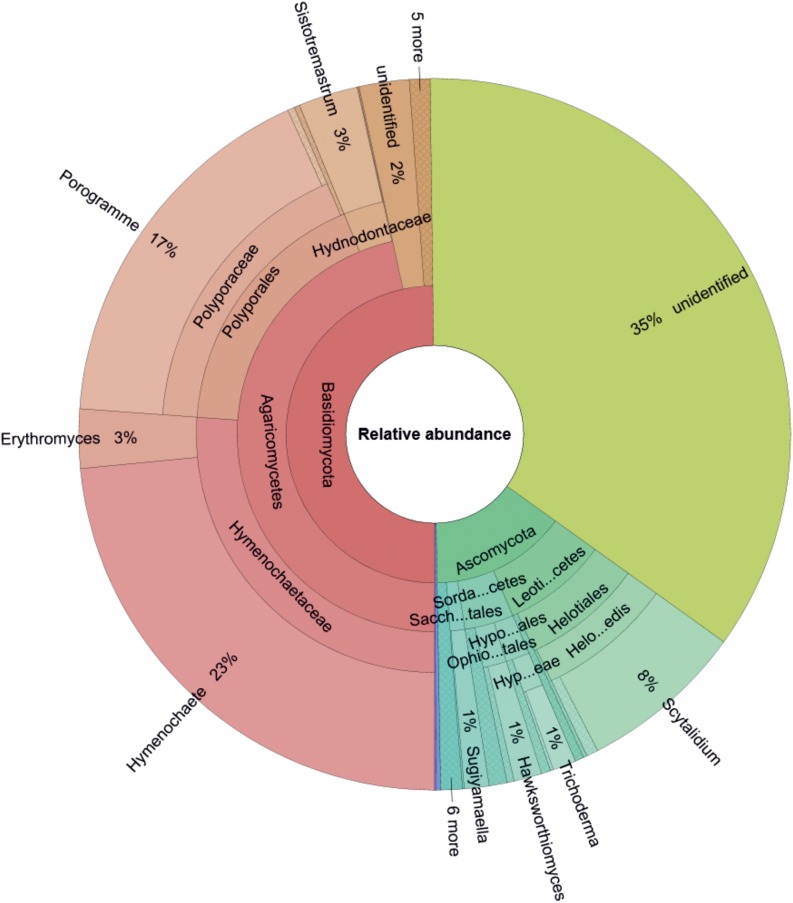
Genus-level composition of the wood-inhabiting fungal community associated with *Castanopsis carlesii* deadwood based on relative abundance data (considering only OTUs with relative abundances >1%) for three samples. Sacch…tales, Saccharomycetales; Sorda…cetes, Sordariomycetes; Leoti…cetes, Leotiomycetes; Ophio…tales, Ophiostomatales; Hypo…ales, Hypocreales; Hyp…eae, Hypocreaceae; Helo...edis, Helotiales_fam_Incertae_sedis.

Based on the relative abundance data (Figure 1), Basidiomycota were the most frequently detected phyla (50% of total sequences), followed by unidentified phyla (35% of total sequences), and Ascomycota (15% of total sequences). Mortierellomycota and Mucoromycota contributed very little (∼0.26% altogether). The most frequently detected Basidiomycota class was Agaricomycetes, which accounted for 47% of all sequences. This class was represented by the families Hymenochaetaceae (26% of total sequences, most of which were assigned to *Hymenochaete tongbiguanensis*), Polyporaceae (17% of total sequences, mostly assigned to *Porogramme albocincta*), and Hydnodontaceae (3% of total sequences, mostly assigned as *Sistotremastrum guttuliferum*). The most frequently detected Ascomycota classes were Leotiomycetes (9% of total sequences; represented by *Scytalidium* spp.) and Sordariomycetes (4% of total sequences; represented by *Hawksworthiomyces crousii*, *Trichoderma* spp., *Sporothrix* spp., *Xenoacremonium* spp., *Pestalotiopsis* spp.).

**FIGURE 2:**
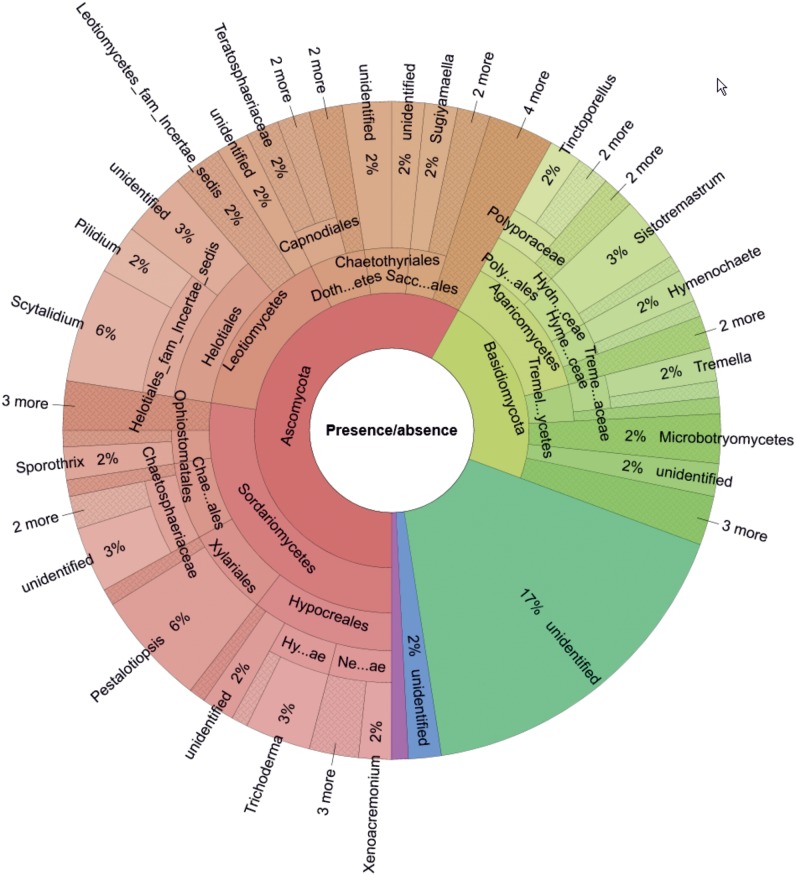
Genus-level composition of the wood-inhabiting fungal community associated with *Castanopsis carlesii* deadwood calculated based on presence/absence data for three samples. Tremel…ycetes, Tremellomycetes; Treme…aceae, Tremellaceae; Hyme…ceae, Hymenochaetaceae; Hydn…ceae, Hydnodontaceae; Poly…ales, Polyporales; Sacc…ales, Saccharomycetales; Doth…etes, Dothideomycetes; Chae…ales, Chaetosphaeriales; Hy…ae, Hypocreaceae; Ne…ae, Nectriaceae.

Based on presence/absence data (Figure 2), the most OTU-rich phylum was Ascomycota (58%, 72 OTUs), followed by Basidiomycota (23%, 28 OTUs), and unidentified phyla (17%, 21 OTUs). For Ascomycota, the most frequent classes were Sordariomycetes (27% of total detected OTUs; represented by *Trichoderma* spp. and *Pestalotiopsis* spp.) and Leotiomycetes (15% of total detected OTUs; represented by *Scytalidium* spp. and *Pilidium* spp.). The most frequently detected class of Basidiomycota was Agaricomycetes (13% of total detected OTUs; represented by *Sistotremastrum* spp., *Tinctoporellus* spp., and *Hymenochaete* spp.).

### Functional Groups of Wood-Inhabiting Fungi

**FIGURE 3:**
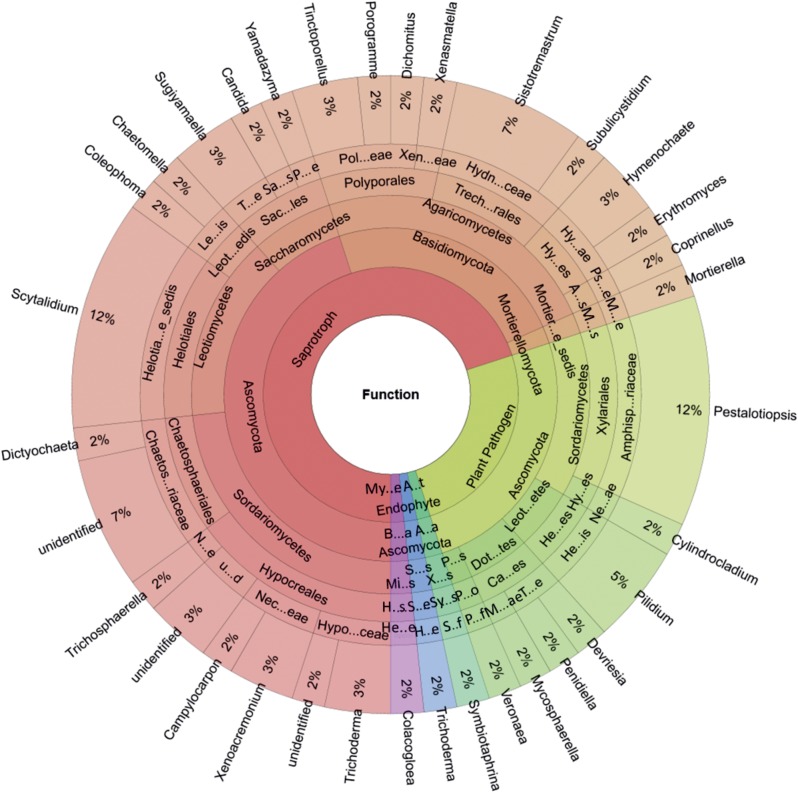
Genus-level proportions of different ecological functional groups of wood-inhabiting fungi associated with *Castanopsis carlesii* deadwood based on three samples. Hypo…ceae, Hypocreaceae; Nec…eae, Nectriaceae; u…d, unidentified; N…e, Niessliaceae; Chaetos…riaceae, Chaetosphaeriaceae; Helotia…e_sedis, Helotiales_fam_Incertae_sedis; Leot…edis, Leotiomycetes_ord_Incertae_sedis; Le…is, Leotiomycetes_fam_Incertae_sedis; Sac…les, Saccharomycetales; T…e, Trichomonascaceae; Sa…s, Saccharomycetales_fam_Incertae_sedis; P…e, Pichiaceae; Pol…eae, Polyporaceae; Xen…eae, Xenasmataceae; Trech…rales, Trechisporales; Hydn…ceae, Hydnodontaceae; Hy…es, Hymenochaetales; Hy…ae, Hymenochaetaceae; A…s, Agaricales; Ps…e, Psathyrellaceae; Mortier…e_sedis, Mortierellomycotina_cls_Incertae_sedis; M…s, Mortierellales; M…e, Mortierellaceae; Amphisp…riaceae, Amphisphaeriaceae; Hy…es, Hypocreales; Ne…ae, Nectriaceae; Leot…etes, Leotiomycetes; He…es, Helotiales; He…is, Helotiales_fam_Incertae_sedis; Dot…tes, Dothideomycetes; Ca…es, Capnodiales; T…e, Teratosphaeriaceae; M…ae, Mycosphaerellaceae; P…s, Pezizomycotina_cls_Incertae_sedis; P…o, Pezizomycotina_ord_Incertae_sedis; P…f, Pezizomycotina_fam_Incertae_sedis; A…t, Animal Endosymbiont; A…a, Ascomycota; X…s, Xylonomycetes, Sy…s, Symbiotaphrinales; S…f, Symbiotaphrinales_fam._Incertae_sedis; S…s, Sordariomycetes; Hy…s, Hypocreales; H…e, Hypocreaceae; My…e, Mycoparasite; B…a, Basidiomycota; Mi…s, Microbotryomycetes; H…s, Heterogastridiales, He…e, Heterogastridiaceae.

Five functional groups of fungi (60/124 OTUs) were identified using FUNGuild: animal endosymbionts, endophytes, mycoparasites, plant pathogens, and saprotrophs (Figure 3). The majority of the fungal OTUs were identified as saprotrophs (70% of successfully function-assigned OTUs, 42 OTUs), followed by plant pathogens (25% of successfully function-assigned OTUs, 15 OTUs). The saprotrophic community was dominated by Ascomycota [27 OTUs represented by *Trichoderma* spp. and *Xenoacremonium* spp. (Sordariomycetes), *Scytalidium* spp. (Leotiomycetes), and *Sugiyamaella* spp. (Saccharomycetes)] and Basidiomycota [14 OTUs represented by *Sistotremastrum* spp., *Tinctoporellus* spp., and *Hymenochaete* spp. (Agaricomycetes)]. The only detected plant pathogens (see Figure 3) belonged to the Ascomycota [15 OTUs represented by *Pestalotiopsis* spp. (Sordariomycetes) and *Pilidium* spp. (Leotiomycetes)].

### Phylogenetic Fungal Identification Based on Full Length ITS Sequences

**FIGURE 4:**
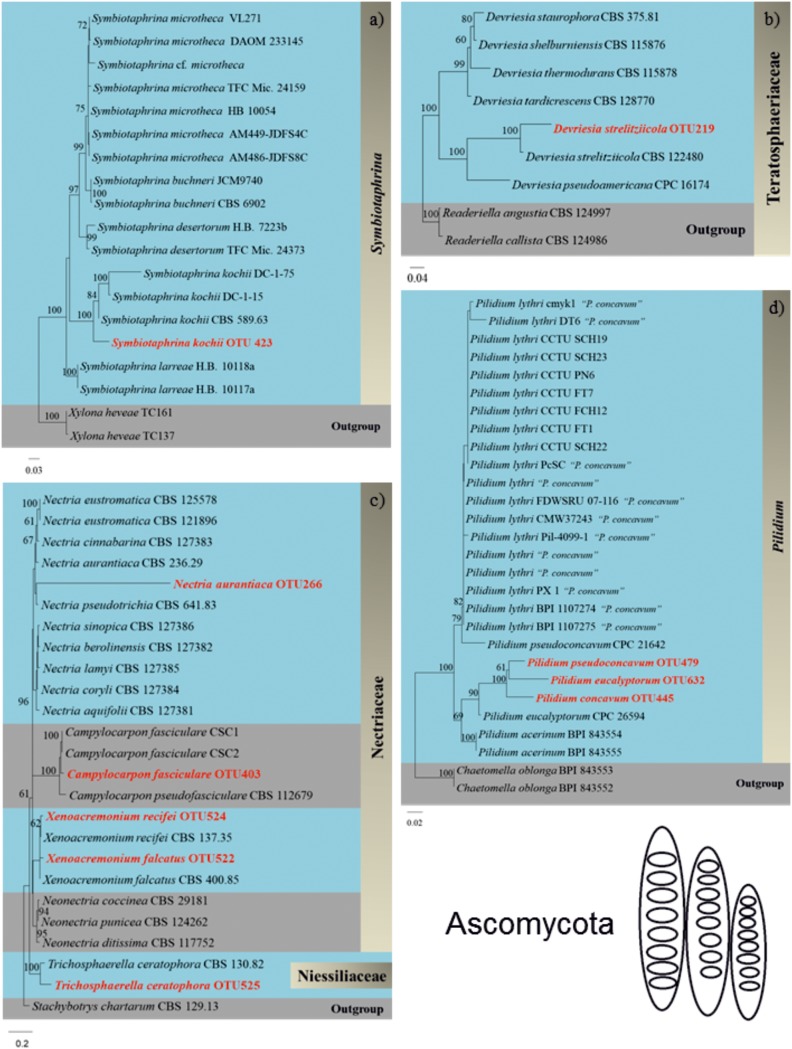
Phylograms (Ascomycota) of **(a)** *Symbiotaphrina kochii* (Xylonomycetes), **(b)** *Devriesia strelitziicola* (Dothideomycetes), **(c)** *Campylocarpon fasciculare*, *Xenoacremonium recifei*, *Xenoacremonium falcatus, Nectria aurantiaca*, and *Trichosphaerella ceratophora* (Sordariomycetes), and **(d)** *Pilidium concavum*, *Pilidium pseudoconcavum*, *Pilidium eucalyptorum* (Leotiomycetes) generated by randomized axelerated maximum likelihood analysis based on complete ITS sequences.

**FIGURE 5:**
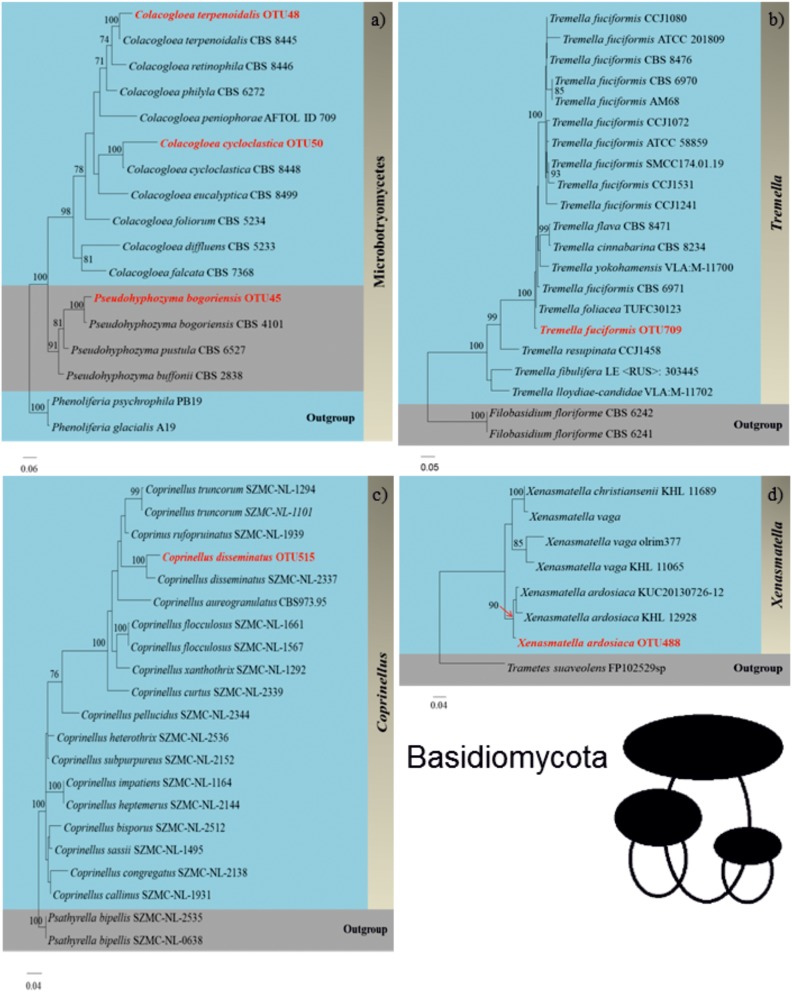
Phylograms (Basidiomycota) of **(a)** *Colacogloea terpenoidalis*, *Pseudohyphozyma bogoriensis*, and *Colacogloea cycloclastica* (Microbotryomycetes), **(b)** *Tremella fuciformis* (Tremellomycetes), **(c)** *Coprinellus disseminatus* (Agaricomycetes), and **(d)** *Xenasmatella ardosiaca* (Agaricomycetes) generated by randomized axelerated maximum likelihood analysis based on complete ITS sequences.

We successfully identified 36 WIF OTUs at the species level using the Pacbio full length ITS sequencing approach. A phylogram for these OTUs was generated manually from an RAxML analysis based on the full ITS sequences to illustrate the phylogeny of these WIF OTUs (Figure 4, 5 and Supplementary Figures S4–S15). We successfully confirmed the species identification of 14 OTUs with high bootstrap support (99–100%, Table 2), 6 OTUs with moderate bootstrap support (60–90%), and 4 with low bootstrap support (<60%). There were 12 OTUs for which the phylogram results differed from those obtained by Pacbio sequencing. For Ascomycota, the phylogram confirmed the species identification with high bootstrap support for 1/2 Dothideomycetes OTUs, 3/10 Sordariomycetes OTUs, 1/1 Xylonomycetes OTU, and no Leotiomycetes or Saccharomycetes OTUs. For Basidiomycota, the phylogram confirmed the species identifications for all Microbotryomycetes (3 OTUs), 6/10 Agaricomycetes OTUs, and no Tremellomycetes OTUs. There were only 4 OTUs whose taxonomic identity could not be assigned at the genus level (Table 2).

**Table 2:** Comparison of fungal taxonomic information on 36 wood-inhabiting fungi identified at the species level based on Pacbio sequencing alone and Pacbio sequencing with phylogenetic analysis based on randomized axelerated maximum likelihood analysis of the full internal transcribed spacer (ITS) sequences.

ID	Phylum	Class	Order	Family	Taxonomy based on Pacbio	Taxonomy based on Pacbio and phylogeny	Bootstrap (%)
DevriesiaOTU219	Ascomycota	Dothideomycetes	Capnodiales	Teratosphaeriaceae	*Devriesia strelitziicola*	***Devriesia strelitziicola***	100
AlternariaOTU741	Ascomycota	Dothideomycetes	Pleosporales	Pleosporaceae	*Alternaria alternata*	*Pleospora herbarum*	88
PilidiumOTU445	Ascomycota	Leotiomycetes	Helotiales	Incertae_sedis	*Pilidium concavum*	*Pilidium eucalyptorum*	90
PilidiumOTU479	Ascomycota	Leotiomycetes	Helotiales	Incertae_sedis	*Pilidium pseudoconcavum*	*Pilidium eucalyptorum*	90
PilidiumOTU632	Ascomycota	Leotiomycetes	Helotiales	Incertae_sedis	*Pilidium eucalyptorum*	*Pilidium eucalyptorum*	90
ScytalidiumOTU182	Ascomycota	Leotiomycetes	Helotiales	Incertae_sedis	*Scytalidium lignicola*	*Scytalidium lignicola*	<60
ScytalidiumOTU235	Ascomycota	Leotiomycetes	Helotiales	Incertae_sedis	*Scytalidium lignicola*	*Scytalidium lignicola*	<60
ScytalidiumOTU260	Ascomycota	Leotiomycetes	Helotiales	Incertae_sedis	*Scytalidium lignicola*	*Scytalidium lignicola*	<60
MeliniomycesOTU283	Ascomycota	Leotiomycetes	Incertae_sedis	Incertae_sedis	*Meliniomyces bicolor*	*Separate from existing Meliniomyces* sp.	100
SugiyamaellaOTU730	Ascomycota	Saccharomycetes	Saccharomycetales	Trichomonascaceae	*Sugiyamaella novakii*	*Sugiyamaella* spp.	77
CampylocarponOTU403	Ascomycota	Sordariomycetes	Hypocreales	Nectriaceae	*Campylocarpon fasciculare*	***Campylocarpon fasciculare***	100
TrichosphaerellaOTU525	Ascomycota	Sordariomycetes	Hypocreales	Niessliaceae	*Trichosphaerella ceratophora*	***Trichosphaerella ceratophora***	100
HawksworthiomycesOTU676	Ascomycota	Sordariomycetes	Ophiostomatales	Incertae_sedis	*Hawksworthiomyces crousii*	***Hawksworthiomyces crousii***	100
PestalotiopsisOTU124	Ascomycota	Sordariomycetes	Xylariales	Amphisphaeriaceae	*Pestalotiopsis maculiformans*	*Pestalotiopsis adusta*	87
TrichodermaOTU425	Ascomycota	Sordariomycetes	Hypocreales	Hypocreaceae	*Trichoderma harzianum*	*Trichoderma harzianum*	74
TrichodermaOTU432	Ascomycota	Sordariomycetes	Hypocreales	Hypocreaceae	*Trichoderma virens*	*Trichoderma harzianum*	74
XenoacremoniumOTU524	Ascomycota	Sordariomycetes	Hypocreales	Nectriaceae	*Xenoacremonium recifei*	*Xenoacremonium recifei*	62
DictyochaetaOTU98	Ascomycota	Sordariomycetes	Chaetosphaeriales	Chaetosphaeriaceae	*Dictyochaeta simplex*	*Dictyochaeta simplex*	60
NectriaOTU266	Ascomycota	Sordariomycetes	Hypocreales	Nectriaceae	*Nectria aurantiaca*	*Nectria pseudotrichia*	<60
XenoacremoniumOTU522	Ascomycota	Sordariomycetes	Hypocreales	Nectriaceae	*Xenoacremonium falcatus*	*Xenoacremonium falcatus*	<60
SymbiotaphrinaOTU423	Ascomycota	Xylonomycetes	Symbiotaphrinales	Incertae_sedis	*Symbiotaphrina kochii*	***Symbiotaphrina kochii***	100
CoprinellusOTU515	Basidiomycota	Agaricomycetes	Agaricales	Psathyrellaceae	*Coprinellus disseminatus*	***Coprinellus disseminatus***	100
ErythromycesOTU623	Basidiomycota	Agaricomycetes	Hymenochaetales	Hymenochaetaceae	*Erythromyces crocicreas*	***Erythromyces crocicreas***	100
HymenochaeteOTU500	Basidiomycota	Agaricomycetes	Hymenochaetales	Hymenochaetaceae	*Hymenochaete tongbiguanensis*	***Hymenochaete tongbiguanensis***	100
SubulicystidiumOTU541	Basidiomycota	Agaricomycetes	Trechisporales	Hydnodontaceae	*Subulicystidium perlongisporum*	***Subulicystidium perlongisporum***	100
SistotremastrumOTU194	Basidiomycota	Agaricomycetes	Trechisporales	Hydnodontaceae	*Sistotremastrum guttuliferum*	***Sistotremastrum guttuliferum***	99
SistotremastrumOTU493	Basidiomycota	Agaricomycetes	Trechisporales	Hydnodontaceae	*Sistotremastrum guttuliferum*	***Sistotremastrum guttuliferum***	99
XenasmatellaOTU488	Basidiomycota	Agaricomycetes	Polyporales	Xenasmataceae	*Xenasmatella ardosiaca*	*Xenasmatella ardosiaca*	90
DichomitusOTU309	Basidiomycota	Agaricomycetes	Polyporales	Polyporaceae	*Dichomitus squalens*	*Tinctoporellus* sp. and *Porogramme* sp. clade	74
PorogrammeOTU377	Basidiomycota	Agaricomycetes	Polyporales	Polyporaceae	*Porogramme albocincta*	*Tinctoporellus* sp. and *Porogramme* sp. clade	74
TinctoporellusOTU512	Basidiomycota	Agaricomycetes	Polyporales	Polyporaceae	*Tinctoporellus epimiltinus*	*Tinctoporellus* sp. and *Porogramme* sp. clade	74
JianyuniaOTU49	Basidiomycota	Agaricostilbomycetes	Agaricostilbales	Incertae_sedis	*Jianyunia sakaguchii*	*Jianyunia sakaguchii*	78
ColacogloeaOTU48	Basidiomycota	Microbotryomycetes	Heterogastridiales	Heterogastridiaceae	*Colacogloea terpenoidalis*	***Colacogloea terpenoidalis***	100
ColacogloeaOTU50	Basidiomycota	Microbotryomycetes	Sporidiobolales	Incertae_sedis	*Colacogloea cycloclastica*	***Colacogloea cycloclastica***	100
PseudohyphozymaOTU45	Basidiomycota	Microbotryomycetes	Incertae_sedis	Incertae_sedis	*Pseudohyphozyma bogoriensis*	***Pseudohyphozyma bogoriensis***	100
TremellaOTU709	Basidiomycota	Tremellomycetes	Tremellales	Tremellaceae	*Tremella fuciformis*	*Tremella* spp.	100

*Green backgrounds indicate identical identification at the species level (bold letters indicate 99 – 100% Bootstrap support). Orange backgrounds indicate identical identification at the genus level. Red backgrounds indicate different identification at the genus level.*

### Community Composition, Richness, and Taxonomic Identification of the *Castanopsis carlesii* Deadwood Mycobiome Based on the ITS1, ITS2, and Full-Length ITS Sequences

Analyses of fungal community composition based on the ITS1 or ITS2 sequences yielded similar results to the analysis based on the full-length ITS sequences (Supplementary Figure S16, Supplementary Appendix 1). Although the numbers of sequence reads in the rarefied ITS1 and ITS2 datasets were lower than that for the full-length ITS dataset (2,773 for ITS1 and ITS2 vs. 3,047 for full-length ITS), the richness determined in the ITS2-based analysis was significantly greater than that determined based on the full ITS sequences (*P* = 0.003, 26%) and marginally higher than that for ITS1 (*P* = 0.058, 17%). The richness obtained by analyzing the full-length ITS sequences did not differ significantly from that for the ITS1 dataset (*P* = 0.179), but the ITS1 richness values were non-significantly higher (by 8% on average) for all three samples. Singleton removal did not change the finding that shorter reads (especially ITS2) yield higher richness than full-length ITS sequences. The number of OTUs identified at the species level from the rarified datasets was highest for the analysis using ITS1 (46) followed by ITS2 (41) and the full-length ITS (36) (Supplementary Table S1, Supplementary Appendix 1). However, we found many cases where sequences from a single OTU based on full-length ITS data were spliced into multiple OTUs when ITS1 or ITS2 data were used with 97% cutoff. For example, we detected one OTU of *Trichoderma harzianum* using the full-length ITS data but 12 and 9 different OTUs identified as *T. harzianum* were detected using the ITS1 and ITS2 data, respectively. Using the rarefied ITS1 and ITS2 datasets, we repeated the construction of phylogenetic trees for the 36 WIF OTUs previously identified at species level based on full-length ITS data. The taxonomic identifications based on full-length ITS, ITS1, and ITS2 data were not in full agreement: 17/36 OTUs (47%) identified at species level using full length ITS data were also detected using the ITS1 or ITS2 data, but 12/36 OTUs (33%) disappeared completely (Supplementary Table S1, Supplementary Appendix 1). We also attempted to identify these OTUs using a phylogenetic approach based on the ITS1 or ITS2 data. The phylogenetic trees based on ITS2 sequences yielded much better results than those based on ITS1; we successfully used the ITS2 data to confirm the species identification of 8 OTUs with high bootstrap support (99–100%, Supplementary Table S2, Supplementary Appendix 1). Nevertheless, the bootstrap values obtained in most cases when using the ITS1 and ITS2 datasets were too low to permit reliable species-level identification. Comparisons of the phylogenetic trees based on the full-length ITS, ITS1, and ITS2 sequences are presented in Supplementary Figures S17–S36 and summarized in Supplementary Table S2, Supplementary Appendix 1.

## Discussion

We successfully characterized a deadwood mycobiome by Pacbio sequencing of full-length ITS sequences. Despite the possible biases inherent to molecular techniques (Tedersoo et al., 2010; Voříšková and Baldrian, 2013), our work provides important and novel insights into the distribution and diversity of wood-inhabiting fungi in a tropical forest.

### Bioinformatics Parameter Choices Affected OTU Richness and Community Composition: The Strong Effect of Removing Rare Taxa

Rarefaction to equalize the numbers of sequences from different samples and removal of rare taxa (i.e., singletons, doubletons, and tripletons, etc.) are considered to be important workflow elements for bioinformatics studies involving NGS amplicon sequencing (Lindahl et al., 2013). Rarefaction is essential for accurate estimation of species richness and diversity, but these is not yet a consensus regarding its impact on assessments of community composition (McMurdie and Holmes, 2014). Our results confirm the strong effect of rarefaction on OTU richness: the results obtained with rarefaction (in which case the ranking of the replicates in terms of richness was replicate1 > replicate2 > replicate3) differed from those without (replicate2 > replicate1 > replicate3). The effect of rarefaction on the calculated fungal community composition was less pronounced than that for OTU richness, and depended on the type of data under consideration (relative abundance or presence/absence) and the choice of similarity measure (i.e., Bray-Curtis or Jaccard). Rare OTUs identified in NGS datasets may be genuine rare OTUs or artifacts originating from sequencing errors (Lindahl et al., 2013; Dissanayake et al., 2018; Jayawardena et al., 2018). Genuine rare OTUs can be distinguished by combining NGS and culture-dependent techniques (using multigene phylogenetic approaches), but this is time-consuming and requires experience (Dissanayake et al., 2018; Jayawardena et al., 2018). In general, singleton removal is recommended as a minimal measure to eliminate sequencing artifacts (Lindahl et al., 2013) but doubletons and tripletons are commonly removed as well for this purpose (Hoppe et al., 2016; Purahong et al., 2016, 2017). Removing singletons alone approximately halved the OTU richness for both the rarefied and un-rarefied datasets, while removing singletons through tripletons reduced OTU richness by over 70%. Removing rare OTUs had a negligible effect on the fungal community composition based on relative abundance data (and the Bray-Curtis similarity index), but strongly affected the composition based on presence/absence data (and the Jaccard similarity index). It should be noted that because of the high variability of ITS copy numbers and primer biases, relative abundance data for mycobiomes obtained by next generation sequencing may be unreliable (Amend et al., 2010; Lindahl et al., 2013). In addition, when using fungal universal primers, the relative read abundance of individual species will always be affected by other species in the pool because reagents (e.g., PCR primers or DNA capture beads) will be limiting in studies on high diversity environments, causing taxa with high template abundance or high PCR affinity to reduce the read abundance of other taxa (Amend et al., 2010). Therefore, presence/absence data may be more useful for assessing fungal community composition in NGS-based studies (Purahong et al., 2018a). When using presence/absence data, we recommend rarefaction to equalize read numbers between samples and removing at least singleton OTUs.

### The Taxonomic and Functional Diversity of the *Castanopsis carlesii* Deadwood Mycobiome

The WIF OTU richness of *C. carlesii* deadwood in the tropical rainforest (53 ± 3 OTUs, mean ± SE) greatly exceeded that determined for other Fagaceae deadwood in temperate forests (*Fagus sylvatica* 22 ± 2 OTUs, mean ± SE; *Quercus* spp. 34 ± 3 OTUs, mean ± SE) using a similar bioinformatics strategy and data processing workflow (∼3000 sequence reads per sample and removal of singletons through quadrupletons) (Purahong et al., 2018b). The mycobiome of early decay *C. carlesii* deadwood was diverse but dominated by Ascomycota and Basidiomycota. Similar patterns were observed in both temperate and subtropical forests (Hoppe et al., 2016; Purahong et al., 2017, 2018b). When mycobiome composition was analyzed on the basis of relative abundance data, Basidiomycota was the most frequent fungal phylum, but Ascomycota was more frequent when composition was analyzed in terms of presence/absence. However, the composition of the *C. carlesii* deadwood mycobiome in the rainforest differed markedly from that of other early decay Fagaceae deadwood in temperate forests when family- and genus- level data were considered. For example, the most frequent fungal families detected in *F. sylvatica* deadwood were Xylariaceae and Hypoxylaceae (represented by *Annulohypoxylon*, *Hypoxylon*, and *Xylaria*), while those for *Quercus* spp. deadwood were Physalacriaceae and Stereaceae (represented by *Armillaria* and *Stereum*). These families were rare in *C. carlesii* deadwood (represented by Hymenochaetaceae and Polyporaceae: *Hymenochaete* and *Porogramme*).

Diverse functional groups of fungi were detected in the deadwood, including animal endosymbionts, endophytes, mycoparasites, plant pathogens, and saprotrophs. Saprotrophs and plant pathogens were the dominant functional groups, accounting for 95% of all successfully function-assigned OTUs. Similar extreme dominance of saprotrophs and plant pathogens has been observed in broadleaved and conifer deadwood in temperate and subtropical forests. The frequent detection of plant fungal pathogens (*Pestalotiopsis* spp. and *Pilidium* spp.) in deadwood in this ecosystem and in temperate and subtropical forests suggests that deadwood may generally serve as an inoculum source of plant pathogenic fungi (Purahong et al., 2017, 2018a). In fact, *Pestalotiopsis* spp. were recently reported to persist in dead leaves and wood as saprobes, suggesting that they may be able to switch between plant pathogen and saprotrophic lifestyles (Maharachchikumbura et al., 2014).

### Is Pacbio Sequencing Suitable for Studying the Mycobiomes of Moderately Complex Fungal Communities?

An earlier study using Pacbio sequencing to characterize fungal communities in environmental samples concluded that that this method can be used to characterize mycobiomes of relatively low diversity (Tedersoo et al., 2018). Our results extend this finding by showing that high sequencing depths (>10,000 sequence reads per sample) can be achieved with Pacbio. The cost of Pacbio sequencing has dropped dramatically, suggesting that it may become viable for the analysis of high diversity mycobiomes in the near future. For moderately complex deadwood fungal communities, Pacbio offers comparable performance to 454 pyrosequencing but provides full-length ITS sequences (Hoppe et al., 2016; Purahong et al., 2017, 2018b).

### Confirming Species- and Genus-Level Taxonomic Assignments by Phylogenetic Analysis Based on Full-Length ITS Sequences Obtained by Pacbio Sequencing

The ITS region varies widely between fungal species (i.e., it displays high interspecific variation) but exhibits only modest intraspecific variation (Nilsson et al., 2009; Lindahl et al., 2013). If its variation is too high, it can be difficult to derive phylogenies at the family and order levels (Lindahl et al., 2013). However, high ITS variability is useful for genus and species identification, which is why the ITS region was chosen as the “official” fungal barcode sequence (Schoch et al., 2012). In particular, it was concluded to be superior to the LSU and SSU for identifying species belonging to the most important classes of Ascomycota (Eurotiomycetes, Sordariomycetes, and Dothideomycetes) (Hongsanan et al., 2018). In this work, a phylogenetic analysis based on full-length ITS reads obtained by Pacbio sequencing was used to confirm species-level taxonomic assignments (with 100% bootstrap support) for some members of the Sordariomycetes and Dothideomycetes. For Basidiomycota, the phylogenetic analysis confirmed the species-level identifications of some members of the Agaricomycetes and all members of the Microbotryomycetes based on Pacbio sequencing. In addition, the genus-level assignments based on the phylogenetic analysis were generally in agreement with those based on the full length ITS data obtained by Pacbio sequencing. Although this work includes a clear phylogenetic analysis based on full-length ITS data obtained by Pacbio sequencing, this approach cannot be used to confirm species-level taxonomic assignments for all fungi. For example, we could not achieve species assignment for any members of Leotiomycetes (Ascomycota) with high bootstrap support. Furthermore, it should be noted that general and reliable species identification requires multigene phylogenetic analysis based on 5 genes: the ITS, large subunit nuclear rRNA (LSU), small subunit nuclear rRNA (SSU), RNA polymerase second largest subunit (RPB2), and the translation elongation factor 1-alpha gene (TEF) (Mapook et al., 2016; Dissanayake et al., 2018; Jayawardena et al., 2018).

### Comparing Short-Read (ITS1 and ITS2) and Long-Read (Full-Length ITS) Sequences: What Can We Learn?

The use of short-read sequences caused species richness to be overestimated by between 8% (ITS1) and 26% (ITS2). In several cases, sequences assigned to a single OTU based on full-length ITS data were spliced into multiple OTUs when ITS1 or ITS2 data were used with a 97% cutoff. This partly explains the high OTU richness observed in analyses based on short-read ITS1 or ITS2 sequences. Furthermore, the short-read ITS1 and ITS2 sequences were not especially useful for phylogenetic identification, so similarity-based methods are more suitable for taxonomic assignment based on these short-read sequences. However, long-read sequencing of the full-length ITS may suffer from length bias (Ihrmark et al., 2012; Kennedy et al., 2018; Tedersoo et al., 2018) – for example, biases against the long ITS sequences of some taxonomic fungal groups such as Chantarellaceae and the genus *Leccinum* (which has long inserts). These species will be underrepresented in datasets based on the full ITS region. Length bias could potentially have very important effects on the interpretation of long-read NGS data, but few studies have explored this issue (Tedersoo et al., 2018). To evaluate this effect, it will be necessary to analyze a mock community with a known proportion of diverse fungal species with long ITS regions.

## Conclusion and Outlook

This work presents the first attempt to characterize a deadwood mycobiome using Pacbio sequencing of the full-length fungal nuclear ribosomal ITS region. We successfully obtained full length ITS sequences (which were less than 1 kb long) and showed that the deadwood mycobiome of *C. carlesii* in tropical forests is taxonomically and functionally diverse. Our results suggest that it should be possible to routinely achieve full length sequencing of other fungal genetic markers such as RPB2, TEF, LSU, and SSU, all of which are typically below 1.5 kb in length. These sequences are important for fungal identification using multigene phylogeny. In the future, when sufficient data on these and other genes have been deposited in public databases, it may become possible to routinely use multigene phylogeny to correctly identify fungal species from environmental samples. Unfortunately, the phylogeny-based identification procedure used in this work can only be applied to fungal classes for which species identification can be achieved on the basis of full length ITS sequences.

## Ethics Statement

A field work permit was issued by the National Pingtung University of Science and Technology, Pingtung, Taiwan.

## Author Contributions

Y-TW, C-TC, and WP conceived and designed the experiments. Y-TW performed the field and laboratory experiments. WP and Y-TW performed the bioinformatics and analyzed the data. AM performed phylogenetic analysis. Y-TW contributed to reagents, materials, and analysis tools. WP and Y-TW wrote the manuscript. Y-TW, AM, and C-TC commented and revised the manuscript. All authors reviewed and approved the manuscript before its submission.

## Conflict of Interest Statement

The authors declare that the research was conducted in the absence of any commercial or financial relationships that could be construed as a potential conflict of interest.

## Footnotes

1 http://www.drive5.com/usearch

2 https://www.ncbi.nlm.nih.gov/bioproject/PRJNA541480?fbclid=IwAR2VJM55YqStRYnHYjsCo4ai4FZplkXcJ1pGvXYLfLRbs_cYiwHC-GVMi8w

## References

[B1] Amend A. S. Seifert K. A. Bruns T. D. (2010). Quantifying microbial communities with 454 pyrosequencing: does read abundance count? *Mol. Ecol.* 19 5555–5565. 10.1111/j.1365-294X.2010.04898.x 21050295

[B2] Bengtsson-Palme J. Ryberg M. Hartmann M. Branco S. Wang Z. Godhe A. (2013). Improved software detection and extraction of ITS1 and ITS2 from ribosomal ITS sequences of fungi and other eukaryotes for analysis of environmental sequencing data. *Methods Ecol. Evol.* 4 914–919. 10.1111/2041-210X.12073

[B3] Dissanayake A. J. Purahong W. Wubet T. Hyde K. D. Wei Z. Xu H. (2018). Direct comparison of culture-dependent and culture-independent molecular approaches reveal the diversity of fungal endophytic communities in stems of grapevine (*Vitis vinifera*). *Fungal Divers.* 90 85–107. 10.1007/s13225-018-0399-3

[B4] Edgar R. C. Haas B. J. Clemente J. C. Quince C. Knight R. (2011). UCHIME improves sensitivity and speed of chimera detection. *Bioinformatics* 27 2194–2200. 10.1093/bioinformatics/btr381 21700674 PMC3150044

[B5] Fu L. Niu B. Zhu Z. Wu S. Li W. (2012). CD-HIT: accelerated for clustering the next-generation sequencing data. *Bioinformatics* 28 3150–3152. 10.1093/bioinformatics/bts565 23060610 PMC3516142

[B6] Gao C. Shi N.-N. Liu Y.-X. Peay K. G. Zheng Y. Ding Q. (2013). Host plant genus-level diversity is the best predictor of ectomycorrhizal fungal diversity in a Chinese subtropical forest. *Mol. Ecol.* 22 3403–3414. 10.1111/mec.12297 24624421

[B7] Goldmann K. Schöning I. Buscot F. Wubet T. (2015). Forest management type influences diversity and community composition of soil fungi across temperate forest ecosystems. *Front. Microbiol.* 6:1300. 10.3389/fmicb.2015.01300 26635766 PMC4656839

[B8] Goodwin S. McPherson J. D. McCombie W. R. (2016). Coming of age: ten years of next-generation sequencing technologies. *Nat. Rev. Genet.* 17 333–351. 10.1038/nrg.2016.49 27184599 PMC10373632

[B9] Hammer Ø. Harper D. A. T. Ryan P. D. (2001). Past: paleontological statistics software package for education and data analysis. *Palaeontol. Electron.* 4:9.

[B10] Hodge S. J. Peterken G. F. (1998). Deadwood in british forests: priorities and a strategy. *Forestry* 71 99–112. 10.1093/forestry/71.2.99

[B11] Hongsanan S. Jeewon R. Purahong W. Xie N. Liu J.-K. Jayawardena R. S. (2018). Can we use environmental DNA as holotypes? *Fungal Divers.* 92 1–30. 10.1007/s13225-018-0404-x

[B12] Hoppe B. Purahong W. Wubet T. Kahl T. Bauhus J. Arnstadt T. (2016). Linking molecular deadwood-inhabiting fungal diversity and community dynamics to ecosystem functions and processes in central European forests. *Fungal Divers.* 77 367–379. 10.1007/s13225-015-0341-x

[B13] Ihrmark K. Bödeker I. T. M. Cruz-Martinez K. Friberg H. Kubartova A. Schenck J. (2012). New primers to amplify the fungal ITS2 region – evaluation by 454-sequencing of artificial and natural communities. *FEMS Microbiol. Ecol.* 82 666–677. 10.1111/j.1574-6941.2012.01437.x 22738186

[B14] Jayawardena R. Purahong W. Zhang W. Wubet T. Li X. Liu M. (2018). Biodiversity of fungi on *Vitis vinifera* L. revealed by traditional and high-resolution culture-independent approaches. *Fungal Divers* 90 1–84. 10.1007/s13225-018-0398-4

[B15] Kennedy P. G. Cline L. C. Song Z. (2018). Probing promise versus performance in longer read fungal metabarcoding. *New Phytol.* 217 973–976. 10.1111/nph.14883 29334600

[B16] Kõljalg U. Nilsson R. H. Abarenkov K. Tedersoo L. Taylor A. F. S. Bahram M. (2013). Towards a unified paradigm for sequence-based identification of fungi. *Mol. Ecol.* 22 5271–5277. 10.1111/mec.12481 24112409

[B17] Lindahl B. D. Nilsson R. H. Tedersoo L. Abarenkov K. Carlsen T. Kjøller R. (2013). Fungal community analysis by high-throughput sequencing of amplified markers – a user’s guide. *New Phytol.* 199 288–299. 10.1111/nph.12243 23534863 PMC3712477

[B18] Maharachchikumbura S. S. N. Hyde K. D. Groenewald J. Z. Xu J. Crous P. W. (2014). Pestalotiopsis revisited. *Stud. Mycol.* 79 121–186. 10.1016/j.simyco.2014.09.005 25492988 PMC4255583

[B19] Mapook A. Boonmee S. Ariyawansa H. A. Tibpromma S. Campesori E. Jones E. B. G. (2016). Taxonomic and phylogenetic placement of nodulosphaeria. *Mycol. Prog.* 15:34 10.1007/s11557-016-1176-x

[B20] McMurdie P. J. Holmes S. (2014). Waste not, want not: why rarefying microbiome data is inadmissible. *PLoS Comput. Biol.* 10:e1003531. 10.1371/journal.pcbi.1003531 24699258 PMC3974642

[B21] Nawaz A. Purahong W. Lehmann R. Herrmann M. Küsel K. Totsche K. U. (2016). Superimposed pristine limestone aquifers with marked hydrochemical differences exhibit distinct fungal communities. *Front. Microbiol.* 7:666. 10.3389/fmicb.2016.00666 27242696 PMC4860458

[B22] Nguyen N. H. Song Z. Bates S. T. Branco S. Tedersoo L. Menke J. (2016). FUNGuild: an open annotation tool for parsing fungal community datasets by ecological guild. *Fungal Ecol.* 20 241–248. 10.1016/j.funeco.2015.06.006

[B23] Nilsson R. H. Ryberg M. Abarenkov K. Sjökvist E. Kristiansson E. (2009). The ITS region as a target for characterization of fungal communities using emerging sequencing technologies. *FEMS Microbiol. Lett.* 296 97–101. 10.1111/j.1574-6968.2009.01618.x 19459974

[B24] Purahong W. Pietsch K. A. Lentendu G. Schöps R. Bruelheide H. Wirth C. (2017). Characterization of unexplored deadwood mycobiome in highly diverse subtropical forests using culture-independent molecular technique. *Front. Microbiol.* 8:574. 10.3389/fmicb.2017.00574 28469600 PMC5395659

[B25] Purahong W. Wubet T. Kahl T. Arnstadt T. Hoppe B. Lentendu G. (2018a). Increasing N deposition impacts neither diversity nor functions of deadwood-inhabiting fungal communities, but adaptation and functional redundancy ensure ecosystem function. *Environ. Microbiol.* 20 1693–1710. 10.1111/1462-2920.14081 29473288

[B26] Purahong W. Wubet T. Krüger D. Buscot F. (2018b). Molecular evidence strongly supports deadwood-inhabiting fungi exhibiting unexpected tree species preferences in temperate forests. *ISME J.* 12 289–295. 10.1038/ismej.2017.177 29087376 PMC5739023

[B27] Purahong W. Wubet T. Krüger D. Buscot F. (2018c). Application of next-generation sequencing technologies to conservation of wood-inhabiting fungi. *Conserv. Biol.* 10.1111/cobi.13240 [Epub ahead of print]. 30350883

[B28] Purahong W. Wubet T. Lentendu G. Schloter M. Pecyna M. J. Kapturska D. (2016). Life in leaf litter: novel insights into community dynamics of bacteria and fungi during litter decomposition. *Mol. Ecol.* 25 4059–4074. 10.1111/mec.13739 27357176

[B29] Quail M. A. Smith M. Coupland P. Otto T. D. Harris S. R. Connor T. R. (2012). A tale of three next generation sequencing platforms: comparison of ion torrent, pacific biosciences and illumina miseq sequencers. *BMC Genomics* 13:341. 10.1186/1471-2164-13-341 22827831 PMC3431227

[B30] Rajala T. Peltoniemi M. Pennanen T. Mäkipää R. (2010). Relationship between wood-inhabiting fungi determined by molecular analysis (denaturing gradient gel electrophoresis) and quality of decaying logs. *Can. J. For. Res.* 40 2384–2397. 10.1139/X10-176

[B31] Rajala T. Peltoniemi M. Pennanen T. Mäkipää R. (2012). Fungal community dynamics in relation to substrate quality of decaying norway spruce (*Picea abies* [L.] Karst) logs in boreal forests. *FEMS Microbiol. Ecol.* 81 494–505. 10.1111/j.1574-6941.2012.01376.x 22458543

[B32] Rambaut A. (2009). *FigTree v 1.4: Tree Figure Drawing Tool.* Available at: http://tree.bio.ed.ac.uk/software/figtree/

[B33] Rhoads A. Au K. F. (2015). PacBio sequencing and its applications. *Genomics Proteomics Bioinformatics* 13 278–289. 10.1016/j.gpb.2015.08.002 26542840 PMC4678779

[B34] Rognes T. Flouri T. Nichols B. Quince C. Mahé F. (2016). VSEARCH: a versatile open source tool for metagenomics. *PeerJ* 4:e2584. 10.7717/peerj.2584 27781170 PMC5075697

[B35] Schloss P. D. Westcott S. L. Ryabin T. Hall J. R. Hartmann M. Hollister E. B. (2009). Introducing mothur: open-source, platform-independent, community-supported software for describing and comparing microbial communities. *Appl. Environ. Microbiol.* 75 7537–7541. 10.1128/AEM.01541-09 19801464 PMC2786419

[B36] Schoch C. L. Seifert K. A. Huhndorf S. Robert V. Spouge J. L. Levesque C. A. (2012). Nuclear ribosomal internal transcribed spacer (ITS) region as a universal DNA barcode marker for fungi. *Proc. Natl. Acad. Sci. U.S.A.* 109 6241–6246. 10.1073/pnas.1117018109 22454494 PMC3341068

[B37] Stokland J. N. Siitonen J. Jonsson B. G. (2012). *Biodiversity in Dead Wood.* New York, NY: Cambridge University Press.

[B38] Tedersoo L. Nilsson R. H. Abarenkov K. Jairus T. Sadam A. Saar I. (2010). 454 Pyrosequencing and Sanger sequencing of tropical mycorrhizal fungi provide similar results but reveal substantial methodological biases. *New Phytol.* 188 291–301. 10.1111/j.1469-8137.2010.03373.x 20636324

[B39] Tedersoo L. Tooming-Klunderud A. Anslan S. (2018). PacBio metabarcoding of Fungi and other eukaryotes: errors, biases and perspectives. *New Phytol.* 217 1370–1385. 10.1111/nph.14776 28906012

[B40] Travers K. J. Chin C.-S. Rank D. R. Eid J. S. Turner S. W. (2010). A flexible and efficient template format for circular consensus sequencing and SNP detection. *Nucleic Acids Res.* 38:e159. 10.1093/nar/gkq543 20571086 PMC2926623

[B41] Vályi K. Rillig M. C. Hempel S. (2015). Land-use intensity and host plant identity interactively shape communities of arbuscular mycorrhizal fungi in roots of grassland plants. *New Phytol.* 205 1577–1586. 10.1111/nph.13236 25545193

[B42] Voříšková J. Baldrian P. (2013). Fungal community on decomposing leaf litter undergoes rapid successional changes. *ISME J.* 7 477–486. 10.1038/ismej.2012.116 23051693 PMC3578564

[B43] Vu D. Groenewald M. de Vries M. Gehrmann T. Stielow B. Eberhardt U. (2019). Large-scale generation and analysis of filamentous fungal DNA barcodes boosts coverage for kingdom fungi and reveals thresholds for fungal species and higher taxon delimitation. *Stud. Mycol.* 92 135–154. 10.1016/j.simyco.2018.05.001 29955203 PMC6020082

[B44] Walder F. Schlaeppi K. Wittwer R. Held A. Y. Vogelgsang S. Heijden V. D. (2017). Community profiling of fusarium in combination with other plant-associated fungi in different crop species using smrt sequencing. *Front. Plant Sci.* 8:2019. 10.3389/fpls.2017.02019 29234337 PMC5712420

[B45] Yang S. Z. Chang S. C. Lin Y. J. Li C. F. (2015). Vegetation classification of lower montane secondary forest in southeastern Taiwan. *Quarterly Journal of Chinese Forestry.*

