# Supplementary material for: Characterization of the Castanopsis carlesii Deadwood Mycobiome by Pacbio Sequencing of the Full-Length Fungal Nuclear Ribosomal Internal Transcribed Spacer (ITS)
Source: Front Microbiol. 2019 May 17;10:983. doi: 10.3389/fmicb.2019.00983 (PMC6540943; doi:10.3389/fmicb.2019.00983)

Supplementary materials

Characterization of the *Castanopsis carlesii* deadwood mycobiome by Pacbio sequencing of the full-length fungal nuclear ribosomal internal transcribed spacer (ITS)

Author names:

Witoon Purahong[1], Ausana Mapook[1, 2], Yu-Ting Wu[3], Chaur-Tzuhn Chen[3]

Affiliations:

[1]Department of Soil Ecology, UFZ-Helmholtz Centre for Environmental Research, Theodor-Lieser-Str. 4, D-06120 Halle (Saale), Germany; [2]Center of Excellence in Fungal Research, Mae Fah Luang University, Chiang Rai 57100, Thailand , [3]Department of Forestry, National Pingtung University of Science and Technology, Pingtung, Taiwan

Figure S1. Information on detected observed OTU richness (a) and similarity (b) of community composition in *Castanopsis carlesii* deadwood mycobiomes subjected to different bioinformatics parameters (rarified and non-rarified datasets/and rare OTUs included/removed).

a

Parameter	Detected OTU richness
All	441
Singletons removed	228
Singletons to tripletons removed	127
Rarify	284
Rarify and singletons removed	124
Rarify and singletons to tripletons removed	68

b

Parameter	Bray-Curtis similarity	Jaccard similarity
Replicate: 1 and 2		
All	0.39	0.28
Singletons removed	0.40	0.50
Singletons to tripletons removed	0.40	0.69
Rarify	0.38	0.30
Rarify and singletons removed	0.39	0.61
Rarify and singletons to tripletons removed	0.39	0.79
Replicate: 1 and 3		
All	0.30	0.29
Singletons removed	0.30	0.45
Singletons to tripletons removed	0.30	0.57
Rarify	0.30	0.23
Rarify and singletons removed	0.30	0.43
Rarify and singletons to tripletons removed	0.31	0.63
Replicate: 2 and 3		
All	0.16	0.24
Singletons removed	0.16	0.43
Singletons to tripletons removed	0.16	0.61
Rarify	0.16	0.23
Rarify and singletons removed	0.16	0.41
Rarify and singletons to tripletons removed	0.16	0.60

Figure S2. ITS sizes and their distribution of the representative sequences of *Castanopsis carlesii* deadwood mycobiomes.

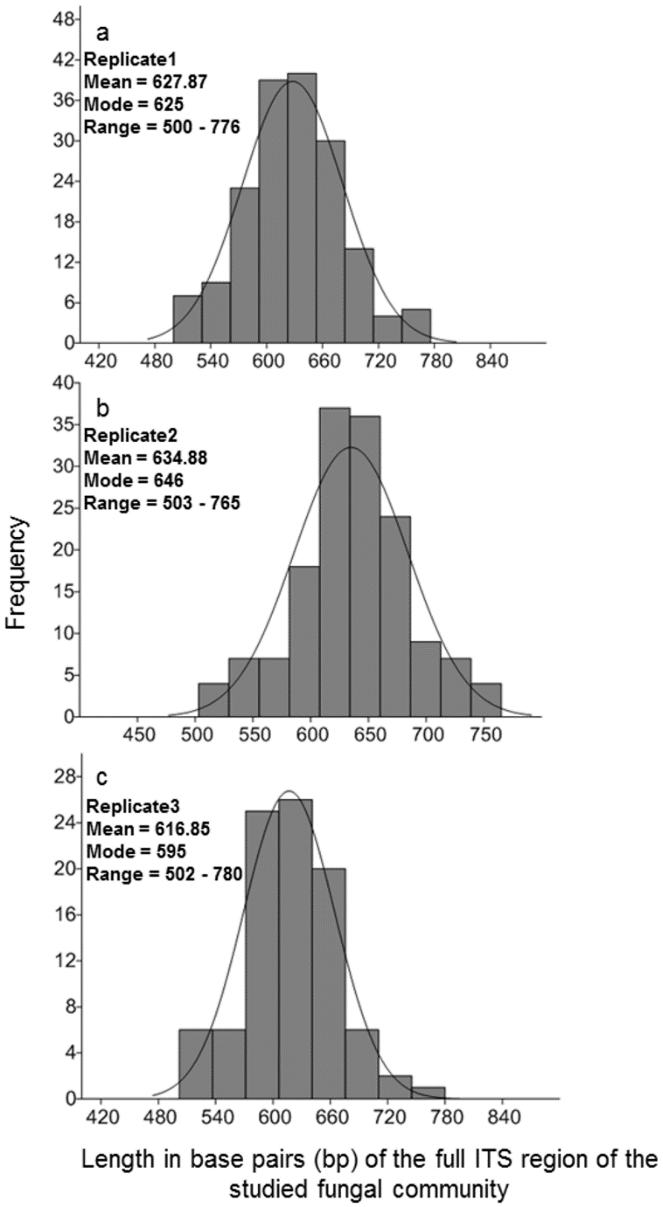

Figure S3. Rarefaction curves of rarified datasets using all OTUs (left panel) and all OTUs detected at least two sequences (right panel).

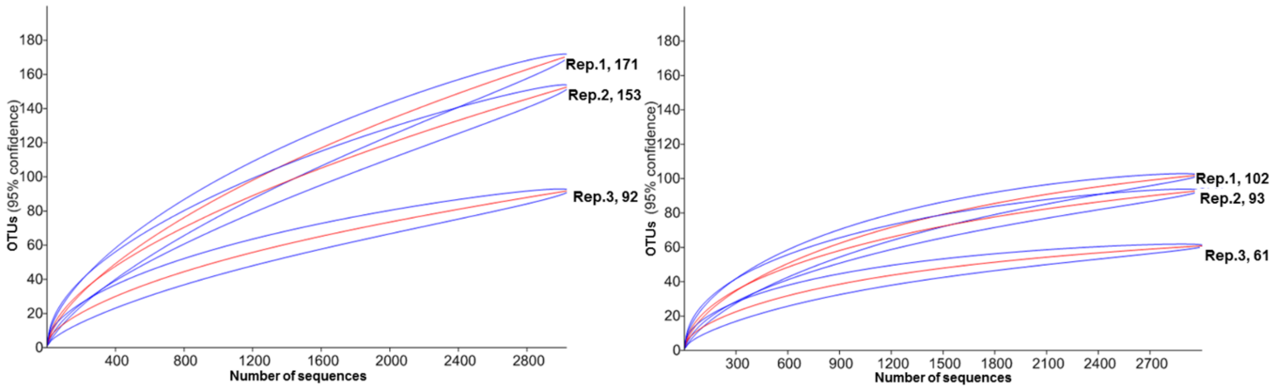

Figure S4. Phylograms of *Alternaria alternata* (Dothideomycetes) generated by Randomized Axelerated Maximum Likelihood (RAxML) analysis based on complete internal transcribed spacer (ITS) sequences.

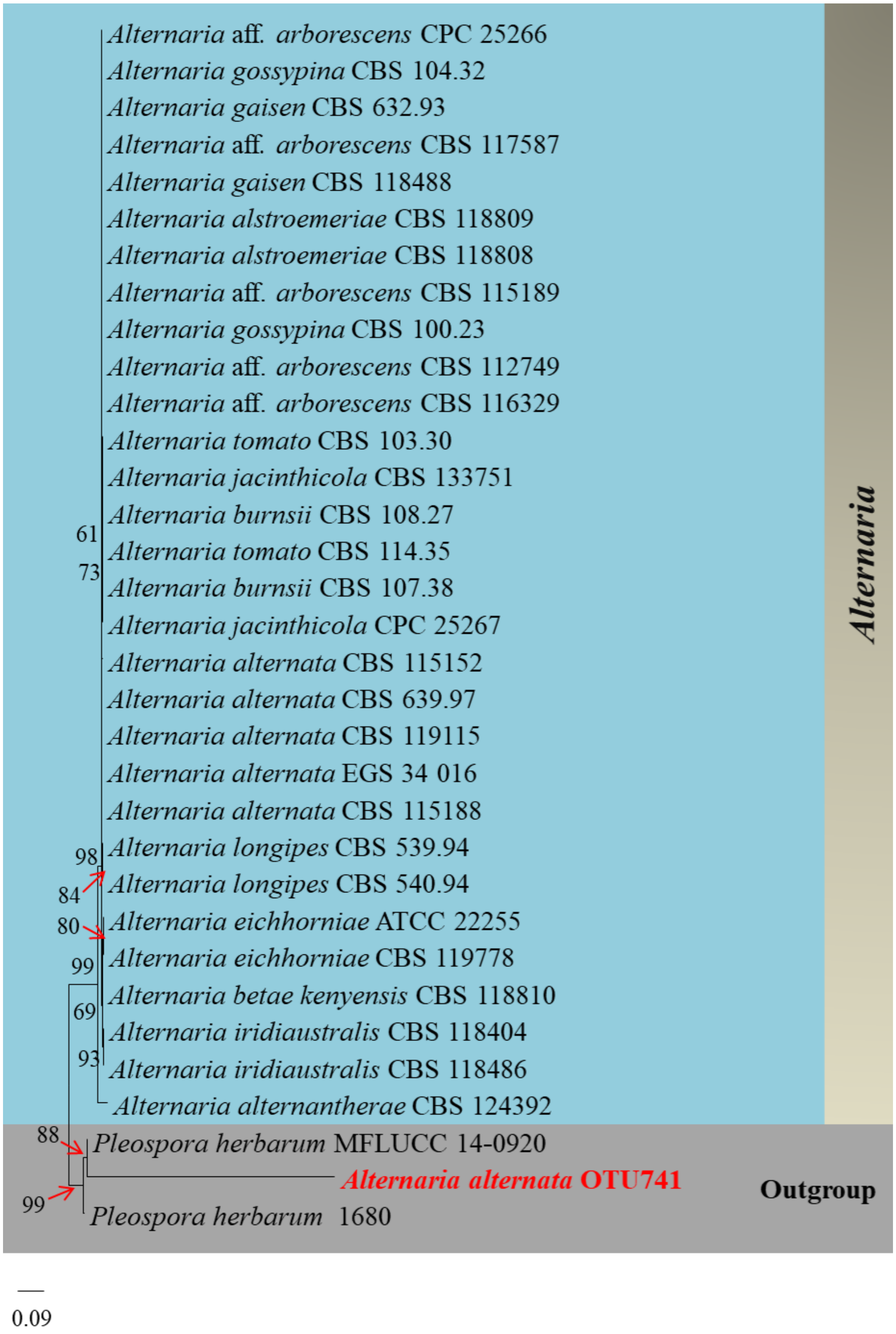

Figure S5. Phylograms of *Scytalidium lignicola* (Leotiomycetes) generated from Randomized Axelerated Maximum Likelihood (RAxML) analysis based on complete internal transcribed spacer (ITS) sequences.

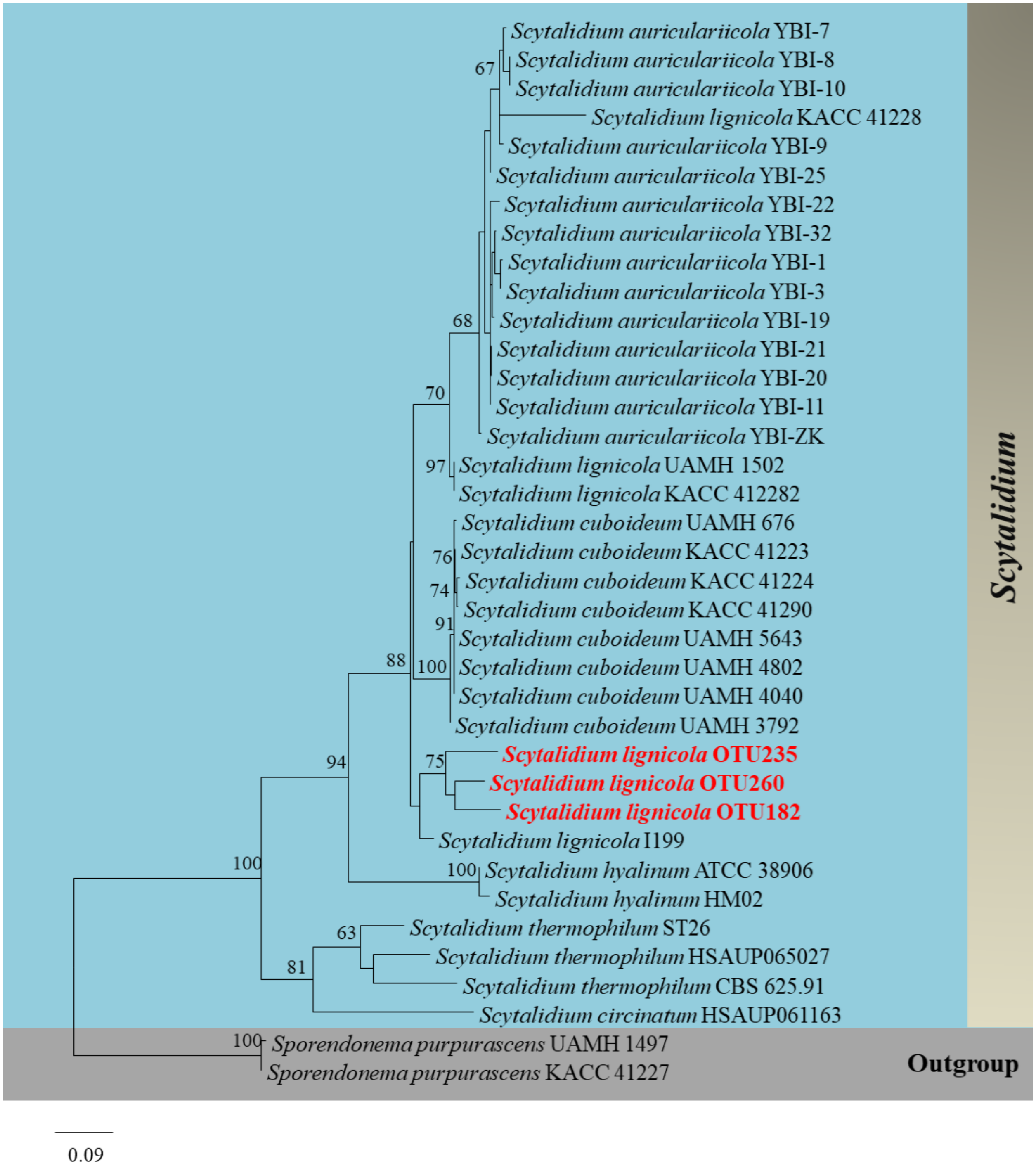

Figure S6. Phylograms of *Meliniomyces bicolor* (Leotiomycetes) generated from Randomized Axelerated Maximum Likelihood (RAxML) analysis based on complete internal transcribed spacer (ITS) sequences.

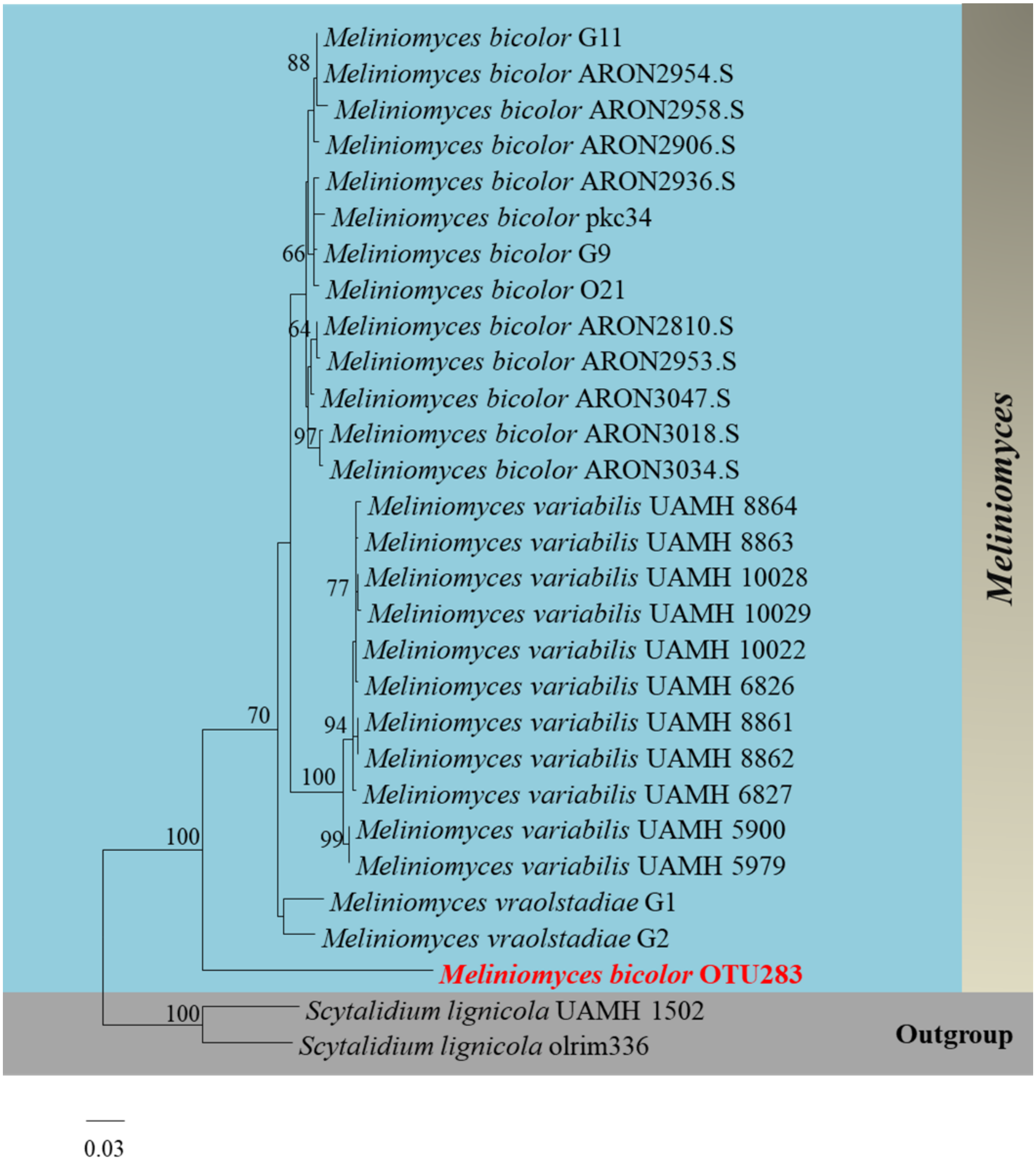

Figure S7. Phylograms of *Sugiyamaella novakii* (Saccharomycetes) generated from Randomized Axelerated Maximum Likelihood (RAxML) analysis based on complete internal transcribed spacer (ITS) sequences.

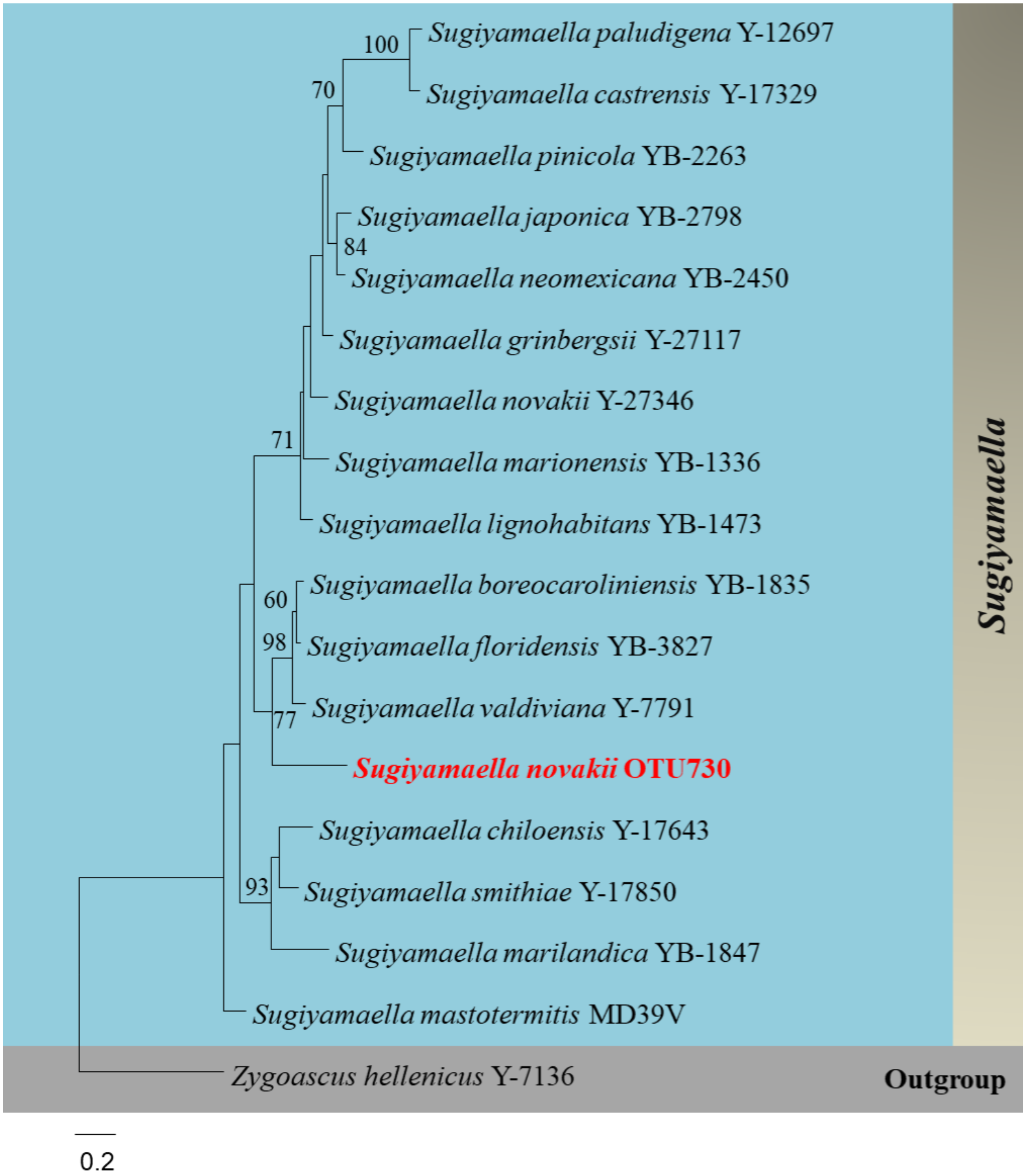

Figure S8. Phylograms of *Hawksworthiomyces crousii* (Sordariomycetes) generated from Randomized Axelerated Maximum Likelihood (RAxML) analysis based on complete internal transcribed spacer (ITS) sequences.

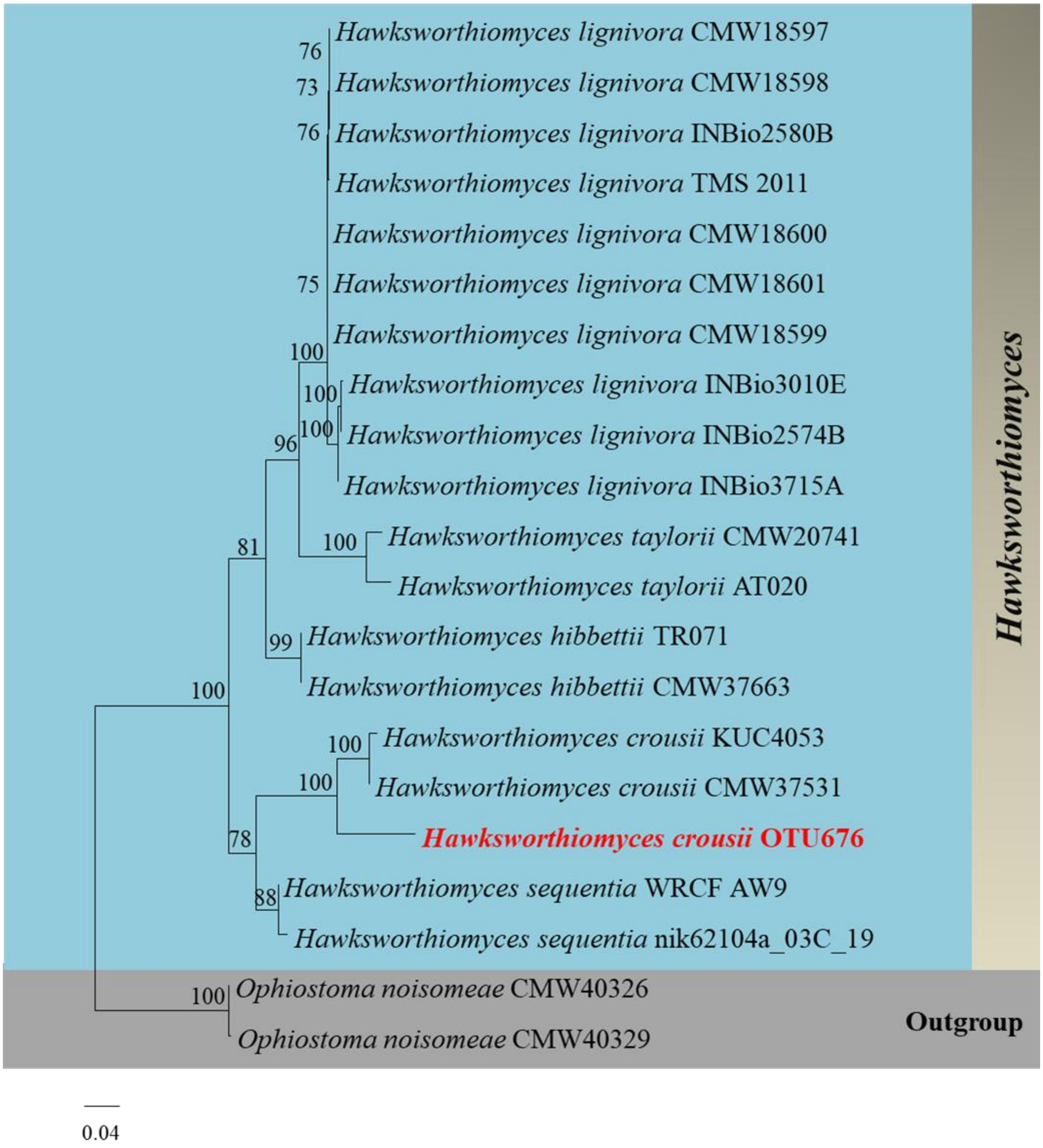

Figure S9. Phylograms of *Pestalotiopsis maculiformans* (Sordariomycetes) generated from Randomized Axelerated Maximum Likelihood (RAxML) analysis based on complete internal transcribed spacer (ITS) sequences.

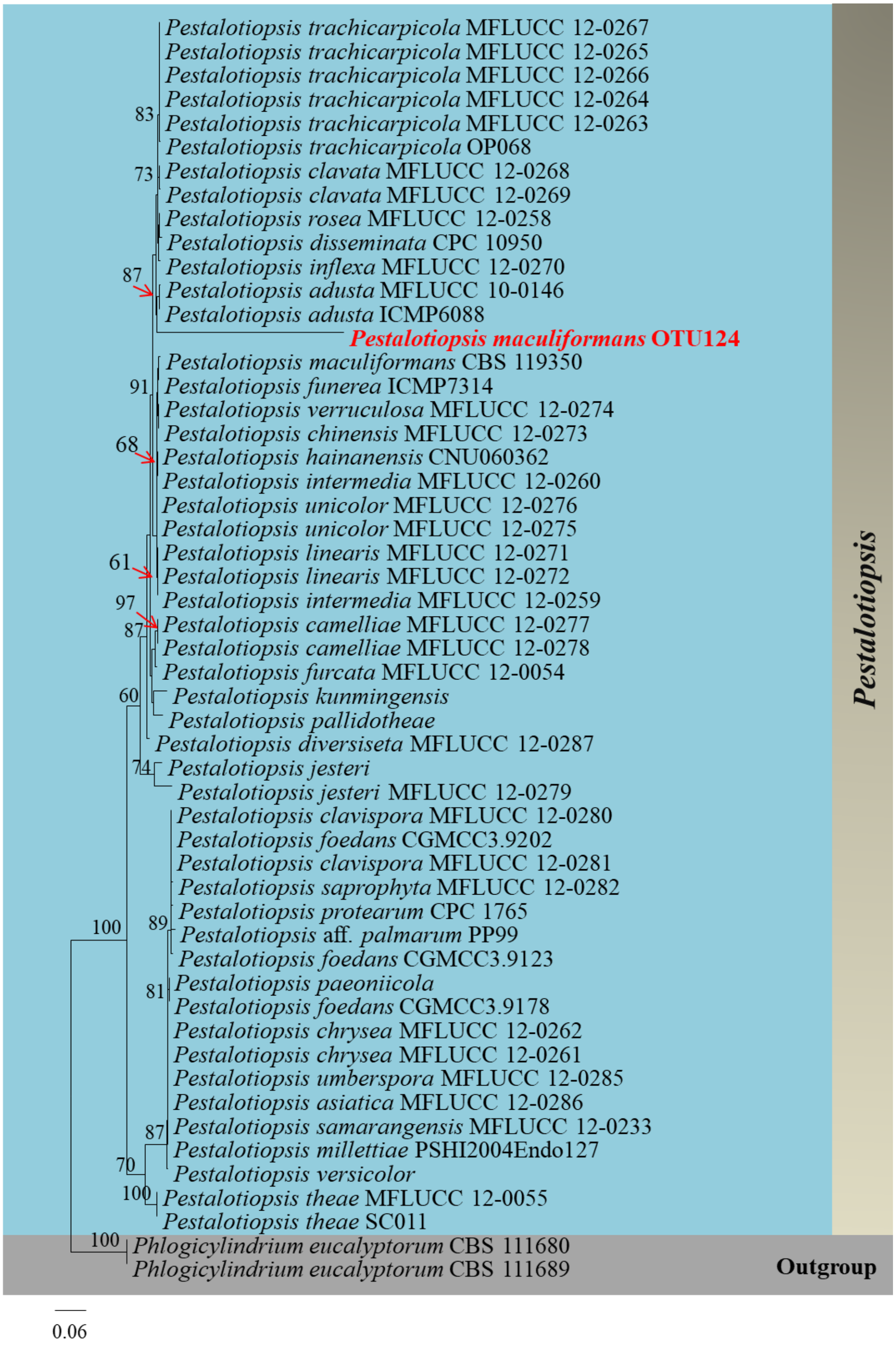

Figure S10. Phylograms of *Trichoderma harzianum* and *Trichoderma virens* (Sordariomycetes) generated from Randomized Axelerated Maximum Likelihood (RAxML) analysis based on complete internal transcribed spacer (ITS) sequences.

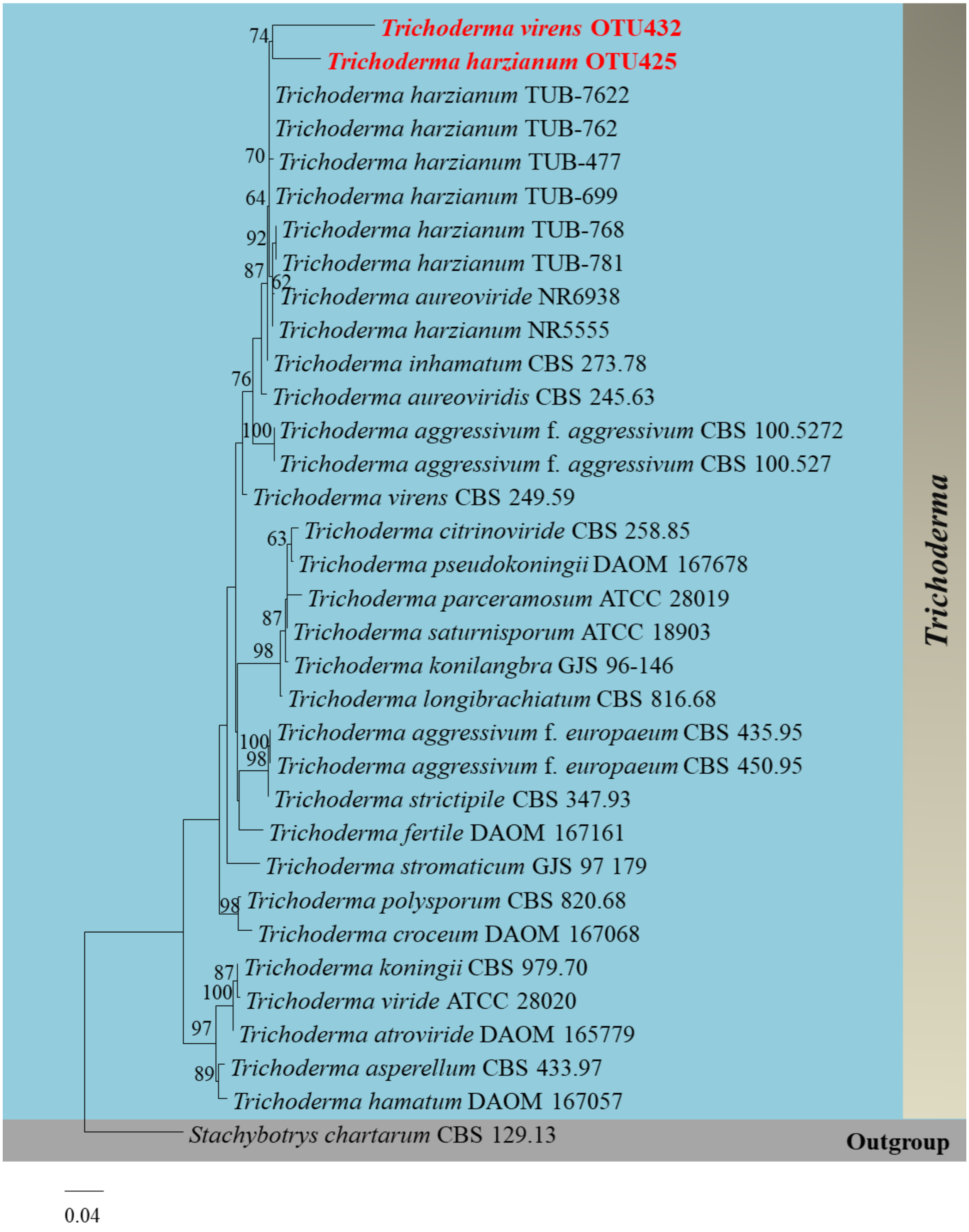

Figure S11. Phylograms of *Dictyochaeta simplex* (Sordariomycetes) generated from Randomized Axelerated Maximum Likelihood (RAxML) analysis based on complete internal transcribed spacer (ITS) sequences.

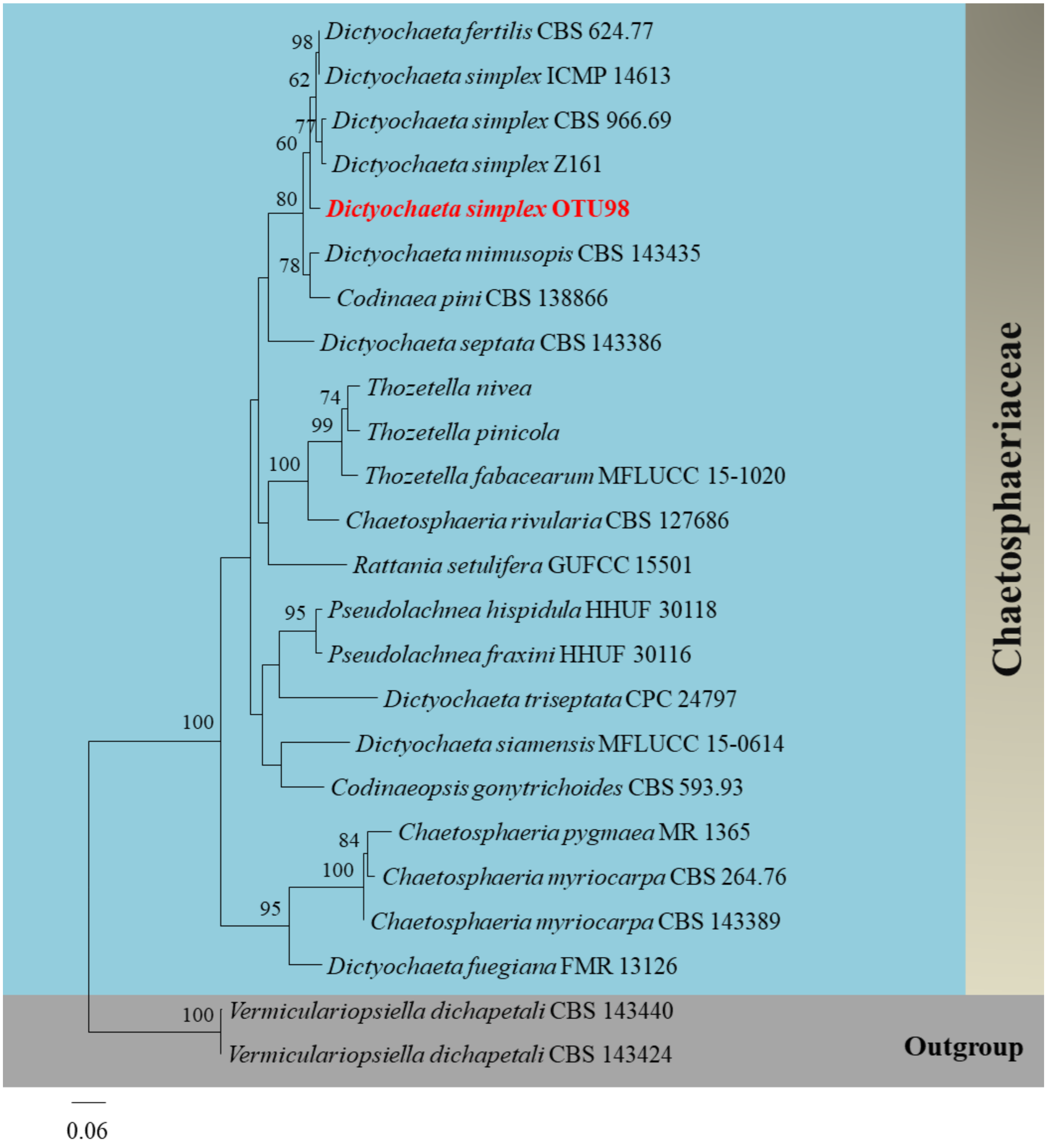

Figure S12. Phylograms of *Subulicystidium perlongisporum* and *Sistotremastrum guttuliferum* (Agaricomycetes, Trechisporales) generated from Randomized Axelerated Maximum Likelihood (RAxML) analysis based on complete internal transcribed spacer (ITS) sequences.

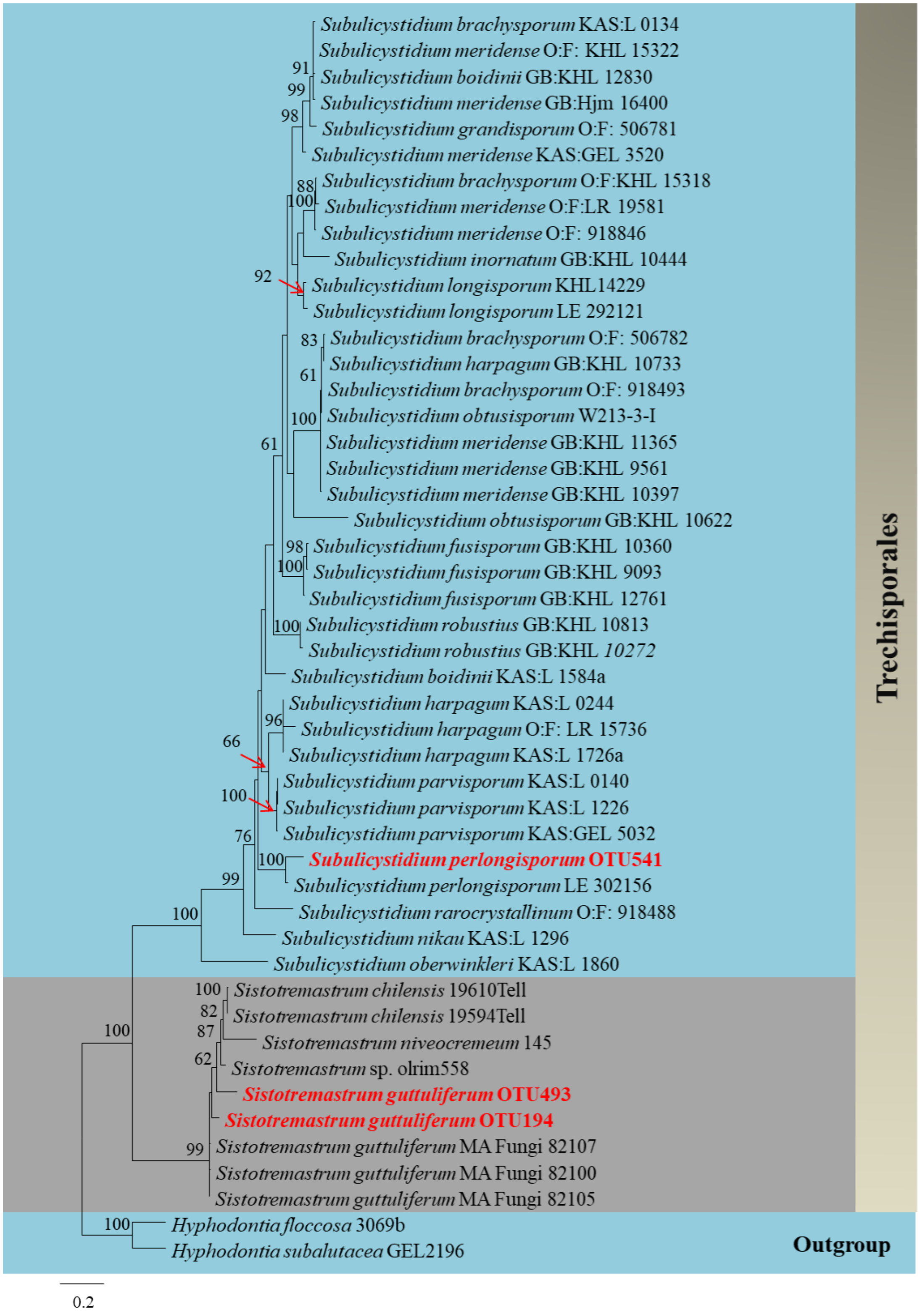

Figure S13. Phylograms of *Dichomitus squalens, Porogramme albocincta* and *Tinctoporellus epimiltinus* (Agaricomycetes, Polyporales) generated from Randomized Axelerated Maximum Likelihood (RAxML) analysis based on complete internal transcribed spacer (ITS) sequences.

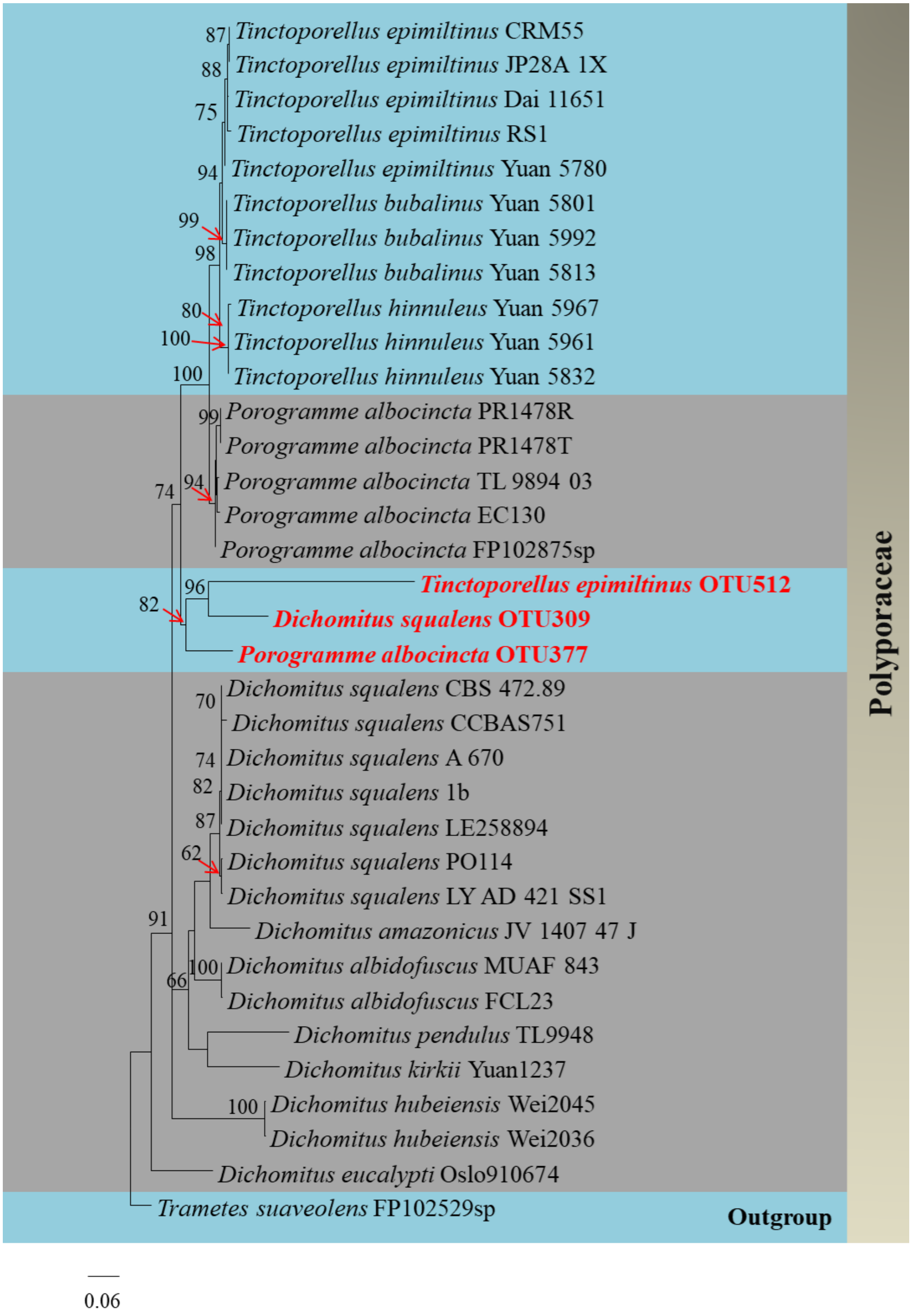

Figure S14. Phylograms of *Hymenochaete tongbiguanensis* and *Erythromyces crocicreas* (Agaricomycetes) generated from Randomized Axelerated Maximum Likelihood (RAxML) analysis based on complete internal transcribed spacer (ITS) sequences.

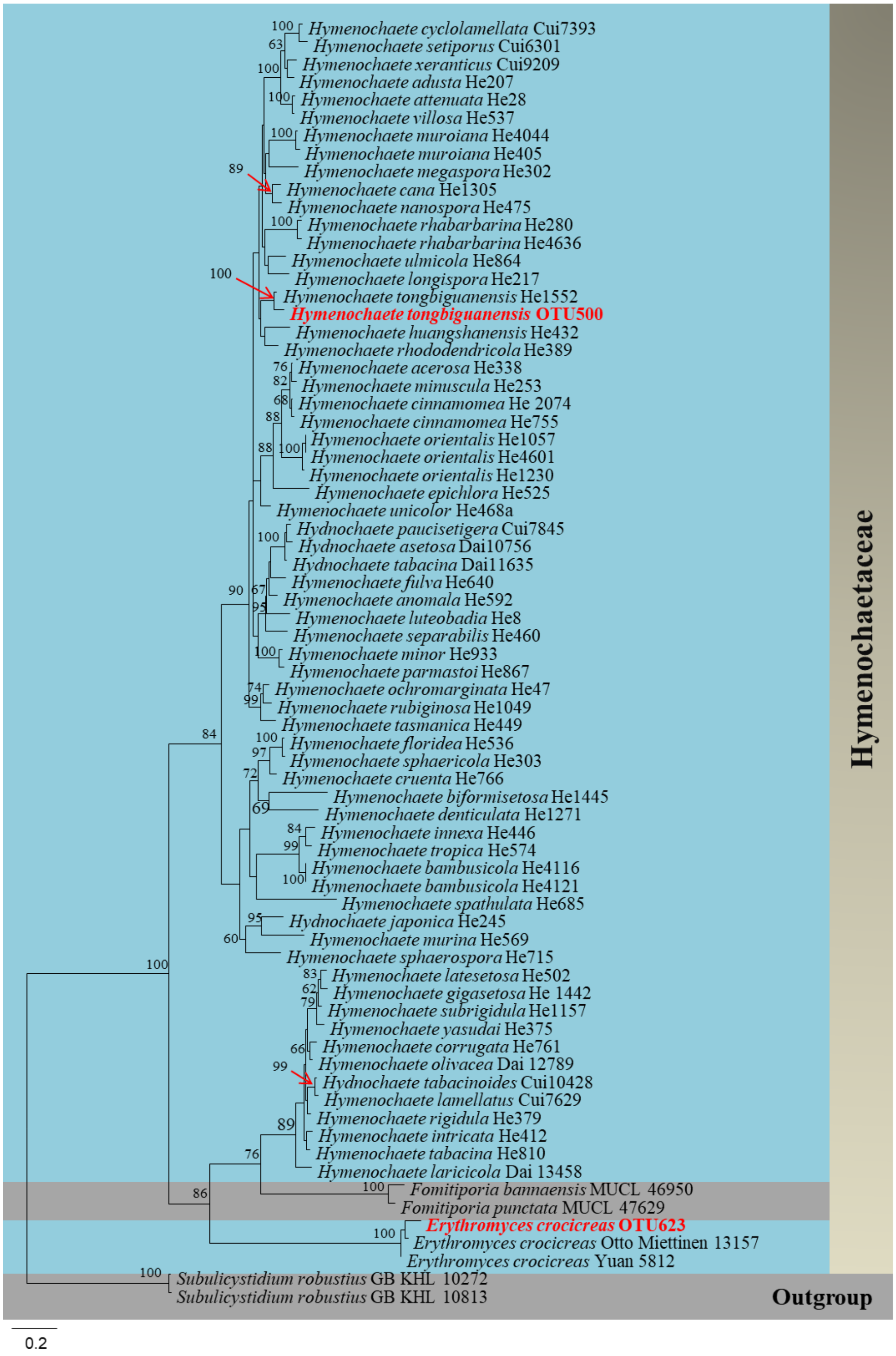

Figure S15. Phylograms of *Jianyunia sakaguchii* (Agaricostilbomycetes) generated from Randomized Axelerated Maximum Likelihood (RAxML) analysis based on complete internal transcribed spacer (ITS) sequences.

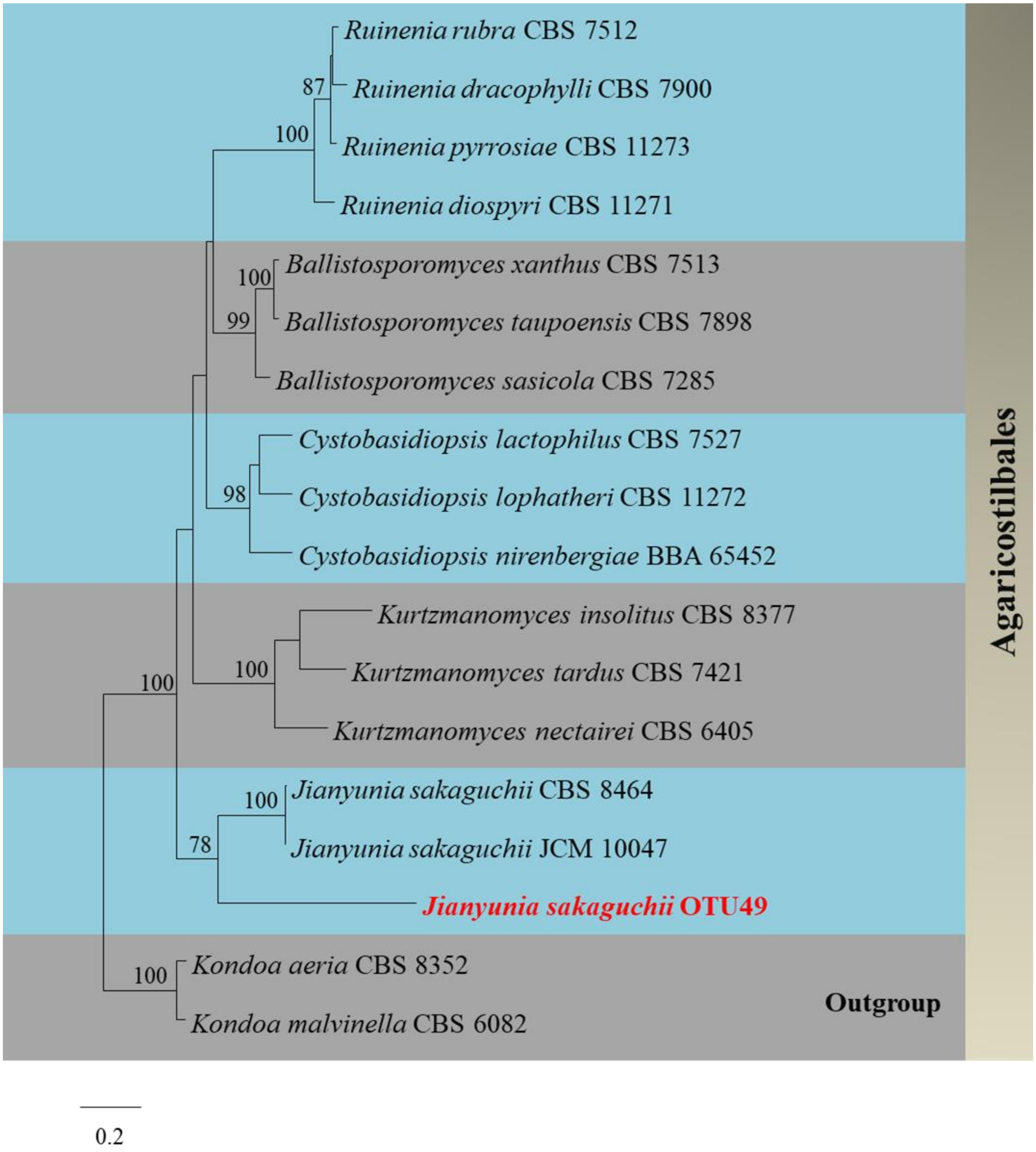

Supplement: Supplementary file 1 [file Data_Sheet_1.pdf]
