# Supplementary material for: Characterization of the Castanopsis carlesii Deadwood Mycobiome by Pacbio Sequencing of the Full-Length Fungal Nuclear Ribosomal Internal Transcribed Spacer (ITS)
Source: Front Microbiol. 2019 May 17;10:983. doi: 10.3389/fmicb.2019.00983 (PMC6540943; doi:10.3389/fmicb.2019.00983)
Supplement: Supplementary file 2 [file Data_Sheet_2.pdf]

Supplementary materials

Characterization of the *Castanopsis carlesii* deadwood mycobiome by Pacbio sequencing of the full-length fungal nuclear ribosomal internal transcribed spacer (ITS)

Author names:

Witoon Purahong[1], Ausana Mapook[1, 2], Yu-Ting Wu[3], Chaur-Tzuhn Chen[3]

Affiliations:

[1]Department of Soil Ecology, UFZ-Helmholtz Centre for Environmental Research, Theodor-Lieser-Str. 4, D-06120 Halle (Saale), Germany; [2]Center of Excellence in Fungal Research, Mae Fah Luang University, Chiang Rai 57100, Thailand , [3]Department of Forestry, National Pingtung University of Science and Technology, Pingtung, Taiwan

Appendix 1: Community composition, richness, and taxonomic identification of the *Castanopsis carlesii* deadwood mycobiome based on the ITS1, ITS2, and full-length ITS sequences

Table S1. Taxonomic information of all wood-inhabiting fungi identified at species level based on Pacbio

Full ITS (36 OTUs identified at species level)	ITS1 (46 OTUs identified at species level)	ITS2 (41 OTUs identified at species level)
Alternaria alternata	*Acremoniopsis suttonii*	*Campylocarpon fasciculare*
Campylocarpon fasciculare	*Colacogloea terpenoidalis*	*Colacogloea terpenoidalis*
Colacogloea terpenoidalis	*Devriesia strelitziicola*	*Devriesia strelitziicola*
Coprinellus disseminatus	*Dictyochaeta simplex*	*Dichomitus albidofuscus*
Devriesia strelitziicola	*Erythromyces crocicreas*	*Erythromyces crocicreas*
Dichomitus squalens	*Erythromyces crocicreas*	*Erythromyces crocicreas*
Dictyochaeta simplex	*Hawksworthiomyces crousii*	*Fusarium oxysporum*
Erythromyces crocicreas	*Hymenochaete tongbiguanensis*	*Ganoderma tsugae*
Hawksworthiomyces crousii	*Nakazawaea ishiwadae*	*Hawksworthiomyces crousii*
Hymenochaete tongbiguanensis	*Pestalotiopsis palustris*	*Hymenochaete tongbiguanensis*
Jianyunia sakaguchii	*Pestalotiopsis vismiae*	*Hymenochaete xerantica*
Meliniomyces bicolor	*Pilidium eucalyptorum*	*Jianyunia sakaguchii*
Nectria aurantiaca	*Piskurozyma taiwanensis*	*Nakazawaea ishiwadae*
Pestalotiopsis maculiformans	*Porogramme albocincta*	*Neopestalotiopsis javaensis*
Pilidium concavum	*Pseudohyphozyma bogoriensis*	*Pestalotiopsis microspora*
Pilidium eucalyptorum	*Scytalidium cuboideum*	*Pilidium acerinum*
Pilidium pseudoconcavum	*Scytalidium lignicola*	*Pilidium eucalyptorum*

**Full ITS (36 OTUs identified at species level)**	**ITS1 (46 OTUs identified at species level)**	**ITS2 (41 OTUs identified at species level)**
Porogramme albocincta	*Scytalidium lignicola*	*Piskurozyma taiwanensis*
Pseudohyphozyma bogoriensis	*Scytalidium lignicola*	*Porogramme albocincta*
Rhodotorula cycloclastica	*Scytalidium lignicola*	*Rhodotorula cycloclastica*
Scytalidium lignicola	*Scytalidium lignicola*	*Scytalidium lignicola*
Scytalidium lignicola	*Sistotremastrum guttuliferum*	*Sistotremastrum guttuliferum*
Scytalidium lignicola	*Sistotremastrum guttuliferum*	*Sistotremastrum guttuliferum*
Sistotremastrum guttuliferum	*Sistotremastrum guttuliferum*	*Sugiyamaella chiloensis*
Sistotremastrum guttuliferum	*Sugiyamaella smithiae*	*Sugiyamaella smithiae*
Subulicystidium perlongisporum	*Symbiotaphrina kochii*	*Symbiotaphrina kochii*
Sugiyamaella novakii	*Tinctoporellus epimiltinus*	*Tremella foliacea*
Symbiotaphrina kochii	*Tremella foliacea*	*Trichoderma harzianum*
Tinctoporellus epimiltinus	*Trichoderma harzianum*	*Trichoderma harzianum*
Tremella fuciformis	*Trichoderma harzianum*	*Trichoderma harzianum*
Trichoderma harzianum	*Trichoderma harzianum*	*Trichoderma harzianum*
Trichoderma virens	*Trichoderma harzianum*	*Trichoderma harzianum*
Trichosphaerella ceratophora	*Trichoderma harzianum*	*Trichoderma harzianum*
Xenasmatella ardosiaca	*Trichoderma harzianum*	*Trichoderma harzianum*
Xenoacremonium falcatum	*Trichoderma harzianum*	*Trichoderma harzianum*

Full ITS (36 OTUs identified at species level)	ITS1 (46 OTUs identified at species level)	ITS2 (41 OTUs identified at species level)
Xenoacremonium recifei	*Trichoderma harzianum*	*Trichoderma harzianum*
	Trichoderma harzianum	*Trichoderma lixii*
	Trichoderma harzianum	*Trichoderma virens*
	Trichoderma harzianum	*Trichosphaerella ceratophora*
	Trichoderma harzianum	*Xenasmatella ardosiaca*
	Trichoderma inhamatum	*Xenoacremonium falcatum*
	Trichoderma virens	
	Veronaea botryosa	
	Xenasmatella ardosiaca	
	Xenasmatella ardosiaca	
	Xenoacremonium falcatum	

*******Dark green highlight = detected using Pacbio based on the ITS1, ITS2, and full-length ITS. Light green highlight = detected using Pacbio based on the full-length ITS and ITS1 or ITS2. Red highlight = only detected using Pacbio based on the full-length ITS.

Table S2. Comparison of fungal taxonomic information of 36 wood-inhabiting fungi identified at species level based on Pacbio sequencing alone and Pacbio sequencing incorporated with phylogenetic analysis based on Randomized Axelerated Maximum Likelihood (RAxML) of the complete internal transcribed spacer (ITS) sequence.

ID	Taxonomy based on ITS1 phylogeny	Bootstrap	Taxonomy based on ITS2 phylogeny	Bootstrap	Taxonomy based on Pacbio	Taxonomy based on Pacbio and phylogeny	Bootstrap
Devriesia OTU219	***Devriesia strelitziicola***	100%	***Devriesia strelitziicola***	100%	*Devriesia strelitziicola*	***Devriesia strelitziicola***	100%
Alternaria OTU741	Multiple taxa including *Alternaria* spp.	-	Multiple taxa including *Alternaria* spp.	-	*Alternaria alternata*	*Pleospora herbarum*	88%
Pilidium OTU445	*Pilidium pseudoconcavum*	33%	*Pilidium eucalyptorum*	59%	*Pilidium concavum*	*Pilidium eucalyptorum*	90%
Pilidium OTU479	*Pilidium pseudoconcavum*	33%	*Pilidium eucalyptorum*	59%	*Pilidium pseudoconcavum*	*Pilidium eucalyptorum*	90%
Pilidium OTU632	*Pilidium pseudoconcavum*	33%	*Pilidium eucalyptorum*	59%	*Pilidium eucalyptorum*	*Pilidium eucalyptorum*	90%
Scytalidium OTU182	Multiple taxa including *Scytalidium* spp.	-	*Scytalidium lignicola*	56%	*Scytalidium lignicola*	*Scytalidium lignicola*	<60%
Scytalidium OTU235	Multiple taxa including *Scytalidium* spp.	-	*Scytalidium lignicola*	56%	*Scytalidium lignicola*	*Scytalidium lignicola*	<60%
Scytalidium OTU260	Multiple taxa including *Scytalidium* spp.	-	*Scytalidium lignicola*	56%	*Scytalidium lignicola*	*Scytalidium lignicola*	<60%
Meliniomyces OTU283	Multiple taxa including *Meliniomyces* spp.	-	Multiple taxa including *Meliniomyces spp.*	-	*Meliniomyces bicolor*	*Separate from existing Meliniomyces sp.*	100%
Sugiyamaella OTU730	*Sugiyamaella* sp.	17%	Multiple taxa including *Sugiyamaella* spp.	44%	*Sugiyamaella novakii*	*Sugiyamaella spp.*	77%
Campylocarpon OTU403	*Campylocarpon pseudofasciculare*	75%	*Campylocarpon fasciculare*	69%	*Campylocarpon fasciculare*	***Campylocarpon fasciculare***	100%
Trichosphaerella OTU525	*Trichosphaerella ceratophora*	86%	***Trichosphaerella ceratophora***	100%	*Trichosphaerella ceratophora*	***Trichosphaerella ceratophora***	100%
Hawksworthiomyces OTU676	*Hawksworthiomyces crousii*	74%	***Hawksworthiomyces crousii***	100%	*Hawksworthiomyces crousii*	***Hawksworthiomyces crousii***	100%
Pestalotiopsis OTU124	*Pestalotiopsis spp.*	14%	*Pestalotiopsis australis*	26%	*Pestalotiopsis maculiformans*	*Pestalotiopsis adusta*	87%
Trichoderma OTU425	*Trichoderma aureoviride*	50%	*Trichoderma spp.*	62%	*Trichoderma harzianum*	*Trichoderma harzianum*	74%
Trichoderma OTU432	*Trichoderma aureoviride*	50%	*Trichoderma spp.*	21%	*Trichoderma virens*	*Trichoderma harzianum*	74%
Xenoacremonium OTU524	*Xenoacremonium recifei Xenoacremonium falcatus*	37%	*Xenoacremonium recifei*	48%	*Xenoacremonium recifei*	*Xenoacremonium recifei*	62%
Dictyochaeta OTU98	Multiple taxa including *Dictyochaeta spp.*	-	*Codinaea pini*	53%	*Dictyochaeta simplex*	*Dictyochaeta simplex*	60%
Nectria OTU266	*Xenoacremonium recifei Xenoacremonium falcatus*	38%	*Xenoacremonium recifei*	26%	*Nectria aurantiaca*	*Nectria pseudotrichia*	<60%

ID	Taxonomy based on ITS1 phylogeny	Bootstrap	Taxonomy based on ITS2 phylogeny	Bootstrap	Taxonomy based on Pacbio	Taxonomy based on Pacbio and phylogeny	Bootstrap
Xenoacremonium OTU522	*Xenoacremonium recifei Xenoacremonium falcatus*	38%	*Xenoacremonium recifei Xenoacremonium falcatus*	56%	*Xenoacremonium falcatus*	*Xenoacremonium falcatus*	<60%
Symbiotaphrina OTU423	*Symbiotaphrina kochii*	57%	***Symbiotaphrina kochii***	100%	*Symbiotaphrina kochii*	***Symbiotaphrina kochii***	100%
Coprinellus OTU515	*Coprinellus disseminatus*	92%	***Coprinellus disseminatus***	100%	*Coprinellus disseminatus*	***Coprinellus disseminatus***	100%
Erythromyces OTU623	*Erythromyces crocicreas*	72%	***Erythromyces crocicreas***	100%	*Erythromyces crocicreas*	***Erythromyces crocicreas***	100%
Hymenochaete OTU500	*Hymenochaete tongbiguanensis*	96%	*Hymenochaete tongbiguanensis*	93%	*Hymenochaete tongbiguanensis*	***Hymenochaete tongbiguanensis***	100%
Subulicystidium OTU541	*Subulicystidium perlongisporum*	94%	*Subulicystidium perlongisporum*	93%	*Subulicystidium perlongisporum*	***Subulicystidium perlongisporum***	100%
Sistotremastrum OTU194	*Sistotremastrum guttuliferum*	86%	*Sistotremastrum spp.*	-	*Sistotremastrum guttuliferum*	***Sistotremastrum guttuliferum***	99%
Sistotremastrum OTU493	*Sistotremastrum spp.*	-	*Sistotremastrum spp.*	-	*Sistotremastrum guttuliferum*	***Sistotremastrum guttuliferum***	99%
Xenasmatella OTU488	*Xenasmatella ardosiaca*	72%	*Xenasmatella ardosiaca*	59%	*Xenasmatella ardosiaca*	*Xenasmatella ardosiaca*	90%
Dichomitus OTU309	Multiple taxa including *Porogramme albocincta and Tinctoporellus spp.*	-	Multiple taxa including *Porogramme albocincta and Tinctoporellus spp.*	-	*Dichomitus squalens*	*Tinctoporellus sp. and Porogramme sp. clade*	74%
Porogramme OTU377	Multiple taxa including *Porogramme albocincta and Tinctoporellus spp.*	-	Multiple taxa including *Porogramme albocincta and Tinctoporellus spp.*	-	*Porogramme albocincta*	*Tinctoporellus sp. and Porogramme sp. clade*	74%
Tinctoporellus OTU512	Multiple taxa including *Porogramme albocincta and Tinctoporellus spp.*	-	Multiple taxa including *Porogramme albocincta and Tinctoporellus spp.*	-	*Tinctoporellus epimiltinus*	*Tinctoporellus sp. and Porogramme sp. clade*	74%
Jianyunia OTU49	*Jianyunia sakaguchii Bensingtonia sakaguchii*	38%	*Jianyunia sakaguchii Bensingtonia sakaguchii*	87%	*Jianyunia sakaguchii*	*Jianyunia sakaguchii*	78%
Colacogloea OTU48	*Colacogloea terpenoidalis*	43%	*Colacogloea terpenoidalis*	85%	*Colacogloea terpenoidalis*	***Colacogloea terpenoidalis***	100%
Colacogloea OTU50	*Colacogloea cycloclastica*	97%	***Colacogloea cycloclastica***	100%	*Colacogloea cycloclastica*	***Colacogloea cycloclastica***	100%
Pseudohyphozyma OTU45	Multiple taxa including *Pseudohyphozyma bogoriensis*	-	***Pseudohyphozyma bogoriensis***	100%	*Pseudohyphozyma bogoriensis*	***Pseudohyphozyma bogoriensis***	100%
Tremella OTU709	*Tremella spp.*	66%	*Tremella foliacea*	51%	*Tremella fuciformis*	*Tremella spp.*	100%

*******Green highlight = identical identification at species level (bold letters indicate 99 – 100% Bootstrap support). Orange highlight = identical identification at genus level. Red highlight = different identification at genus level.

Figure S16. Information on similarity of community composition in *Castanopsis carlesii* deadwood mycobiomes subjected to different bioinformatics parameters (rarified and rarified with singletons removed).

	Full-length ITS		ITS1		ITS2	
**Parameter**	**Bray-Curtis similarity**	**Jaccard similarity**	**Bray-Curtis similarity**	**Jaccard similarity**	**Bray-Curtis similarity**	**Jaccard similarity**
Replicate: 1 and 2						
Rarify	**0.38**	**0.30**	**0.37**	**0.25**	**0.38**	**0.25**
Rarify and singletons removed	**0.39**	**0.61**	0.38	**0.47**	**0.39**	**0.52**
Replicate: 1 and 3						
Rarify	**0.30**	**0.23**	**0.30**	**0.24**	**0.27**	**0.22**
Rarify and singletons removed	**0.30**	**0.43**	**0.30**	**0.43**	**0.28**	**0.40**
Replicate: 2 and 3						
Rarify	**0.16**	**0.23**	**0.14**	**0.19**	**0.17**	**0.22**
Rarify and singletons removed	**0.16**	**0.41**	**0.14**	**0.32**	**0.17**	**0.40**

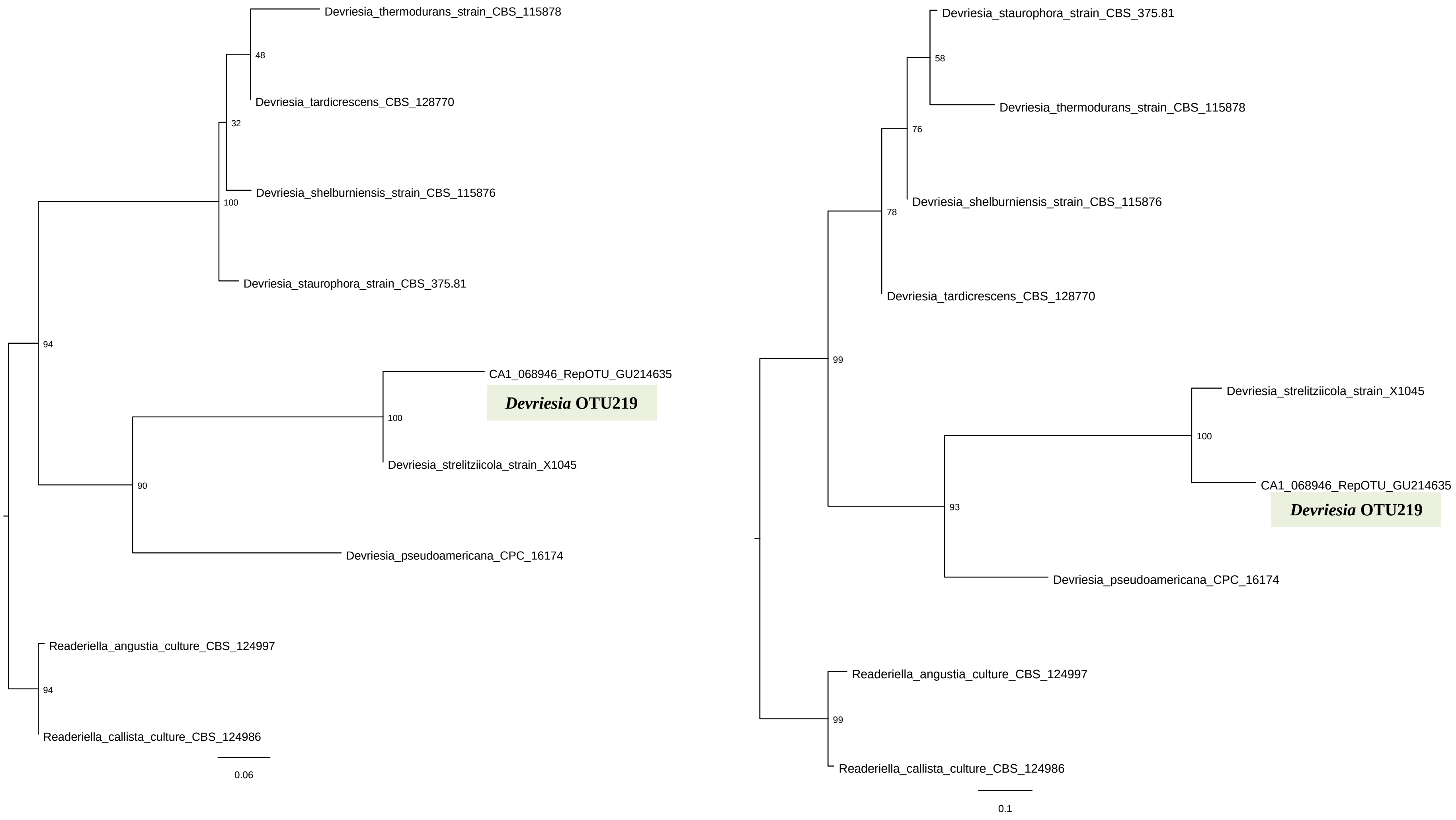

Figure S17. Phylograms of *Devriesia* OTU219 (Dothideomycetes) generated from Randomized Axelerated Maximum Likelihood (RAxML) analysis based on internal transcribed spacer 1 (ITS1, left) or on internal transcribed spacer 2 (ITS2; right) sequence.

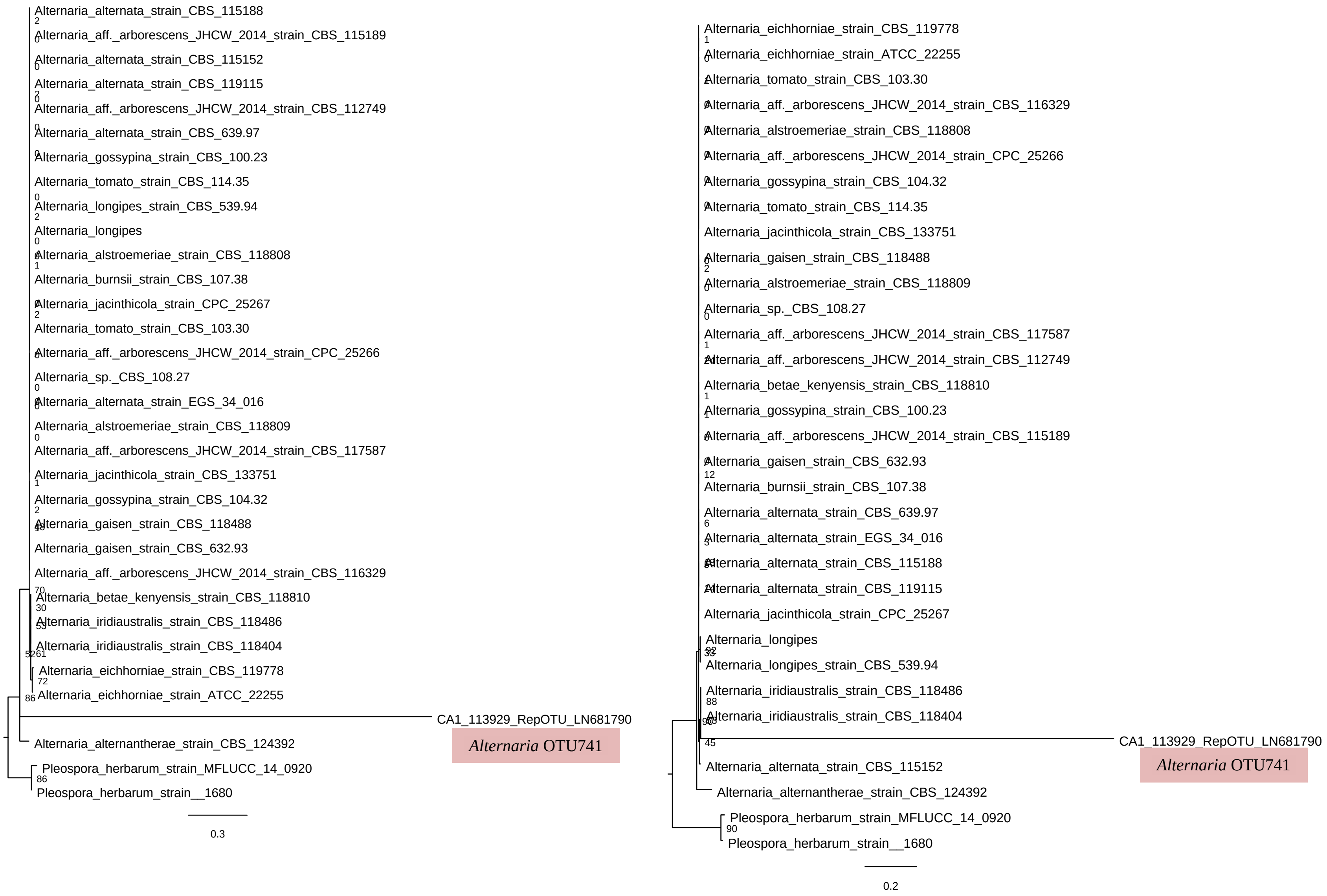

Figure S18. Phylograms of *Alternaria* OTU741 (Dothideomycetes) generated from Randomized Axelerated Maximum Likelihood (RAxML) analysis based on internal transcribed spacer 1 (ITS1, left) or on internal transcribed spacer 2 (ITS2; right) sequence.

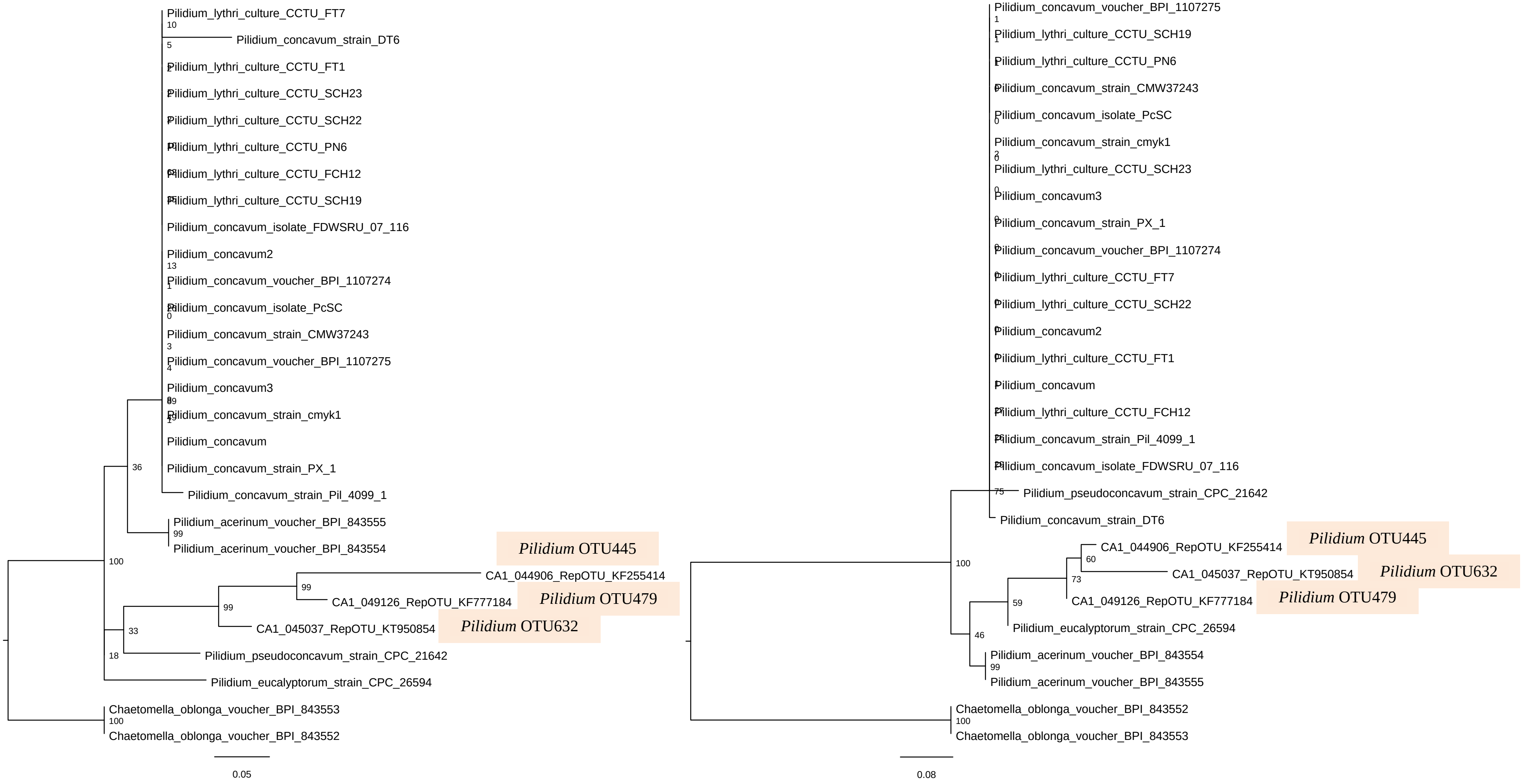

Figure S19. Phylograms of *Pilidium* OTU445, OTU479, OTU632 (Leotiomycetes) generated from Randomized Axelerated Maximum Likelihood (RAxML) analysis based on internal transcribed spacer 1 (ITS1, left) or on internal transcribed spacer 2 (ITS2; right) sequence.

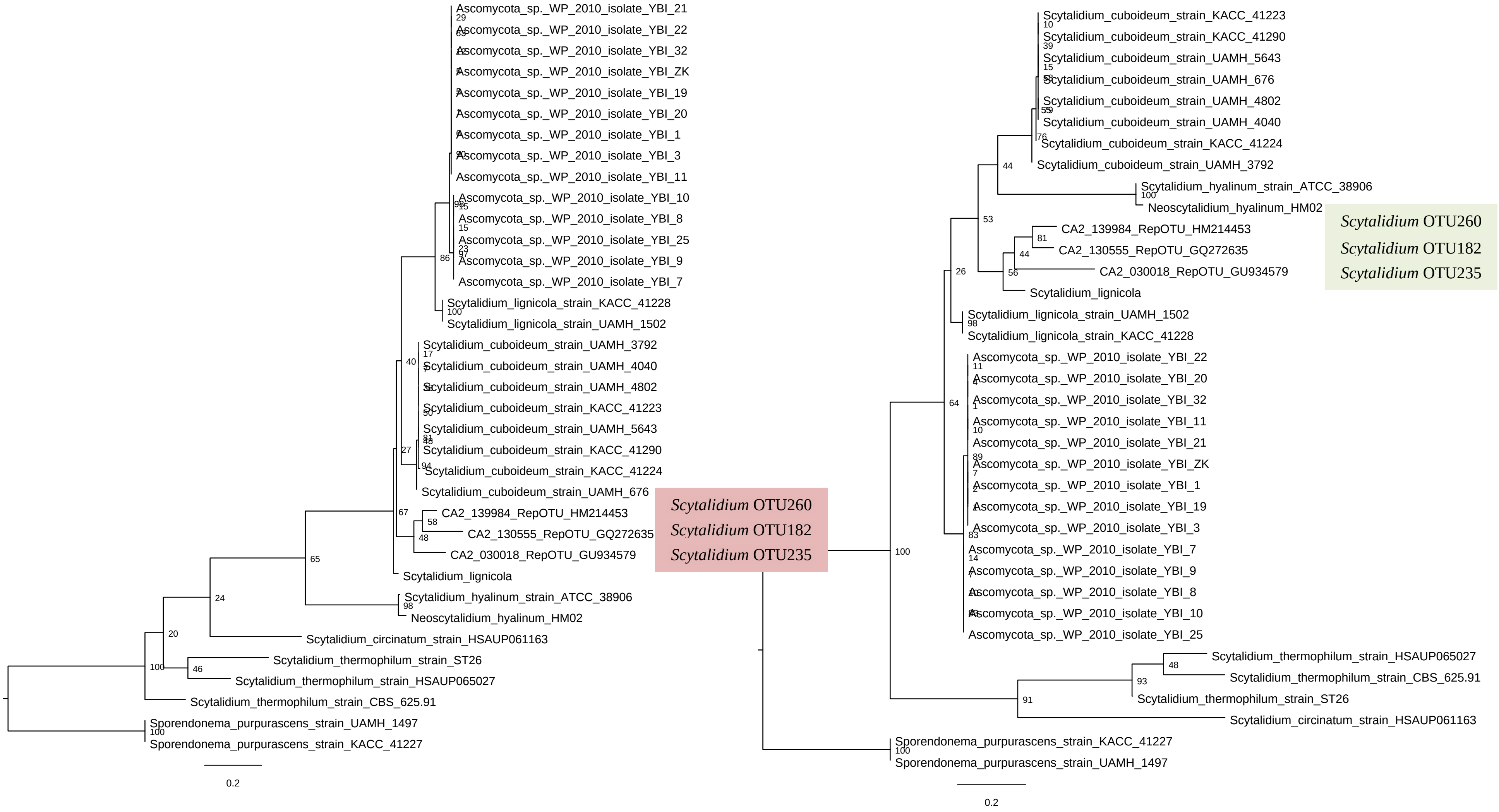

Figure S20. Phylograms of *Scytalidium* OTU182, OTU235, OTU260 (Leotiomycetes) generated from Randomized Axelerated Maximum Likelihood (RAxML) analysis based on internal transcribed spacer 1 (ITS1, left) or on internal transcribed spacer 2 (ITS2; right) sequence.

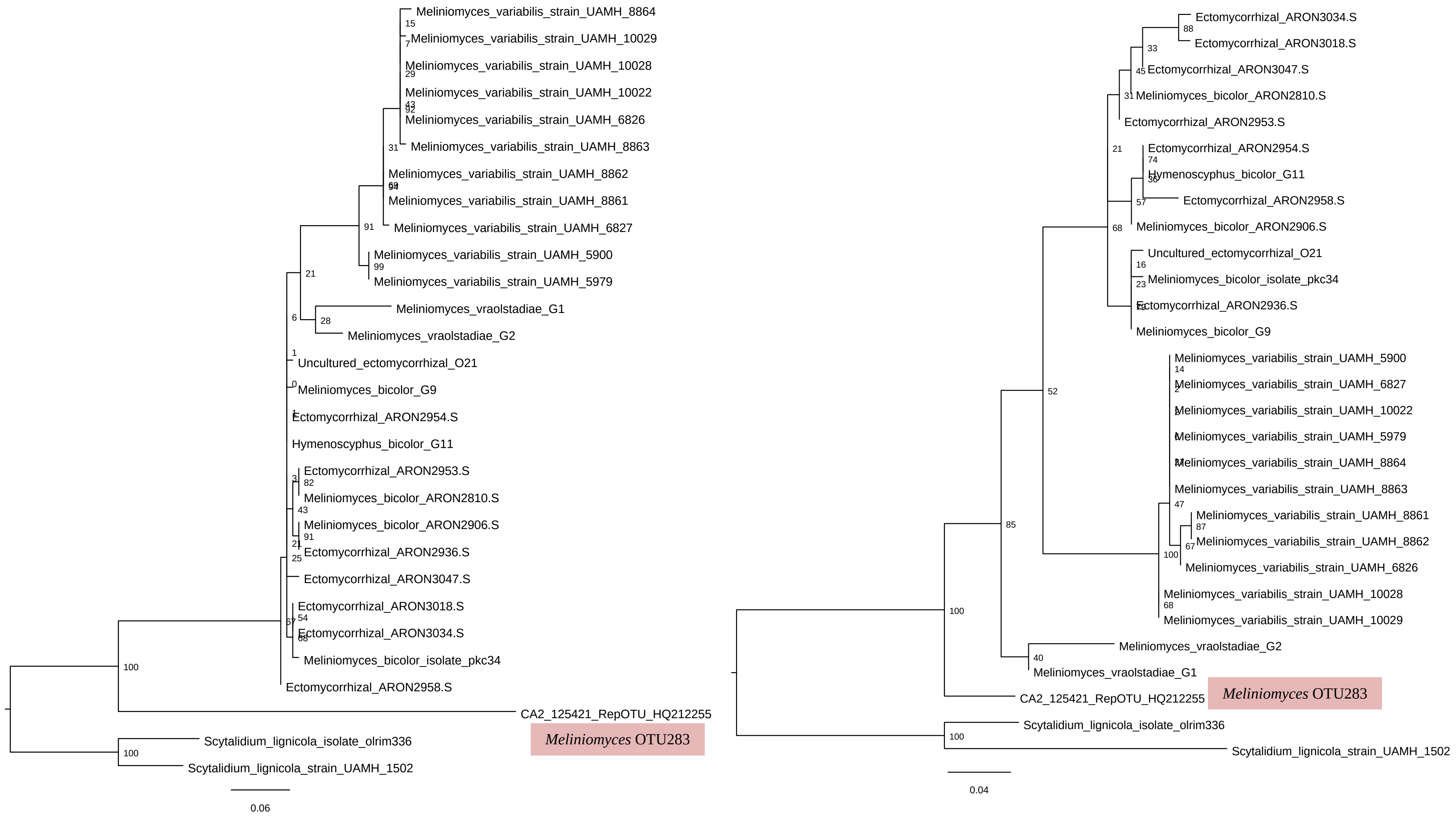

Figure S21. Phylograms of *Meliniomyces* OTU283 (Leotiomycetes) generated from Randomized Axelerated Maximum Likelihood (RAxML) analysis based on internal transcribed spacer 1 (ITS1, left) or on internal transcribed spacer 2 (ITS2; right) sequence.

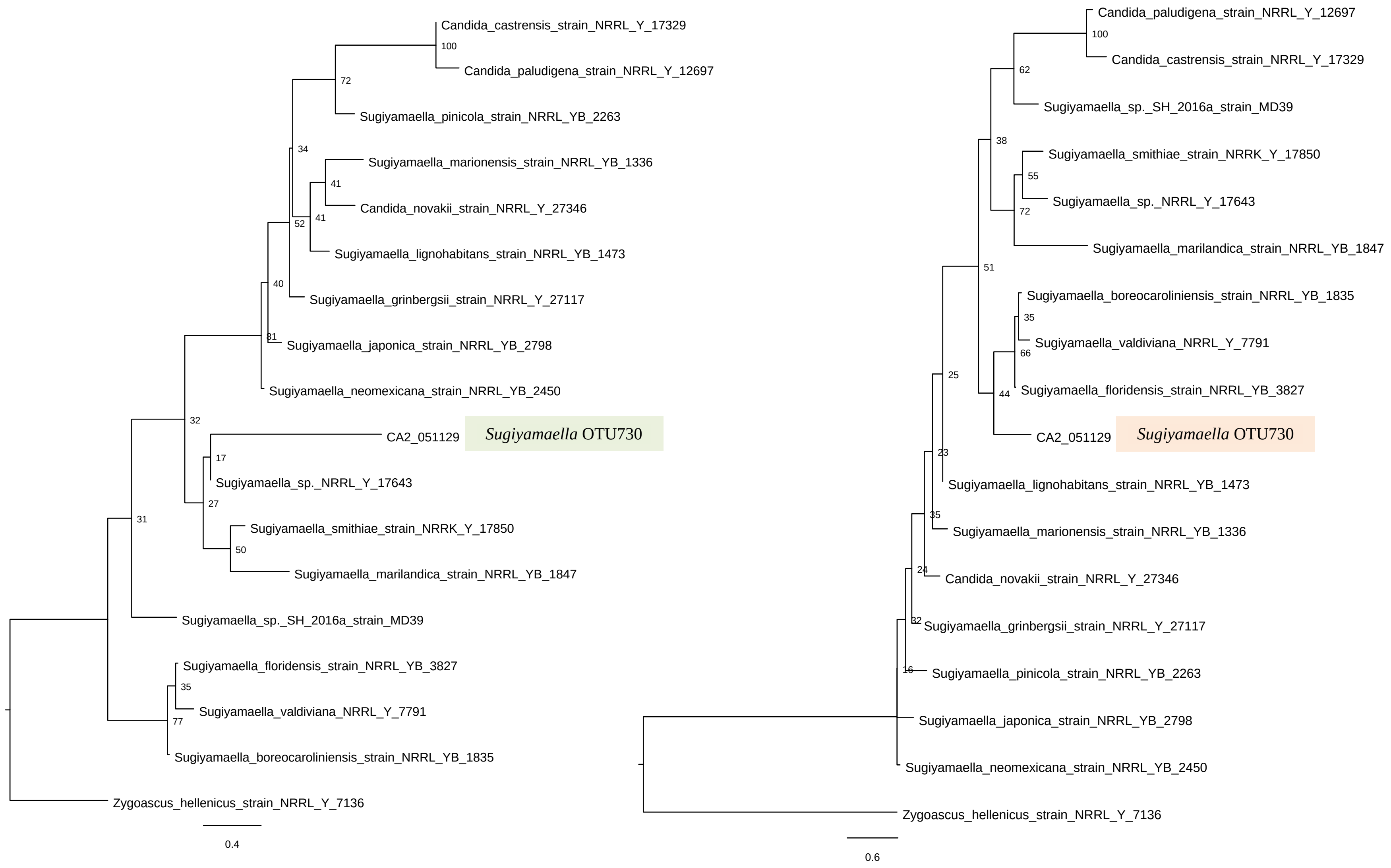

Figure S22. Phylograms of *Sugiyamaella* OTU730 (Saccharomycetes) generated from Randomized Axelerated Maximum Likelihood (RAxML) analysis based on internal transcribed spacer 1 (ITS1, left) or on internal transcribed spacer 2 (ITS2; right) sequence.

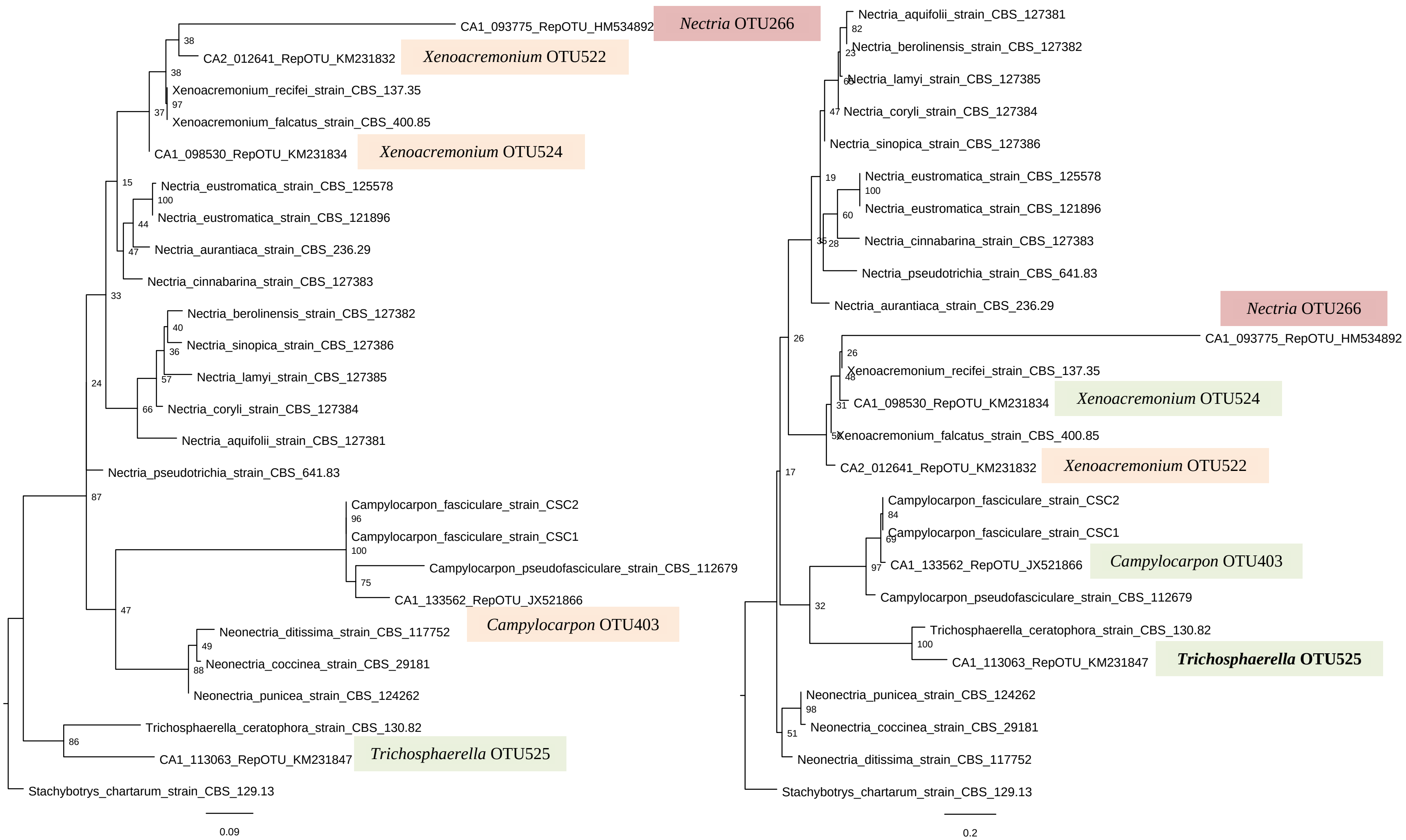

Figure S23. Phylograms of *Campylocarpon* OTU403 (Sordariomycetes), *Xenoacremonium* OTU524, OTU522 (Sordariomycetes), *Nectria* OTU266 (Sordariomycetes), *Trichosphaerella* OTU525 (Sordariomycetes) generated from Randomized Axelerated Maximum Likelihood (RAxML) analysis based on internal transcribed spacer 1 (ITS1, left) or on internal transcribed spacer 2 (ITS2; right) sequence.

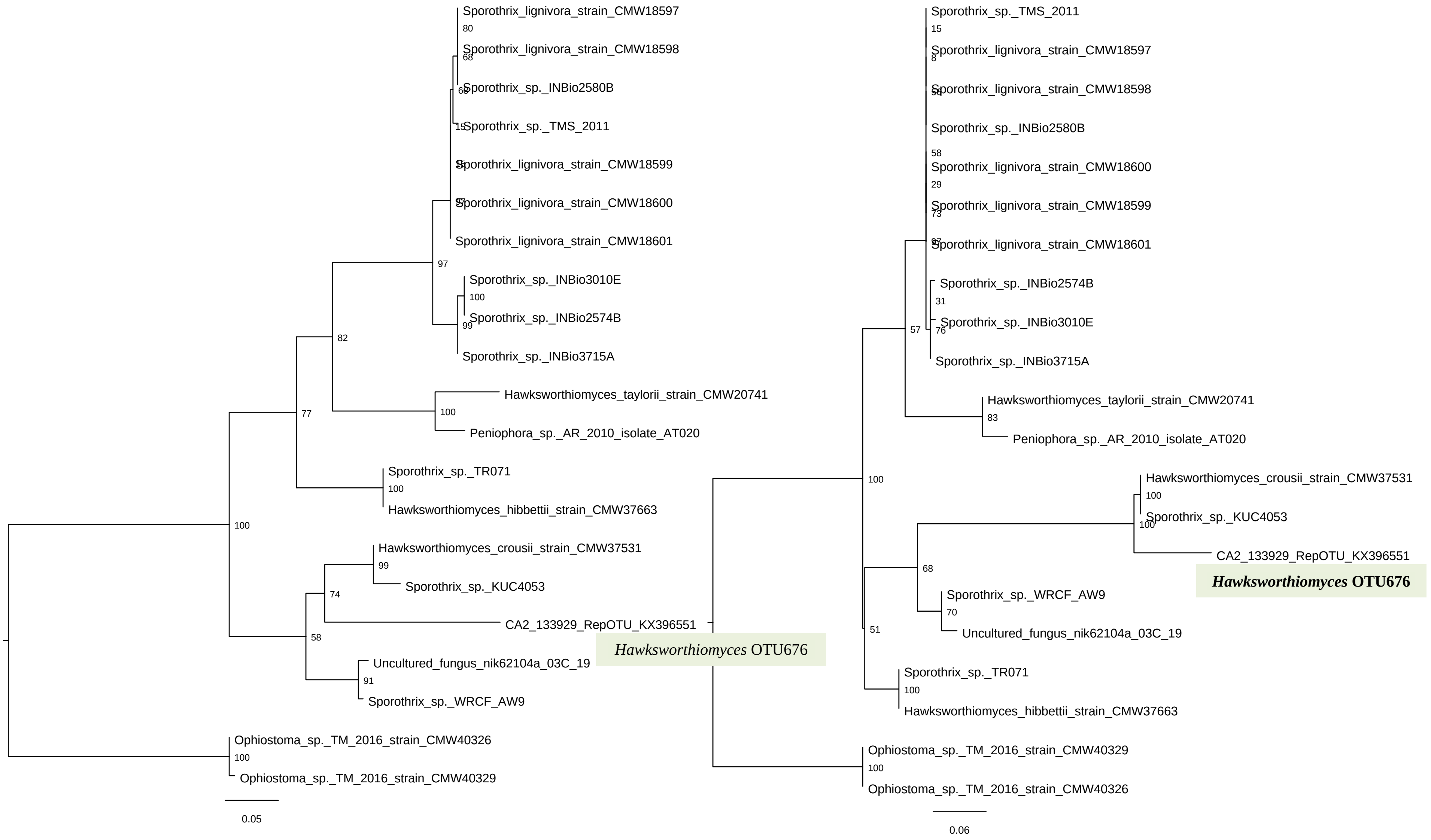

Figure S24. Phylograms of *Hawksworthiomyces* OTU676 (Sordariomycetes) generated from Randomized Axelerated Maximum Likelihood (RAxML) analysis based on internal transcribed spacer 1 (ITS1, left) or on internal transcribed spacer 2 (ITS2; right) sequence.

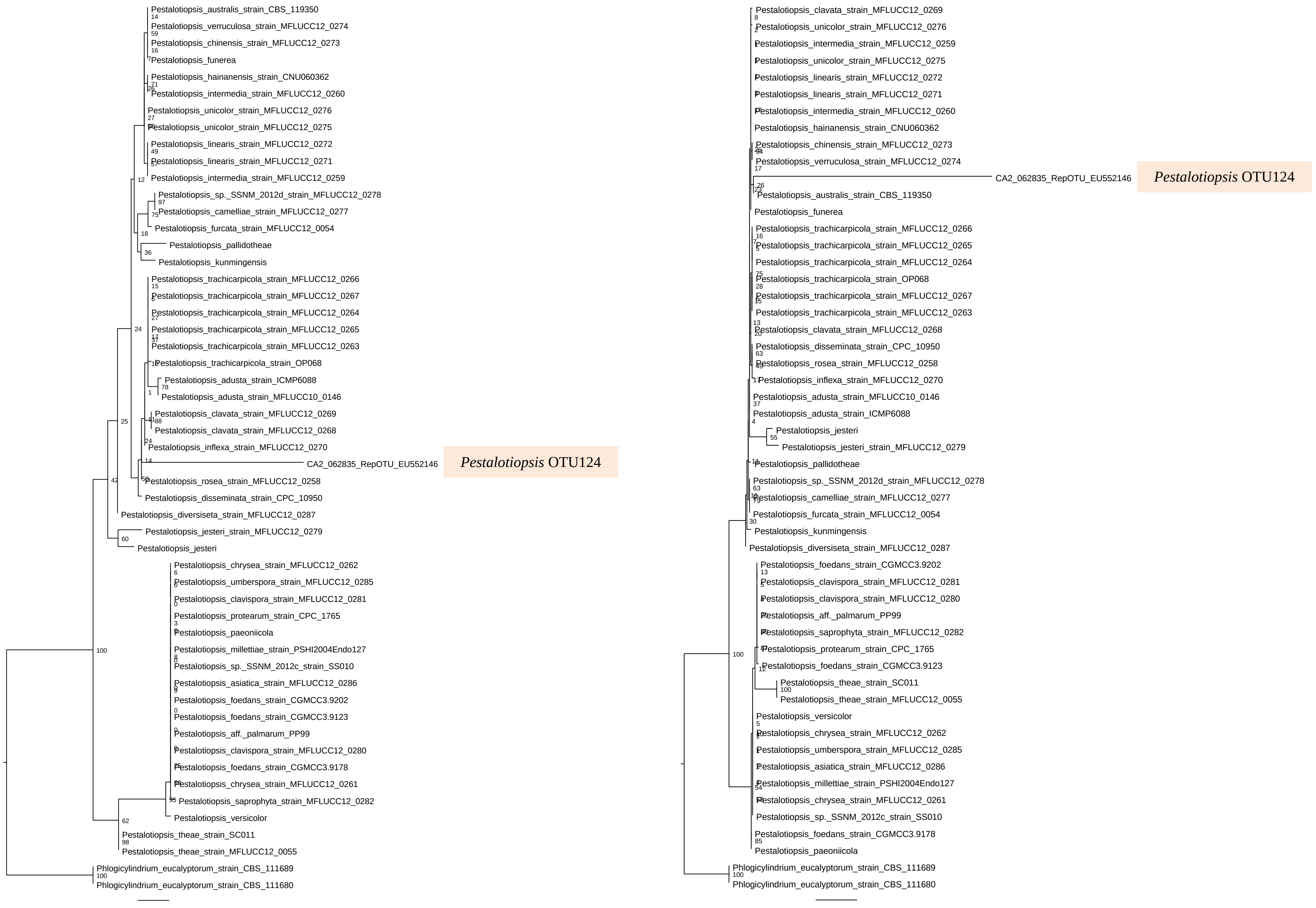

Figure S25. Phylograms of *Pestalotiopsis* OTU124 (Sordariomycetes) generated from Randomized Axelerated Maximum Likelihood (RAxML) analysis based on internal transcribed spacer 1 (ITS1, left) or on internal transcribed spacer 2 (ITS2; right) sequence.

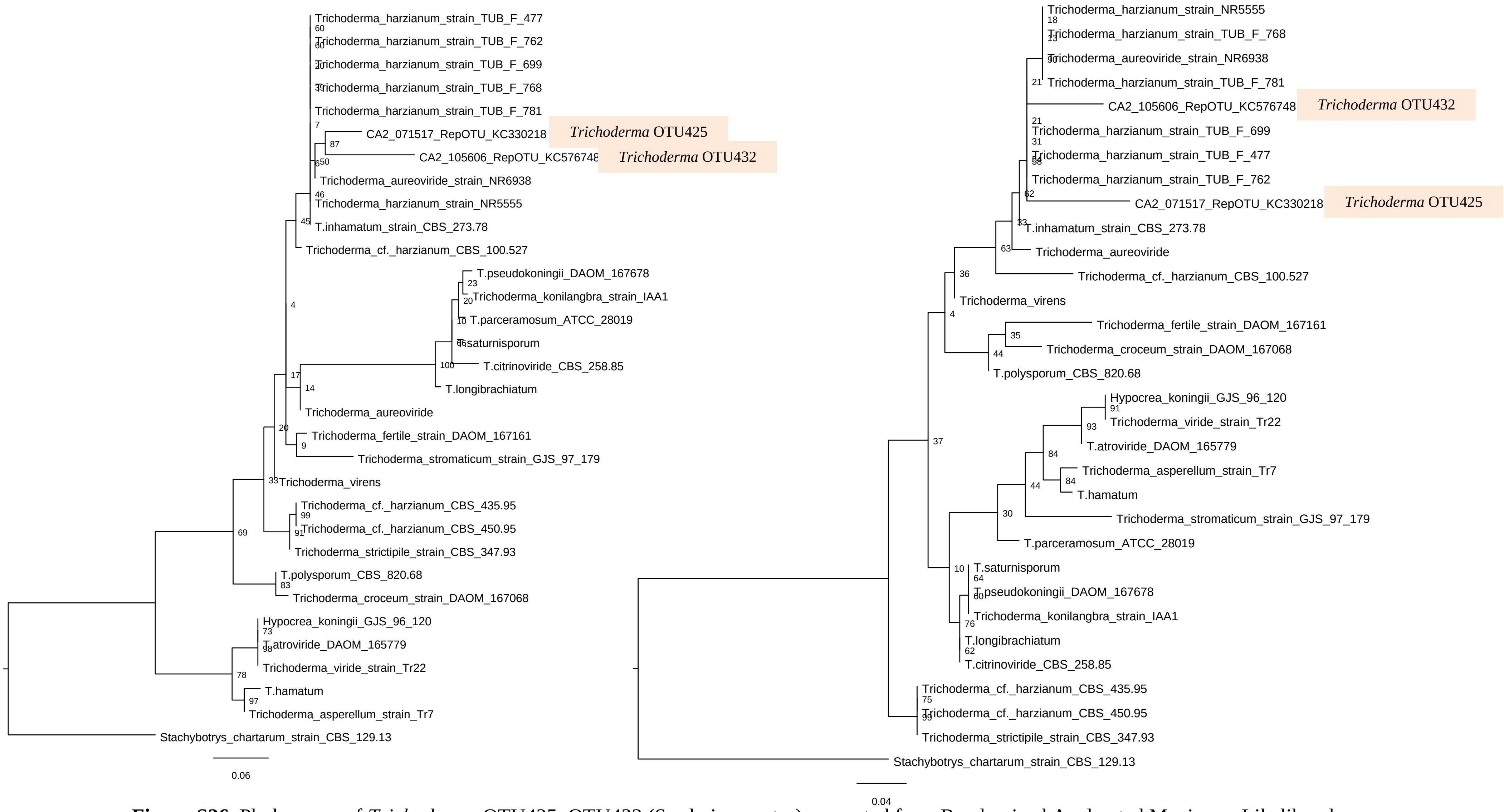

Figure S26. Phylograms of *Trichoderma* OTU425, OTU432 (Sordariomycetes) generated from Randomized Axelerated Maximum Likelihood (RAxML) analysis based on internal transcribed spacer 1 (ITS1, left) or on internal transcribed spacer 2 (ITS2; right) sequence.

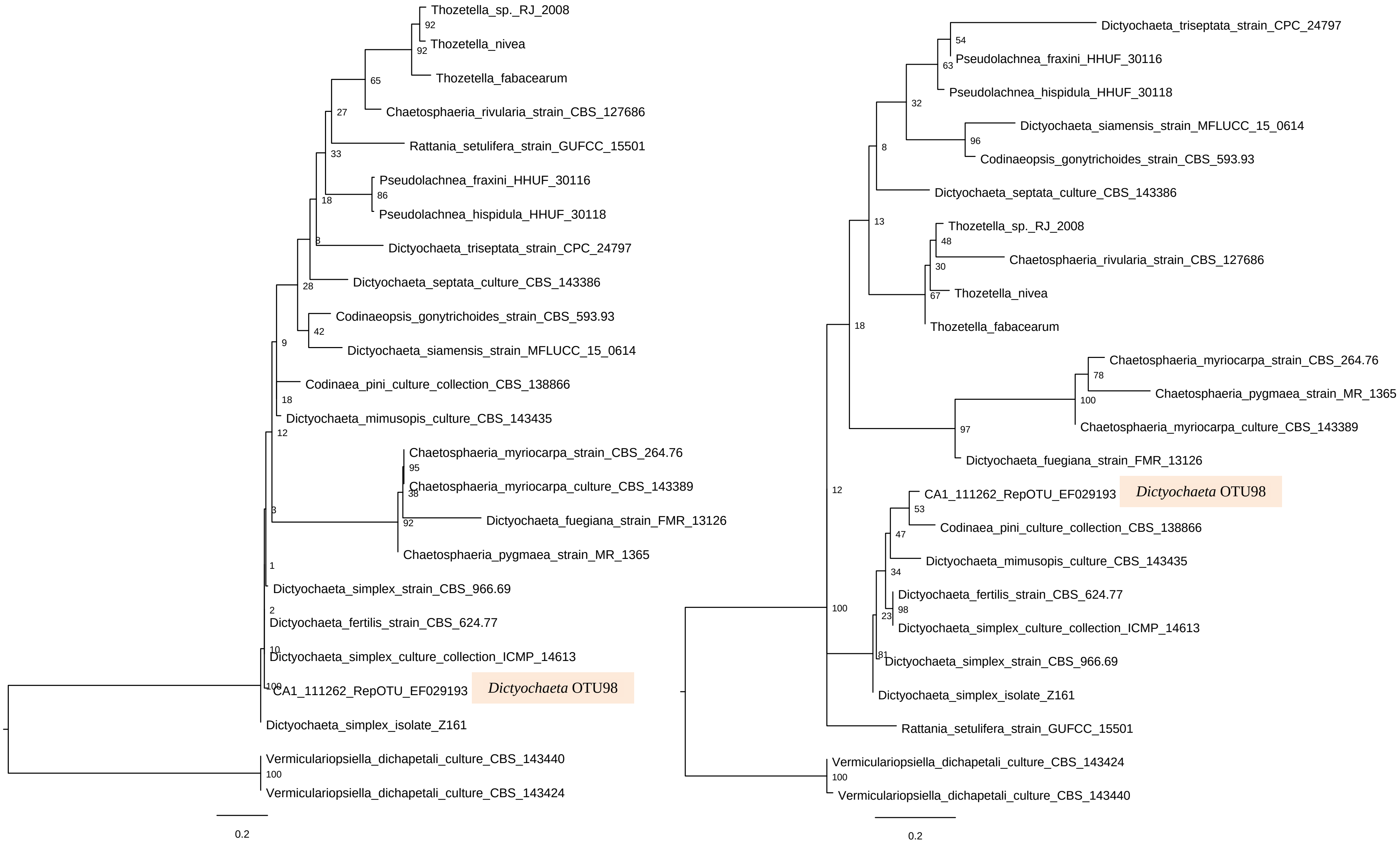

Figure S27. Phylograms of *Dictyochaeta* OTU98 (Sordariomycetes) generated from Randomized Axelerated Maximum Likelihood (RAxML) analysis based on internal transcribed spacer 1 (ITS1, left) or on internal transcribed spacer 2 (ITS2; right) sequence.

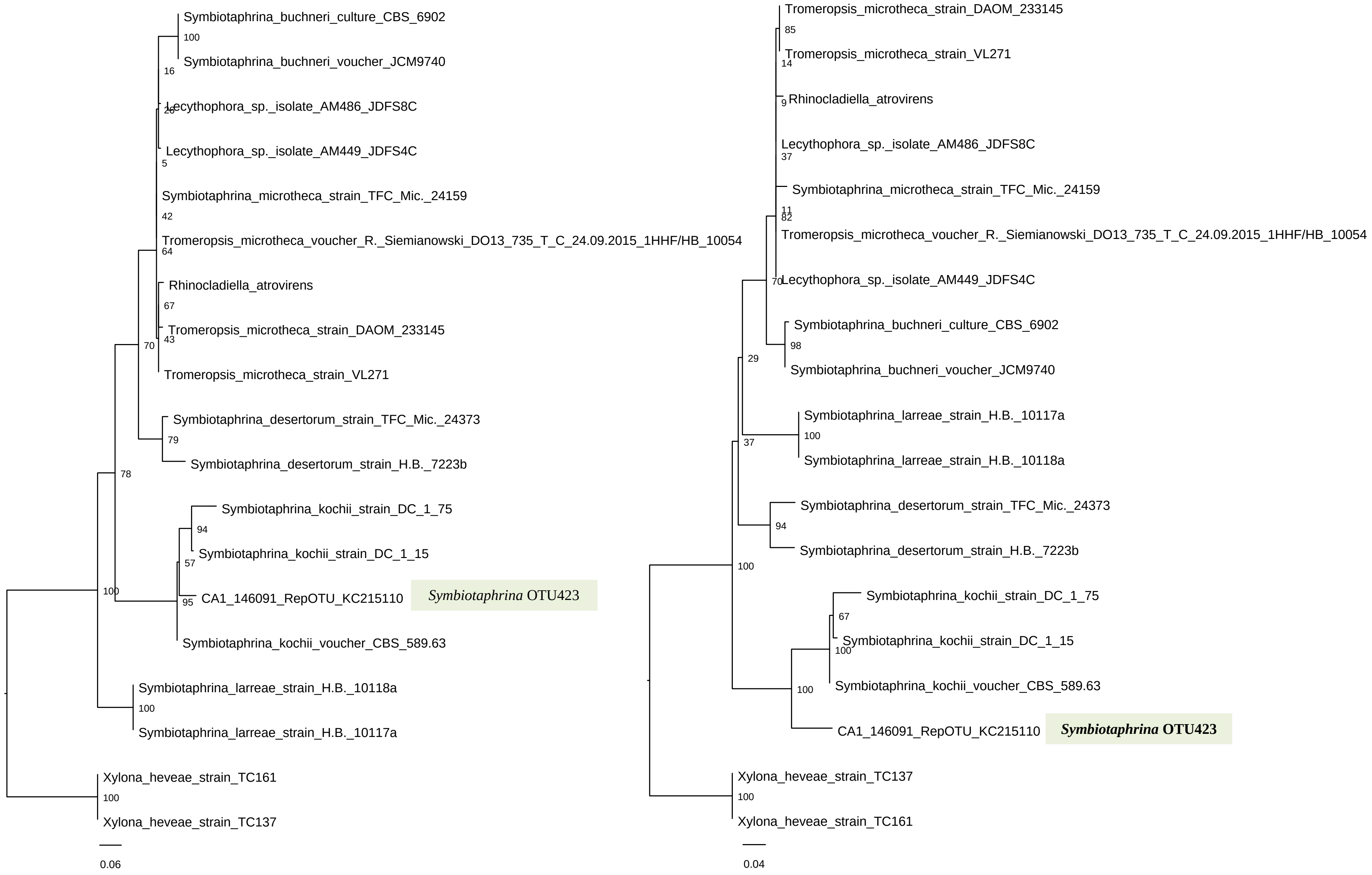

Figure S28. Phylograms of *Symbiotaphrina* OTU423 (Xylonomycetes) generated from Randomized Axelerated Maximum Likelihood (RAxML) analysis based on internal transcribed spacer 1 (ITS1, left) or on internal transcribed spacer 2 (ITS2; right) sequence.

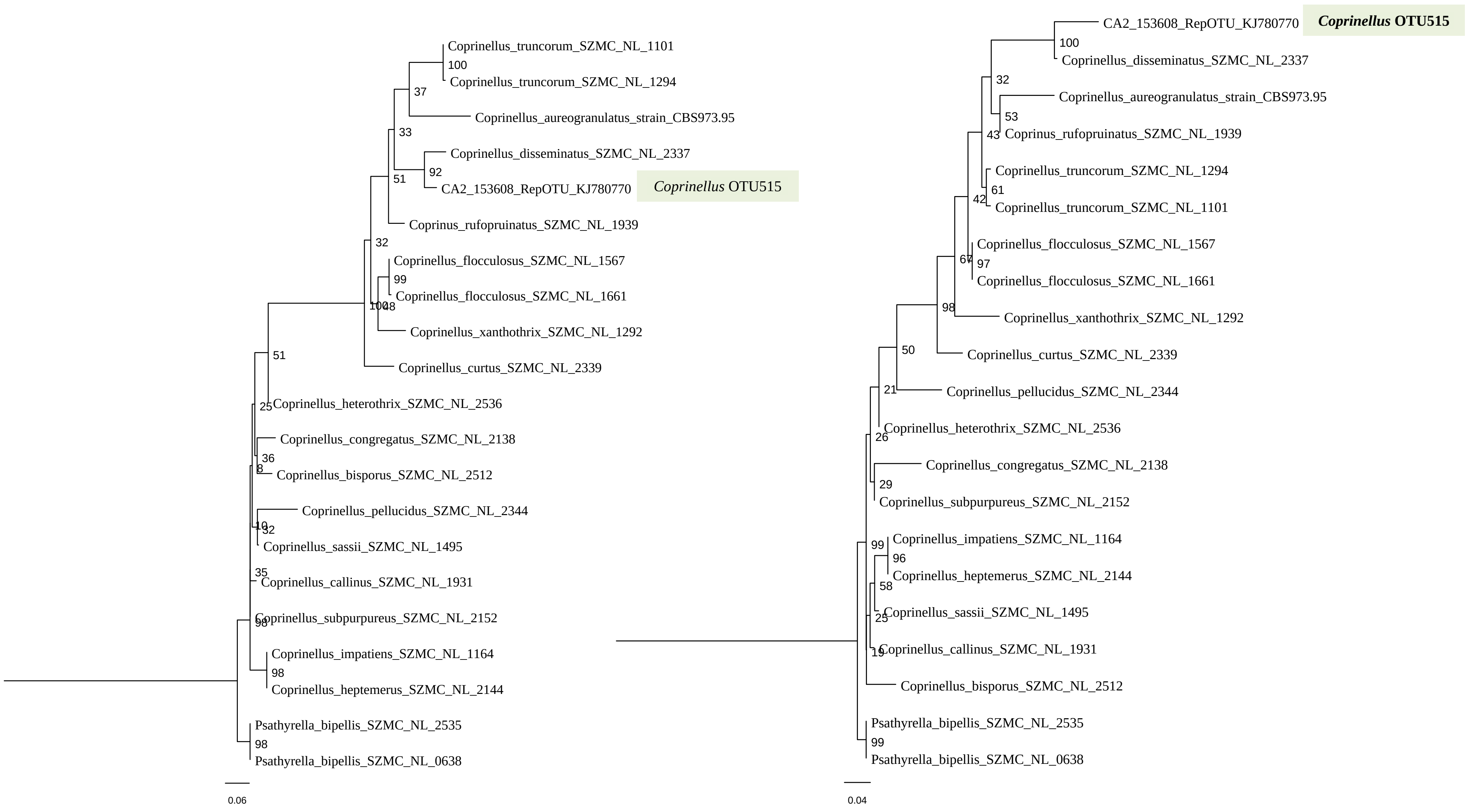

Figure S29. Phylograms of *Coprinellus* OTU515 (Agaricomycetes) generated from Randomized Axelerated Maximum Likelihood (RAxML) analysis based on internal transcribed spacer 1 (ITS1, left) or on internal transcribed spacer 2 (ITS2; right) sequence.

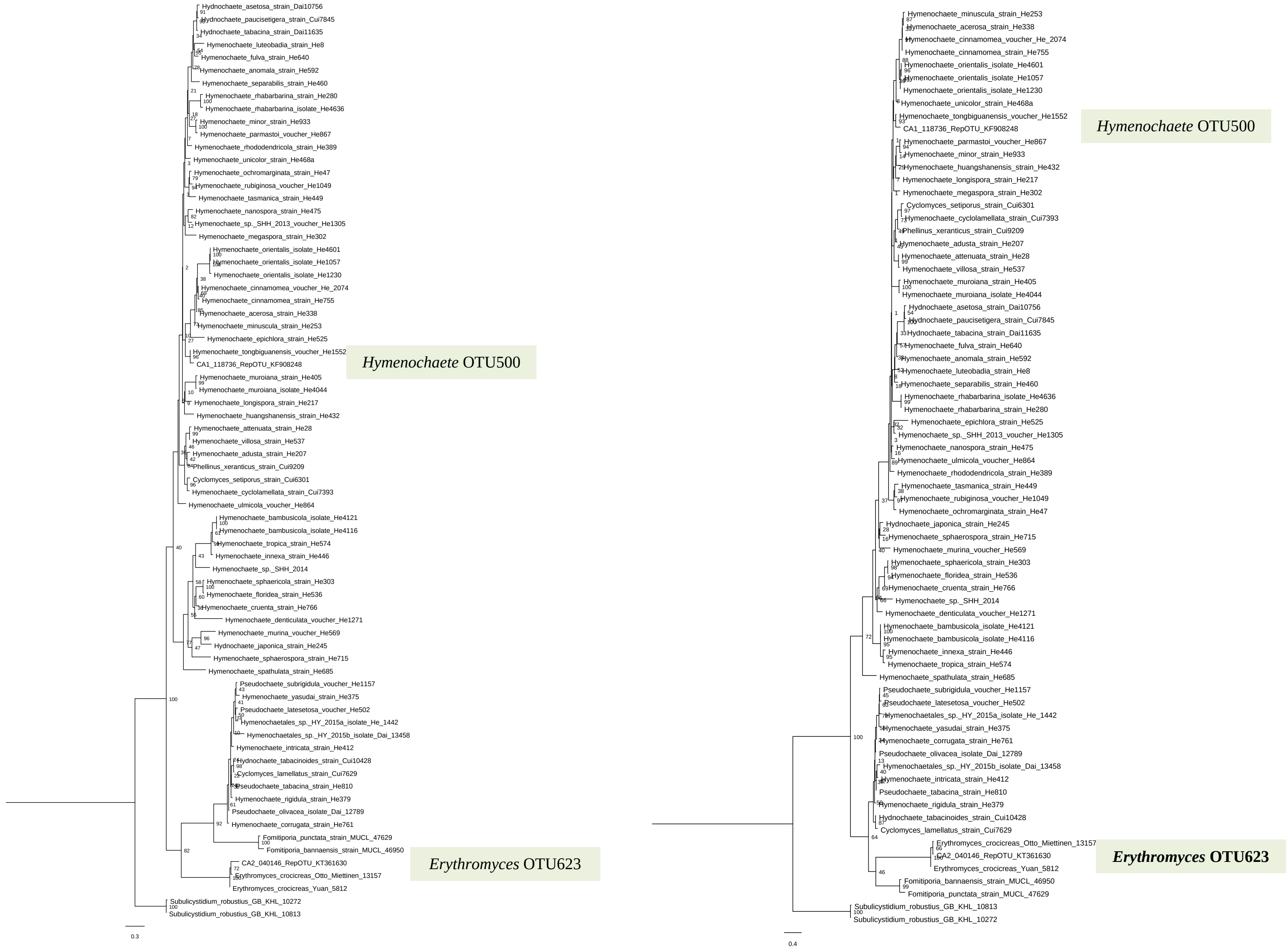

Figure S30. Phylograms of *Hymenochaete* OTU500 (Agaricomycetes), *Erythromyces* OTU623 (Agaricomycetes) generated from Randomized Axelerated Maximum Likelihood (RAxML) analysis based on internal transcribed spacer 1 (ITS1, left) or on internal transcribed spacer 2 (ITS2; right) sequence.

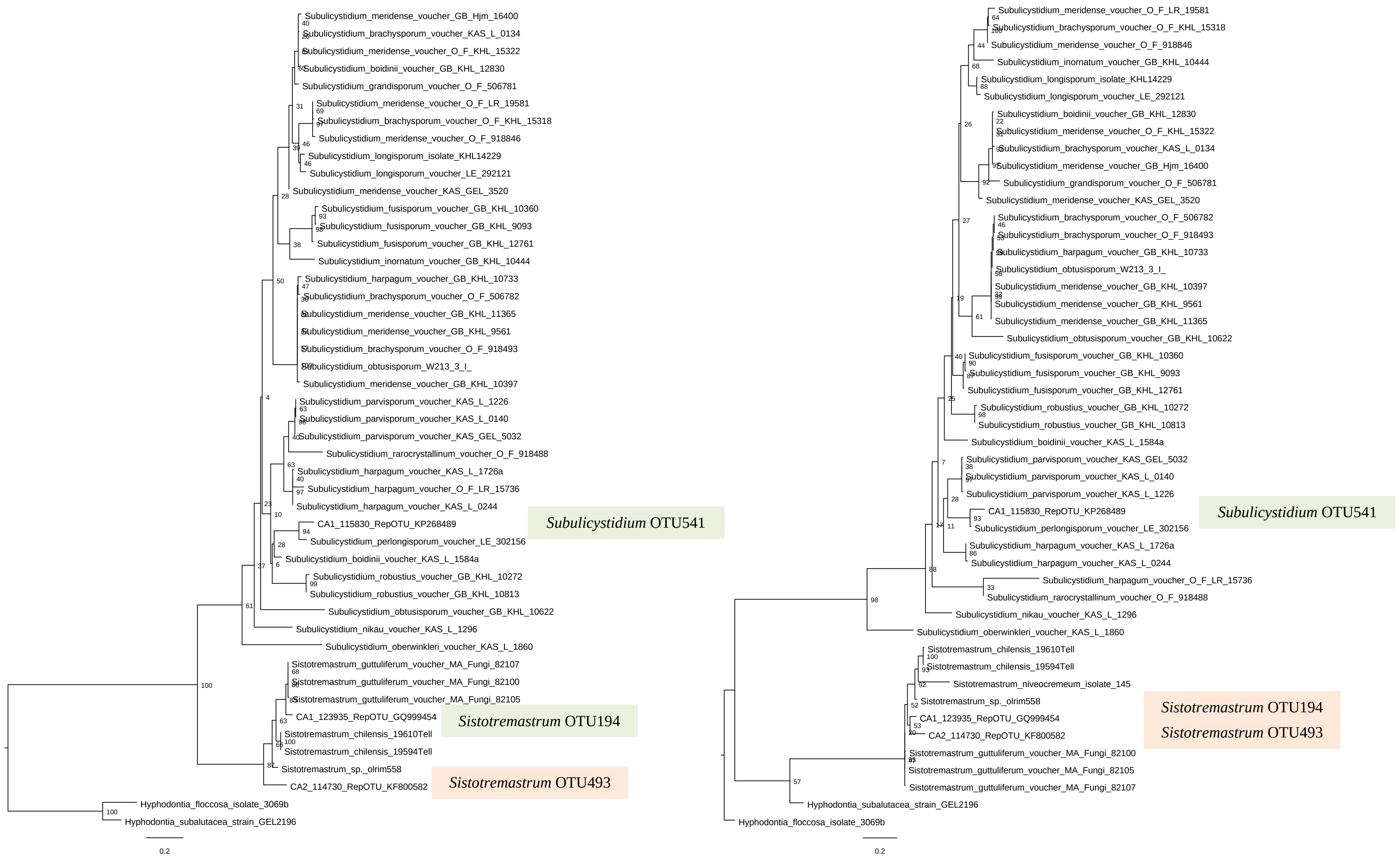

Figure S31. Phylograms of *Subulicystidium* OTU541, *Sistotremastrum* OTU194, OTU493 (Agaricomycetes) generated from Randomized Axelerated Maximum Likelihood (RAxML) analysis based on internal transcribed spacer 1 (ITS1, left) or on internal transcribed spacer 2 (ITS2; right) sequence.

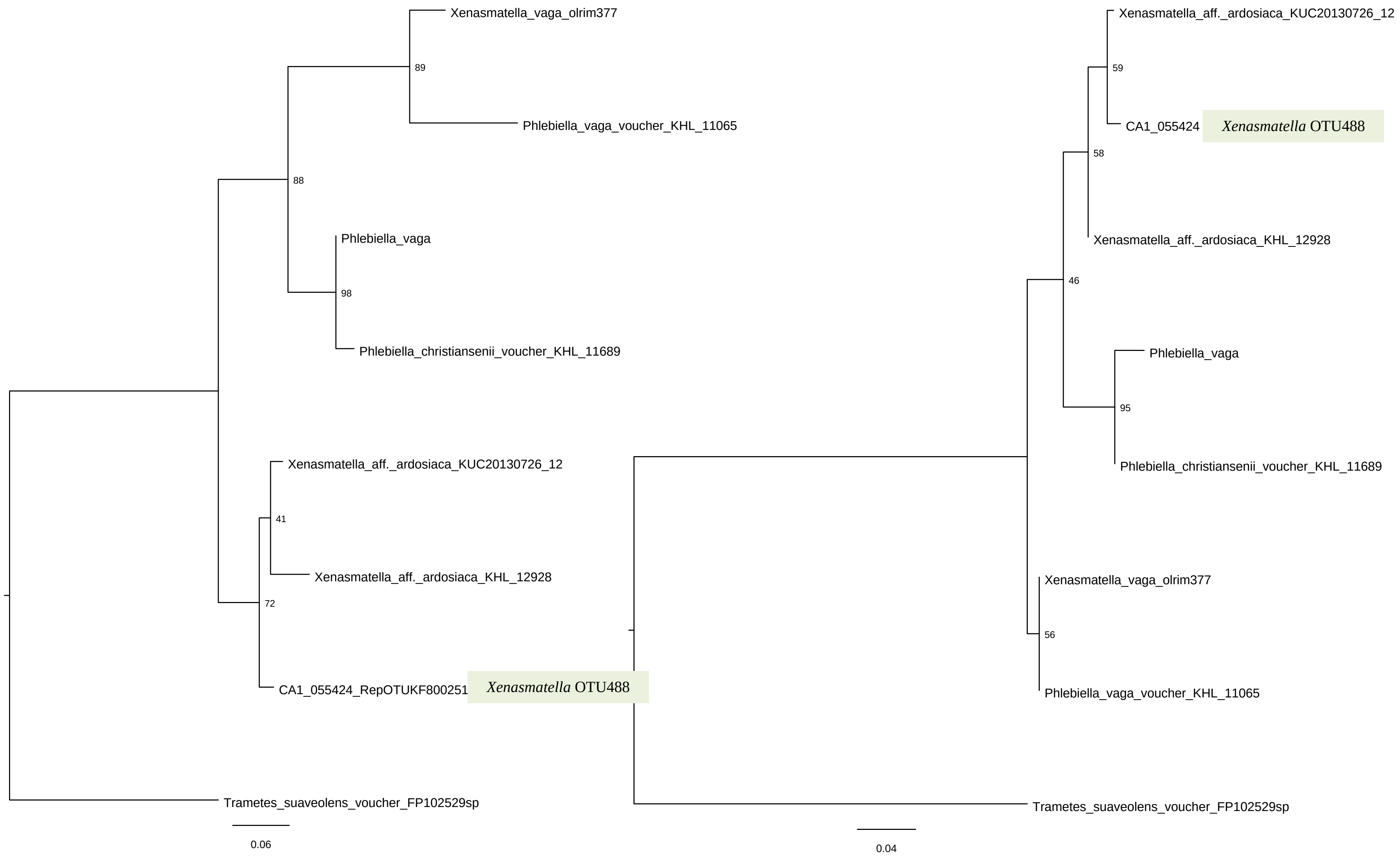

Figure S32. Phylograms of *Xenasmatella* OTU488 (Agaricomycetes) generated from Randomized Axelerated Maximum Likelihood (RAxML) analysis based on internal transcribed spacer 1 (ITS1, left) or on internal transcribed spacer 2 (ITS2; right) sequence.

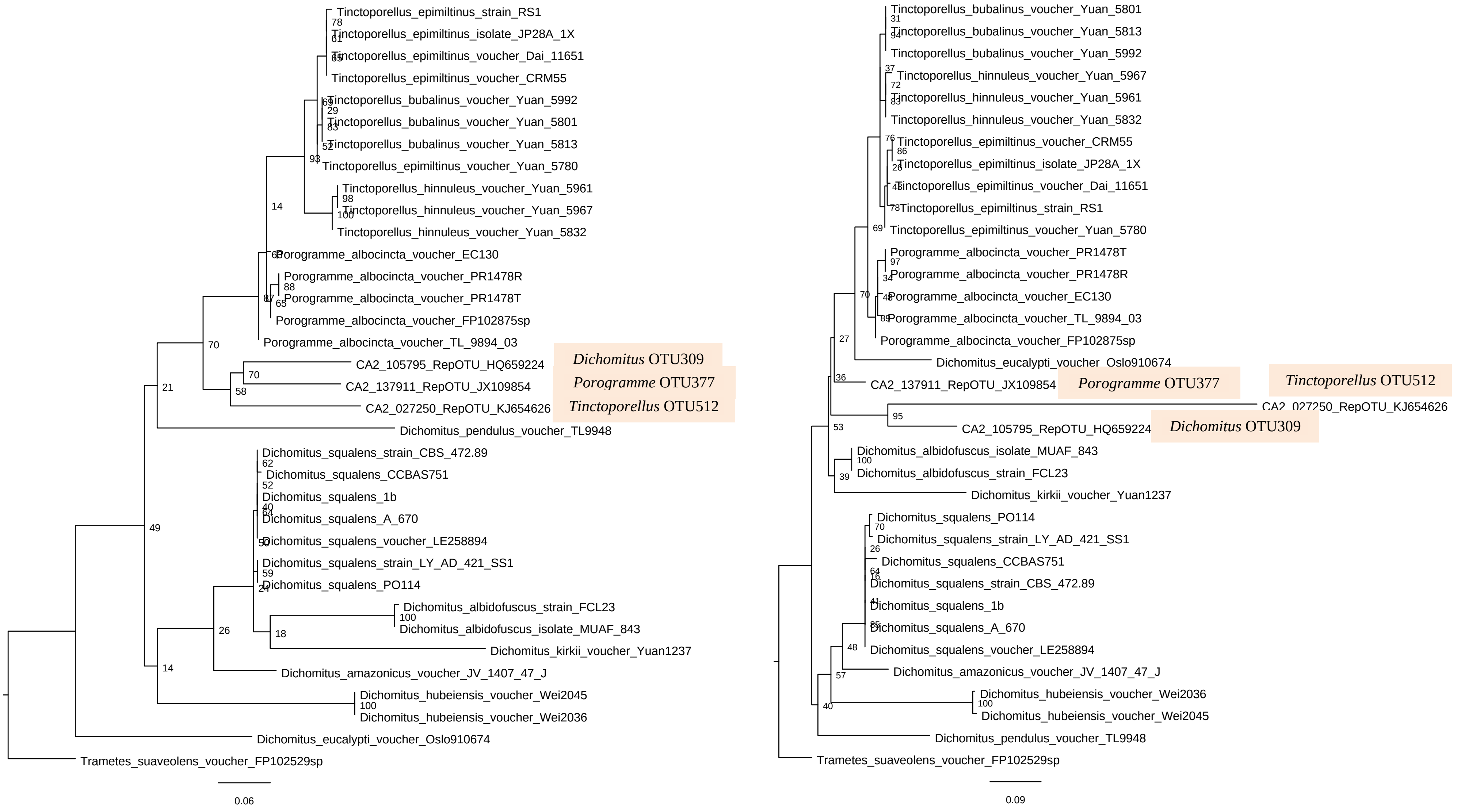

Figure S33. Phylograms of *Dichomitus* OTU309 (Agaricomycetes), *Porogramme* OTU377 (Agaricomycetes), *Tinctoporellus* OTU512 (Agaricomycetes) generated from Randomized Axelerated Maximum Likelihood (RAxML) analysis based on internal transcribed spacer 1 (ITS1, left) or on internal transcribed spacer 2 (ITS2; right) sequence.

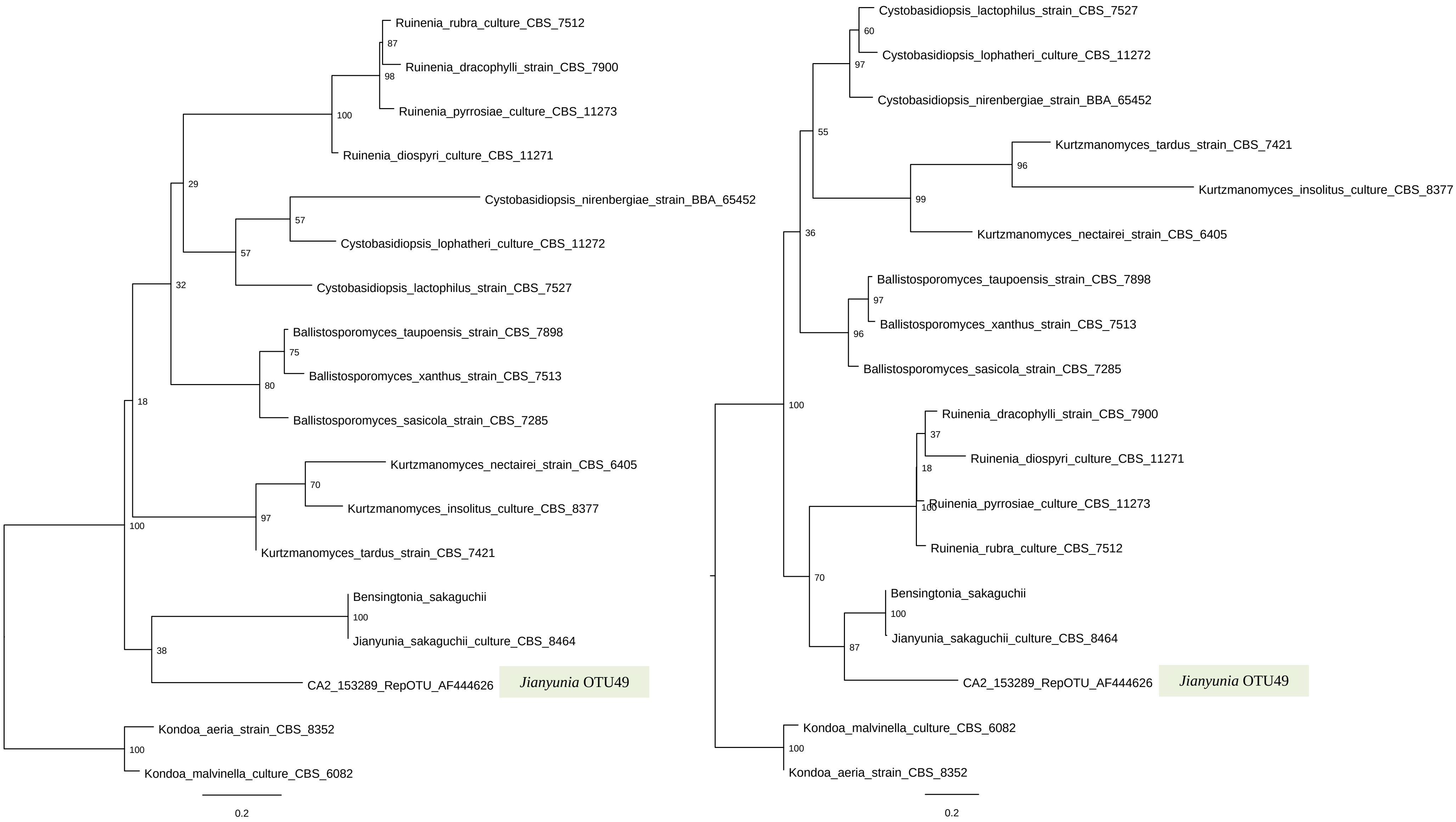

Figure S34. Phylograms of *Jianyunia* OTU49 (Agaricomycetes) generated from Randomized Axelerated Maximum Likelihood (RAxML) analysis based on internal transcribed spacer 1 (ITS1, left) or on internal transcribed spacer 2 (ITS2; right) sequence.

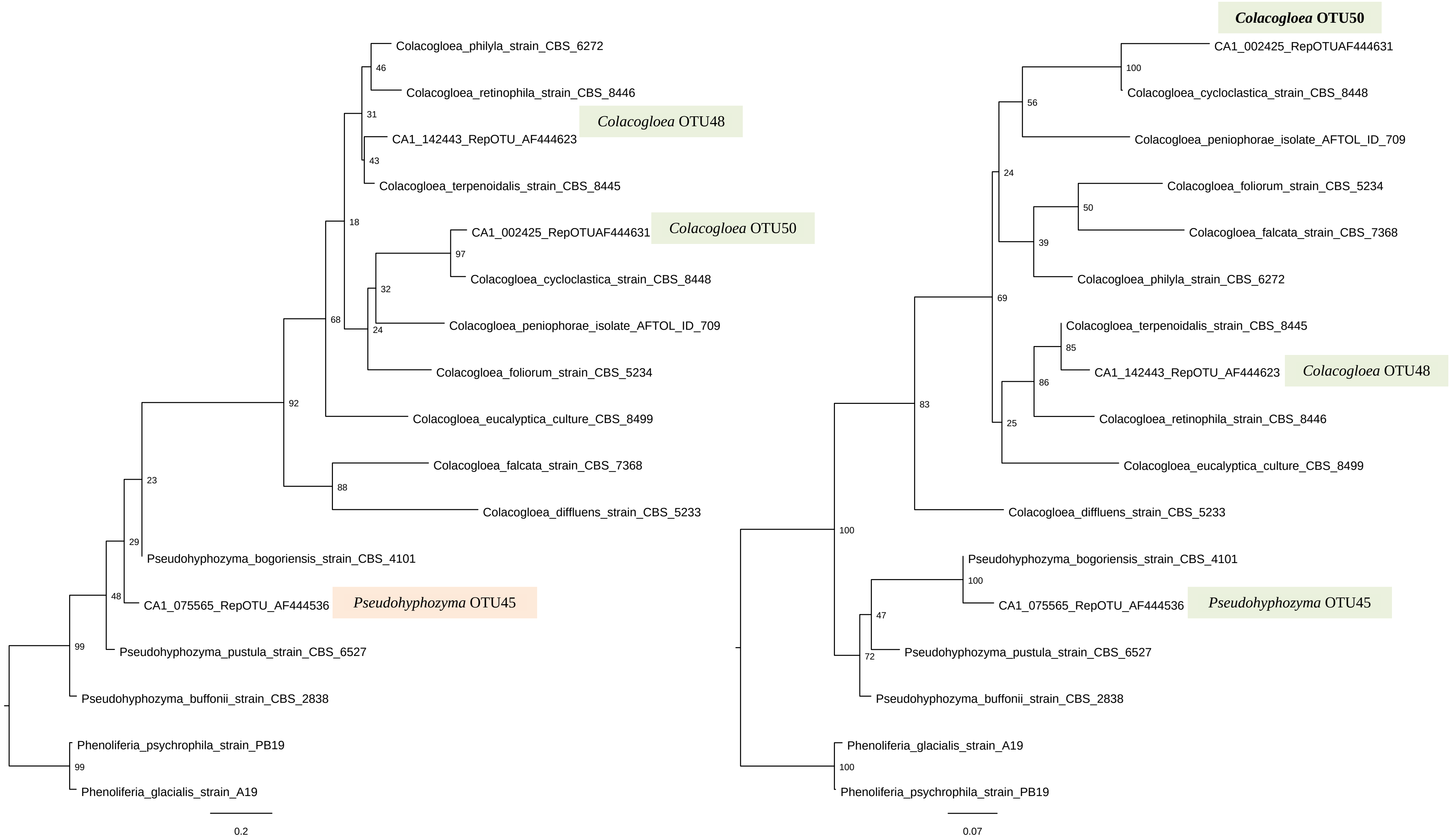

Figure S35. Phylograms of *Colacogloea* OTU48, OTU50 (Microbotryomycetes), *Pseudohyphozyma* OTU45 (Microbotryomycetes) generated from Randomized Axelerated Maximum Likelihood (RAxML) analysis based on internal transcribed spacer 1 (ITS1, left) or on internal transcribed spacer 2 (ITS2; right) sequence.

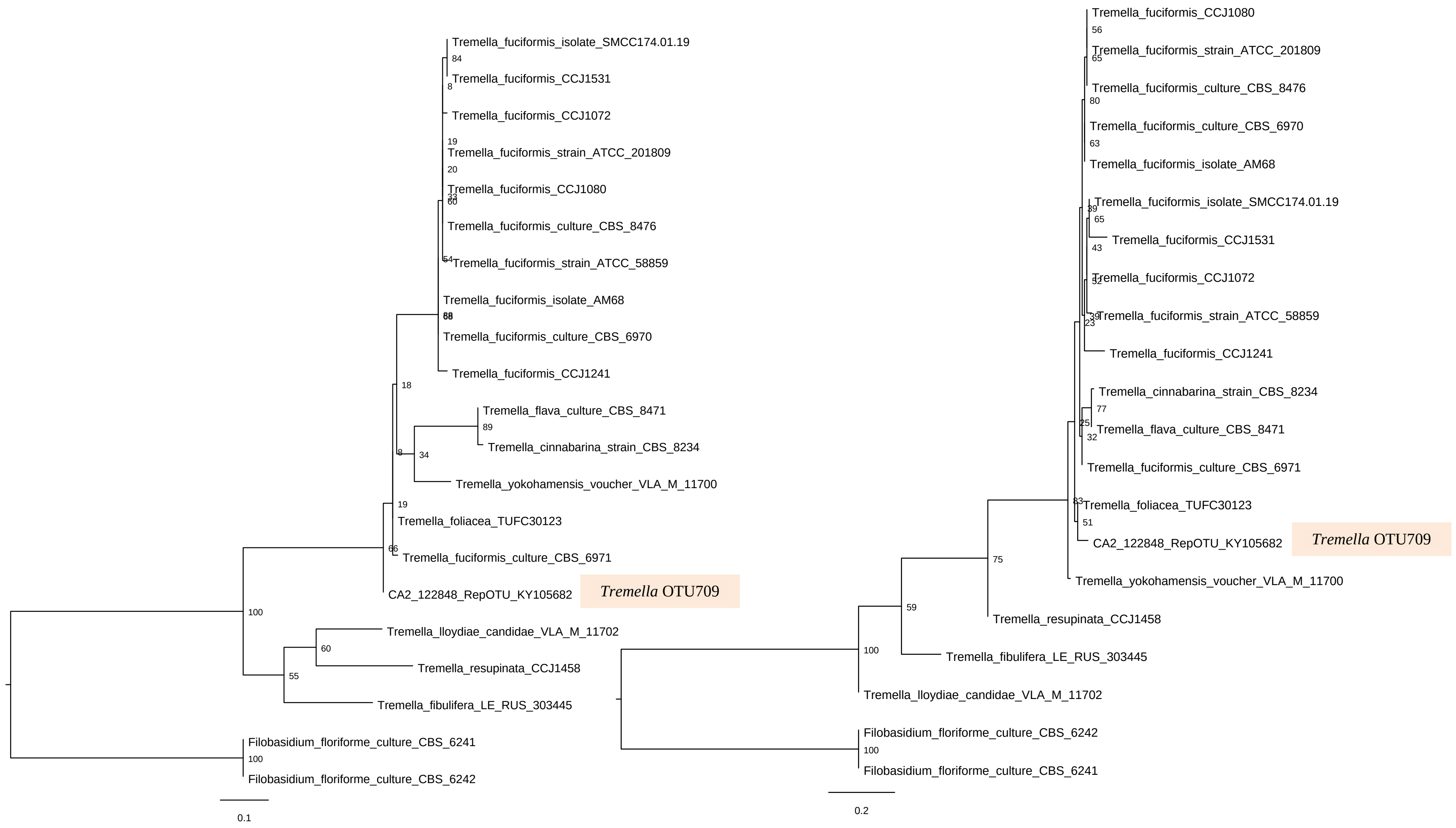

Figure S36. Phylograms of *Tremella* OTU709 (Tremellomycetes) generated from Randomized Axelerated Maximum Likelihood (RAxML) analysis based on internal transcribed spacer 1 (ITS1, left) or on internal transcribed spacer 2 (ITS2; right) sequence.
